# Probing Peptidylprolyl Bond *cis*/*trans* Status Using Distal ^19^F NMR Reporters

**DOI:** 10.1002/chem.202203017

**Published:** 2023-02-16

**Authors:** Patrick M. Killoran, George S. M. Hanson, Sanne J. M. Verhoork, Madeleine Smith, Davide Del Gobbo, Lu‐Yun Lian, Christopher R. Coxon

**Affiliations:** ^1^ School of Pharmacy and Biomolecular Sciences Liverpool John Moores University Liverpool Merseyside L3 3AF UK; ^2^ Institute of Systems Molecular and Integrative Biology The University of Liverpool Crown Street Liverpool L69 7ZB UK; ^3^ EaStChem School of Chemistry The University of Edinburgh Joseph Black Building David Brewster Road Edinburgh EH14 4AS UK

**Keywords:** fluorinated amino acids, ^19^F NMR, peptides, proline conformation

## Abstract

A method for measuring peptidylprolyl bond *cis‐trans* conformational status in peptide models is described, using 4‐fluorophenylalanine (4FPhe) as a distal reporter for ^19^F NMR. The %*cis*‐Pro population was measured for peptides of the general structure Ac‐X‐Pro‐Z‐Ala‐Ala‐4FPhe (X and Z are proteinogenic amino acids) at pH 7.4, and provided conformational populations consistent with literature values obtained by more complex methods. This approach was applied to probe the prolyl bond status in pentapeptide models of the intrinsically disordered C‐terminal region of α‐synuclein, which mirrored the preferences in the Ac‐X‐Pro‐Z‐Ala‐4FPhe models. Advantageously, the ^19^F reporter group does not need to be adjacent to or attached to proline to provide quantifiable signals and distal 4‐fluorophenylalanines can be placed so as not to influence prolyl bond conformation. Finally, we demonstrated that the prolyl bond status is not significantly affected by pH when there are ionisable amino acid residues at the carboxyl side of proline, which makes ^19^F NMR an invaluable tool with which to study proline isomerism at a range of pHs and in different solvents and buffers.

## Graphical abstract

**It is demonstrated that incorporation** of 4‐fluorophenylalanine in model peptides provides an accurate and non‐biased reporter of peptidylprolyl *cis‐trans* conformational status by ^19^F NMR at physiological pH, even when several residues away from proline. In simple models, the prolyl bond conformational populations are predominantly governed by the amino acids local to proline and are conserved across different model systems.

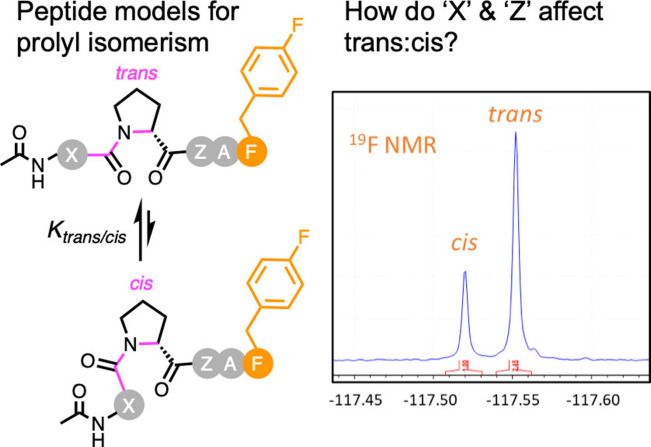

## Introduction

**Figure 1:**
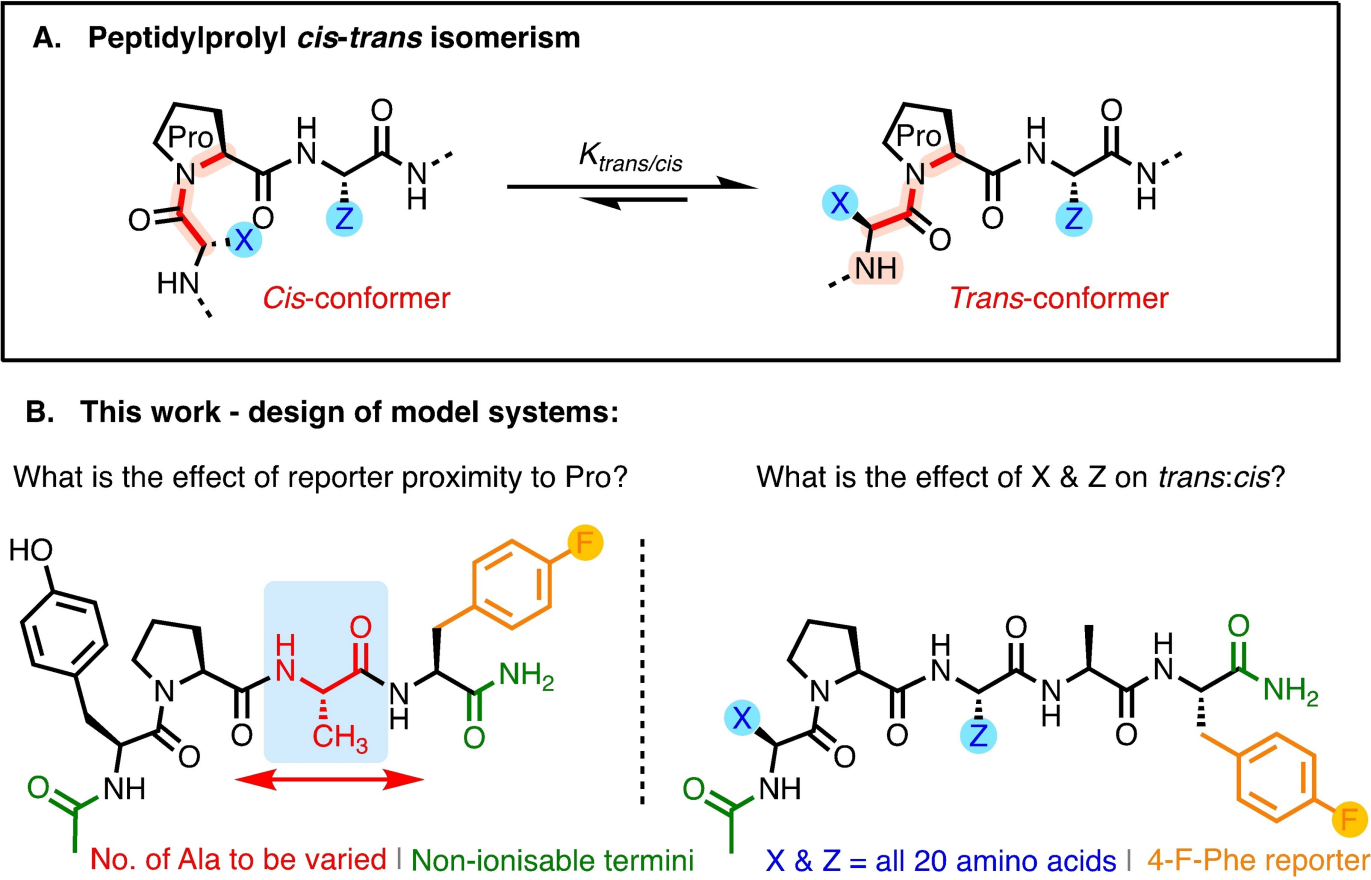
Peptidylprolyl *cis*‐*trans* isomerism and the design of model systems containing fluorine reporter groups for ^19^F NMR to measure prolyl bond status.

The peptidylprolyl bond is a key facilitator of conformational change and peptidylprolyl bond *cis‐trans* isomerism is often the slow‐phase or rate‐limiting step in protein folding, with a ∼80 kJ/mol energy barrier for interconversion.^[1]^ Standard backbone amide bonds, comprised of non‐cyclic canonical amino acids have a very high thermodynamic preference for a *trans* conformation.^[2]^ However, with its sidechain forming part of the backbone, proline contains a tertiary amide in peptides and proteins, which makes the difference in energy between the *cis* and *trans* forms lower than for standard amino acids, affording a higher population of the *cis* conformation (Figure 1A) at proline compared with non‐prolines. These features result in unique properties that are fundamental to the three‐dimensional structure of peptides and proteins. Indeed, the prevalence of proline residues in intrinsically disordered protein motifs (approx. double the average^[3]^) has prompted further investigation into the role of prolines in adopting partially folded states and promoting disorder or compaction.^[4]^ Indeed, the peptidylprolyl *cis‐trans* isomerases CypD and FK506 binding proteins (FKBPs) have been shown to interact directly with the acidic proline‐rich C‐terminal region of α‐synuclein (αSyn), which forms a constituent part of the protein aggregates in Lewy bodies in a range of synucleinopathies, for example, Parkinsons Disease.[^5^, ^6^] FKBPs were shown to accelerate the rate of aggregation, and aggregation was abolished by removal of the proline residues from the C‐terminus. However, CypD binding to soluble αSyn prevented its aggregation, whilst the addition of CypD to preformed αSyn fibrils led to fibril disassembly. In both cases, this behaviour was linked to an increased rate of isomerase‐catalysed peptidylprolyl isomerisation. Moreover, Pro‐to‐Ala mutations at C‐terminal region are reported to accelerate the rate of uncatalysed aggregation,^[7]^ highlighting a key role of proline conformation in Parkinson's Disease pathology.

The natural populations of specific prolyl bond conformers is affected by i) the presence of electron‐donating/withdrawing or bulky groups at the proline 3‐ or 4‐positions, which controls the pyrrolidine *endo*/*exo* ring pucker through steric and/or stereoelectronic effects,^[8]^ and ii) the nature of neighbouring amino acid sidechains, and that these trends are consistent with the observed *cis*‐populations in fully folded proteins.[^9^, ^10^, ^11^, ^12^, ^13^]

Most of the studies described have used NMR‐based approaches to detect and/or quantify prolyl conformers. NMR techniques provide structural information at the molecular level and can be useful in understanding the finer details of protein and peptide folding at specific sites, particularly with respect to proline isomerism.[^14^, ^15^] Due to the relatively slow exchange rates between *cis* and *trans* conformers, ^1^H NMR analysis of X‐Pro systems typically affords two sets of signals, allowing the measurement of *cis*:*trans*.[^9^, ^10^, ^11^, ^12^, ^16^] However, this can create complexity in the analysis and in many cases, 2D NMR approaches are required to resolve specific resonances. Compared with the use of ^1^H NMR for studies of peptide conformation, ^19^F NMR exhibits very little‐to‐no spectral crowding and will detect separate signals for the different forms that are in chemical exchange. The fluorine‐19 isotope (^19^F) exhibits higher local environmental sensitivity compared with the ^1^H nucleus, providing defined and distinct chemical shifts for individual fluorinated molecules, isomers or conformers.^[17]^ In addition, it is only slightly less sensitive compared with the ^1^H nucleus. Therefore, ^19^F NMR analysis of fluorinated peptides enables far simpler identification and quantification of conformer populations and over a wider range of timescales as compared with traditional methods.

The use of a variety of fluorinated amino acids in conjunction with ^19^F NMR is a valuable tool to study biological processes and several applications have been reported.[^18^, ^19^, ^20^, ^21^] Prolyl bond conformational status in proteins has been measured previously using a combination of fluorinated prolines and ^19^F NMR. In particular, a number of groups have used fluorinated amino acids derivatives to study *cis*‐*trans* isomerism by ^19^F NMR. Thomas and colleagues studied 3*S* and 3*R*‐fluoroproline measured the kinetic and thermodynamic parameters of proline *cis‐trans* isomerisation,^[22]^ whilst Renner performed a similar analysis of 4*S*, 4*R* and 4,4‐difluoroprolines in Ac‐(F)Pro‐OMe using ^19^F NMR.^[23]^ It was clear from this work (and others) that the 4*R*‐fluoroproline has a greater preference for the *trans*‐Pro conformation, whilst the 4*S*‐diastereomer has a relatively increased *cis*‐Pro population compared to the parent proline. These studies highlighted the connection between the fluorine configuration and *cis:trans* conformer populations, albeit these observations were only in only in simple model systems. Kawahara was able to incorporate 4*R*‐fluoroproline into collagen polypeptides and study triple helix assembly and the relationship to proline conformation using ^19^F NMR.^[24]^ Torbeev demonstrated that 4,4‐dIfluoroproline, which has nearly the same *cis*‐*trans* preference as proline but an increased rate of isomerisation, caused pronounced destabilization of macroglobulin; indicating that the *cis‐trans* equilibrium and isomerisation dynamics of a single proline affect the rate of protein folding.^[25]^ Increasing the number of equivalent fluorine atoms enhances the signal‐to‐noise ratio in ^19^F NMR analysis. Trifluoromethylproline was incorporated into a simple Ac‐TfmPro‐OMe model and used with ^19^F NMR to provide an accurate reporter of *cis*:*trans* and to determine the K_*trans/cis*_.^[26]^ Whilst Tressler and co‐workers developed perfluoro‐*tert*‐butyl 4‐hydroxyproline (9 fluorine atoms) as a highly‐sensitive ^19^F NMR reporter for proline isomerism, permitting quantification at concentrations as low as 200 nM.^[27]^ However, these studies also highlighted that fluorine atoms exert an influence on the conformation of prolines, and fluoroproline, is therefore, a biased proline conformational reporter.^[8]^ 4‐Fluorophenylalanine has been used to study the formation of oligomers in the misfolding of prion proteins using ^19^F NMR.^[28]^ Multiple 4‐fluorophenylalanine residues have also been incorporated into a single protein, rat intestinal fatty acid binding protein (IFABP), to monitor the folding process and providing information on the conformational status at various locations simultaneously.^[29]^ 4‐Fluorophenylalanine was also used as a reporter of the enzyme catalysed *cis*‐to‐*trans* isomerisation rate of [4‐fluoro‐Phe]bradykinin measured by magnetisation‐transfer ^19^F NMR.^[30]^ However, most of the reported examples of using 4‐fluorophenylalanine as a ^19^F NMR reporter in peptide and protein systems have focused on its applications in probing global conformational effects and little attention has been paid to its use for probing prolyl bond status.

Here we examine the use of 4‐fluorophenylalanine‐as a reporter to measure the relative *cis:trans* prolyl bond conformer ratio in X‐Pro‐Z systems contained in pentapeptide models. Moreover, we explore how the proximity of this reporter group and the nature of proximal amino acids impacts upon peptidylprolyl *cis*:*trans* populations (Figure 1B).

## Results and Discussion

Employing 4‐fluorophenylalanine as a reporter group, the initial aim was to understand how the proximity of the 4‐fluorophenylalanine residue to the proline, affected the relative *cis*:*trans* ratio and how far away this could be moved within a peptide sequence and still provide distinguishable ^19^F NMR resonances for each conformer. The Tyr‐Pro motif exhibits a relatively high *cis*‐Pro population of around 20–39 % in, for example, Ac‐Ala‐**Tyr**‐**Pro**‐Ala‐Lys‐NH_2_,^[9]^ Ac‐Gly‐**Tyr**‐**Pro**‐Gly^[12]^ and Ac‐Thr‐**Tyr**‐**Pro**‐Asn,^[13]^ depending on other proximal residues.[^9^, ^12^, ^13^] Therefore, this was used as a starting point for model design and optimisation, and a series of peptides containing Ac‐Tyr‐Pro‐(Ala)_n_‐4FPhe (where n=0–7) were synthesised using standard microwave‐assisted solid phase peptide synthesis, sequentially moving the 4‐fluorophenylalanine reporter one residue at a time away from proline. Peptides were prepared as C‐terminal amides and were N‐terminally capped as an acetamide to avoid ionisable termini influencing the *cis*:*trans* through electrostatic interactions with one another or ionisable sidechains, as noted in other reports.[^31^, ^32^]

**Figure 2:**
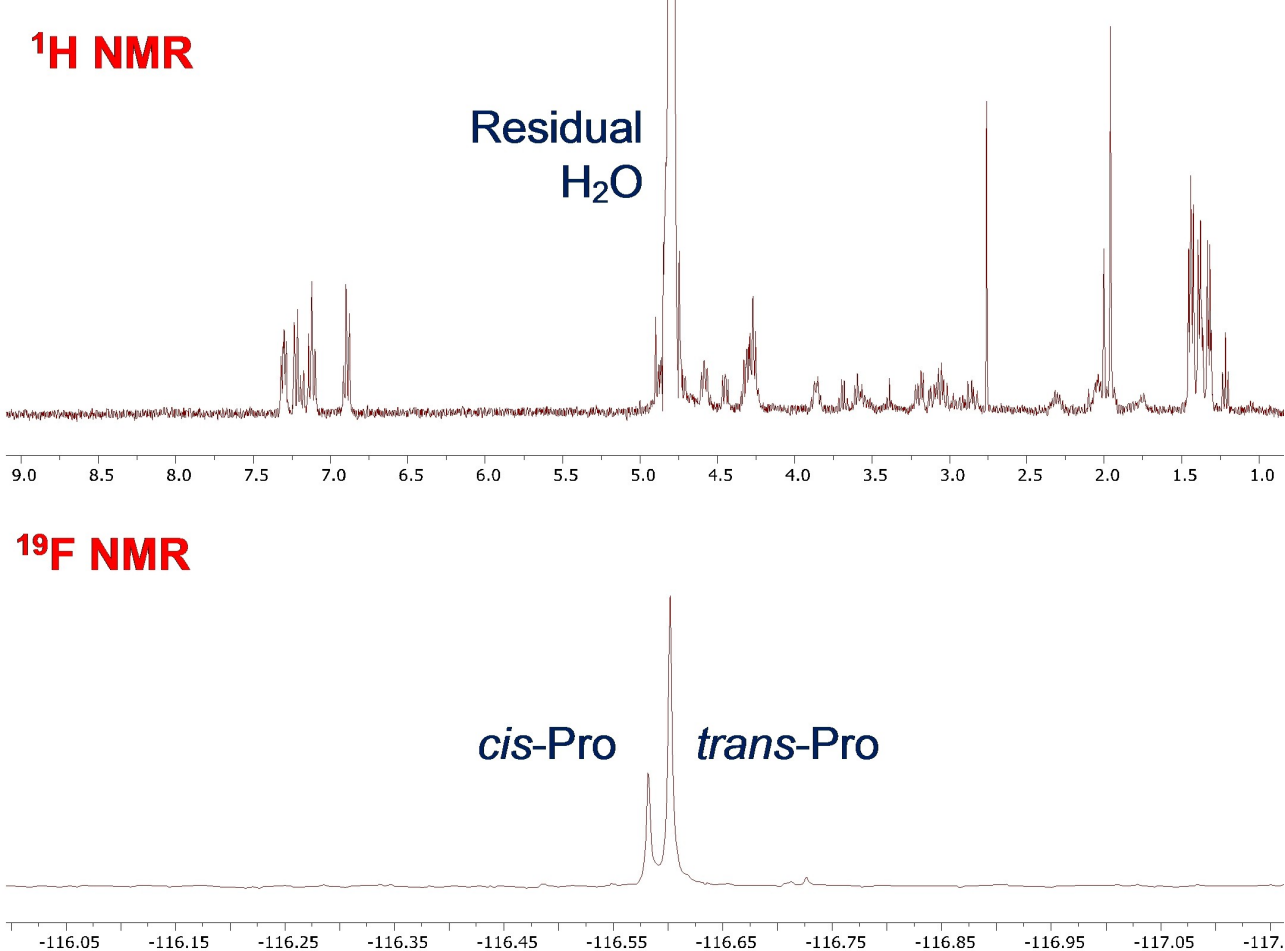
Comparaison of an example ^1^H NMR (top) and ^19^F NMR (bottom) spectrum of peptide Ac‐Tyr‐Pro‐Ala‐Ala‐Ala‐4FPhe. Experiments were acquired at 2 mg/mL in D_2_O, room temperature.

**Figure 3:**
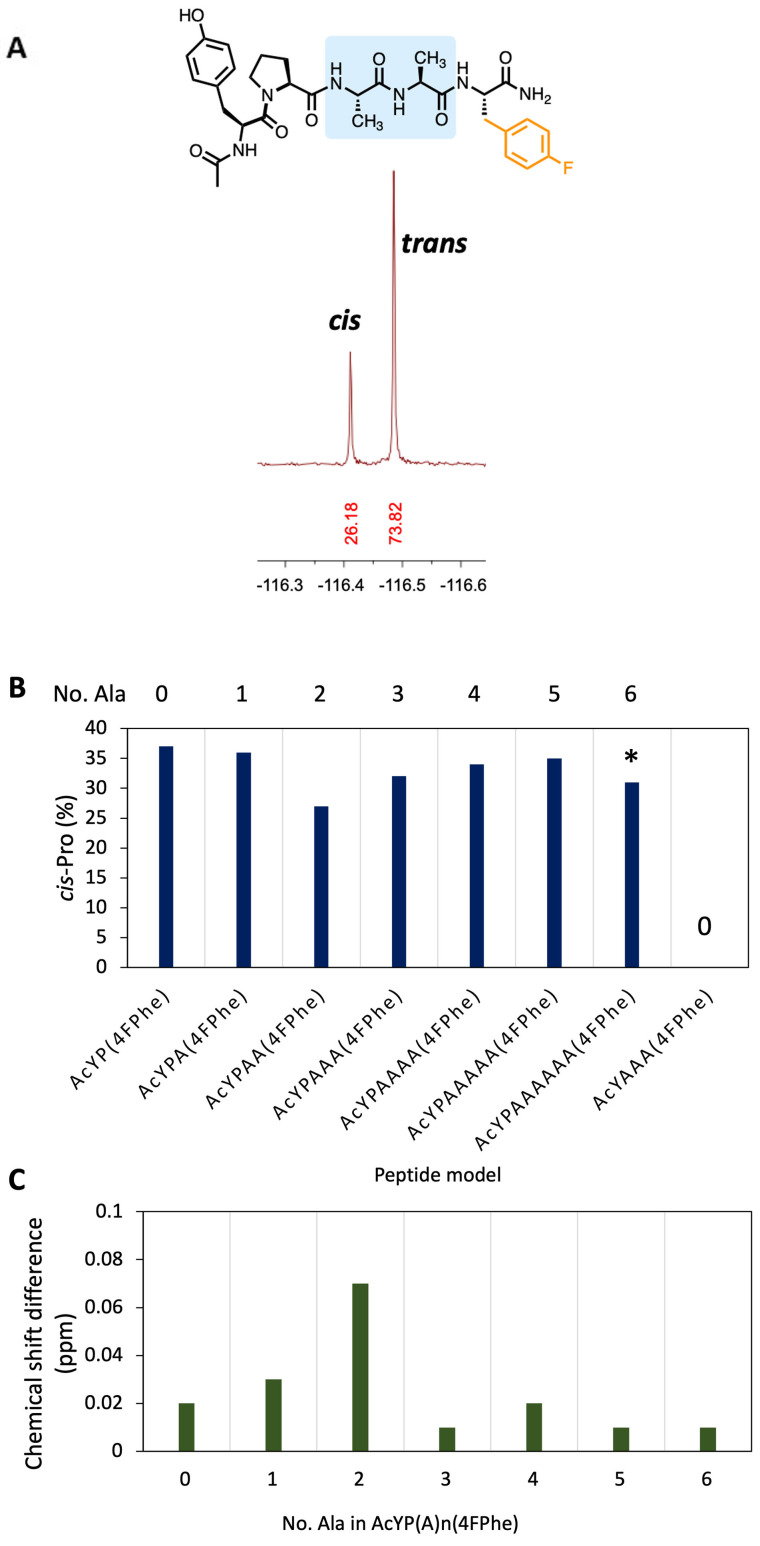
**A**) An example ^19^F NMR spectrum of peptide Ac‐Tyr‐Pro‐Ala‐Ala‐(4FPhe) (2 alanine spacer) exhibiting prolyl bond *cis‐trans* conformers. Probing the effect of proximity of the ^19^F NMR reporter (4FPhe = 4‐fluorophenylalanine) on **B**) measured *cis*‐Pro populations in model peptides using ^19^F NMR, and **C**) chemical shift difference between resonances. * Poorly resolved: not accurately quantifiable but still distinguishable ^19^F NMR spectrum.

The first point to note was the relative simplicity of the ^19^F NMR spectrum compared with the ^1^H NMR spectrum for the same model peptide (Figure 2), which significantly deconvolutes the conformational analysis. An advantage of the ^19^F NMR method is that it functions readily at physiological pH (7.4), whereas ^1^H NMR approaches often quantify *cis*‐Pro in the amide region, which disappears due to exchange above ∼ pH 7 or in D_2_O (absent in Figure 2). ^19^F NMR can also report *cis*‐Pro populations in any non‐fluorinated solvent or buffer without need for water‐suppression techniques. Peptide prolyl bond *cis*:*trans* was analysed by ^19^F NMR (relaxation delay time (D1) set at 10 s; with ^1^H‐decoupling) in (1 mg/mL in aqueous 10 mM phosphate buffered saline (10 % D_2_O), pH 7.4) at 23 °C and afforded two ^19^F resonances with the major being assigned as the thermodynamically favoured *trans* and minor as *cis* conformer (e. g., Figure 3A). Pleasingly, the control peptide containing no proline, only afforded a single resonance. The signal integrations were consistent with the reported range of *cis‐Pro* populations in related systems as mentioned earlier and were largely similar for each probe (Figure 3B), confirming that 4‐fluorophenylalanine can report on *cis*‐*trans* conformer populations over a range of amino acids (up to >6 residues away). Models containing six and seven alanine spacer residues exhibited distinct signals but were poorly resolved and not quantifiable. Slightly lower *cis*‐Pro populations were noted for cases when the 4‐fluorophenylalanine was two (27 %) or three (32 %) residues away from proline, whilst the model containing 4FPhe adjacent to Pro exhibited a slightly greater *cis*‐Pro population (37 %). In some cases, aromatic (Aro) amino acids in an Aro‐Pro‐Aro sequence can increase the propensity for a *cis* prolyl bond conformation but this effect may be less significant when the aromatic residue is electron poor, for example, 4‐fluorophenylalanine.^[13]^ Despite the Ala‐Ala spacer model appearing to slightly under‐report the %*cis* conformer (27 %), we later found that this provided a good match with the literature conformer populations (discussed subsequently).

The signal resolution between *cis*‐Pro and *trans*‐Pro resonances is an important criterion for modelling proline isomerism by NMR. By ^19^F NMR analysis the chemical shift difference for conformers varied with relative proximity of the reporter to the proline (Figure 3C; also see Supporting Information, Figure S1). Although it was possible to quantify *cis‐trans* prolyl populations up to at least 6 amino acids away from proline, the optimal signal resolution (0.07 ppm) was obtained in the model containing two alanine residues between the 4‐fluorophenylalanine and proline (AcYPAA‐4FPhe, Figure 3A, C). Therefore, this was used as the basis for our the model system to probe the effects of amino acids X and Z in X‐Pro‐Z systems.

**Figure 4:**
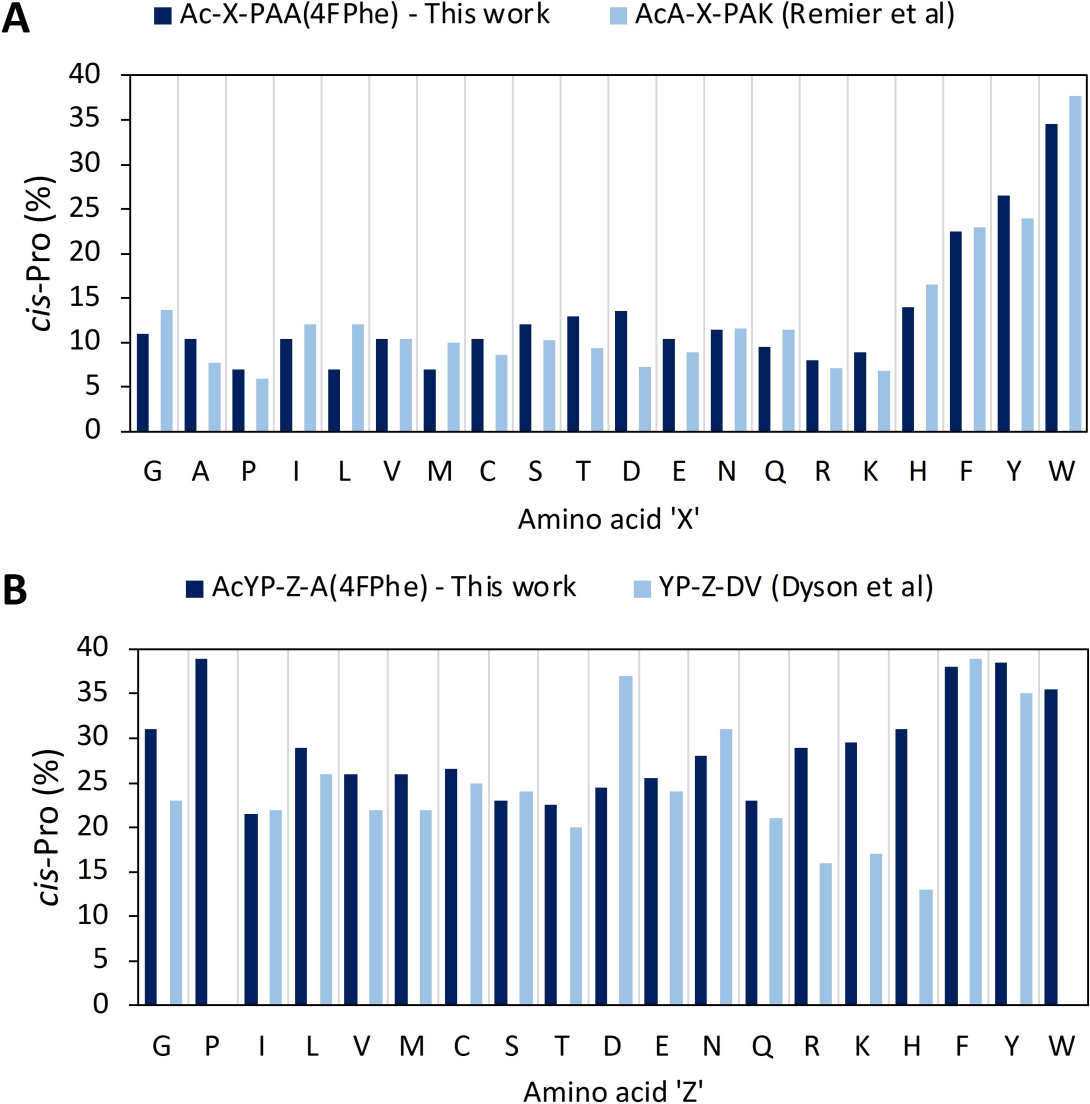
Comparison of ^19^F NMR derived *cis*‐Pro conformational preference (dark blue) with earlier published data (light blue) obtained by ^1^H NMR analysis. **A**) Comparison with model Ac‐Ala‐X‐Pro‐Ala‐Lys‐NH_2_ (Reimer et al.)^[9]^ at pH 6 where ‘X’ is at the prolyl amine. **B**) Comparison with model H_2_N‐Tyr‐Pro‐Z‐Asp‐Val‐COOH (Dyson et al.)^[10]^ at pH 4.1 where ‘Z’ is at the proline carboxamide. Note: blank columns indicate no possible comparison within the literature.

To demonstrate that this model system could be used to study the influence of amino acids proximal to proline on *cis*:*trans* ratio, 39 peptides of the form Ac‐**X**‐Pro‐Ala‐Ala‐4FPhe and Ac‐Tyr‐Pro‐**Z**‐Ala‐4FPhe were synthesised by systematically replacing the amino acid **X** or **Z** immediately adjacent to proline. As before, peptidylprolyl *cis*:*trans* conformer ratios were determined by integration of characteristic resonances obtained from ^19^F NMR (386 MHz, 23 °C, 1 mg/mL aqueous 10 mM phosphate buffered saline (10 % D_2_O), pH 7.4) analysis (Figure S2). As expected, the relative ratio of signal integration varied according to the amino acid at the proline nitrogen (Figure 4, Table S1) ‐ indicating that this was reporting the *cis*:*trans* ratio. Some pairs of signals were poorly resolved despite the model optimisation and required line‐fitting analysis using the Mnova line‐shape tool. Chemical shifts also were observed to change slightly, and indeed there was a weak correlation for higher *cis*‐Pro preferences to also exhibit greater signal resolution in ^19^F NMR spectra, perhaps due to slower rates of isomerism.

Comparison of the *cis*‐Pro populations measured by ^19^F NMR with those obtained by ^1^H NMR in related models published by Reimier et al.^[9]^ and Dyson et al.,^[10]^ respectively, afforded good agreement (Figure 4A, B and Table S2). The ^19^F NMR measurements are also consistent with the earlier reports of local conformational effects and the role of neighbouring amino acids upon proline *cis‐trans* conformational preferences, albeit in slightly different models.[^9^, ^10^] In general, the X‐Pro motif exhibits the greatest influence over the conformation of proline, particularly when X is an aromatic amino acid: Phe (22.5 %), Tyr (26.5 %) and Trp (34.5 %). This was further enhanced in Tyr‐Pro‐Aro motifs (Aro=aromatic amino acids: Phe (38 %), Tyr (38.5 %) and Trp (35.5 %)) exhibiting the highest propensity for a *cis* prolyl bond. This also supports the proposal that the Aro‐Pro‐Aro motif is a key stabiliser of type VI beta turns.[^33^, ^34^, ^35^] These observations are attributed to favourable CH‐π interactions between relatively acidic proline H_α_ or H_δ_ atoms and electron‐rich aromatic rings; and aromatic rings interacting with both faces of proline, respectively.[^9^, ^12^, ^13^, ^34^] However, in both types of model His was found to provide a lower *cis*‐Pro population than other aromatic amino acids (His‐Pro‐Ala (14 %) and Tyr‐Pro‐His (31 %), behaving more like a basic sidechain. Indeed, there was a clear trend for electron‐rich aromatics to promote higher *cis*‐Pro in the order: Trp‐Pro>Tyr‐Pro>Phe‐Pro>His‐Pro.[^9^, ^13^, ^36^] It was also clear that polar side chains, for example, Ser (12 %), Thr (13 %) Asp (13.5 %) promote a slightly higher *cis*‐Pro population in X‐Pro systems compared with Ala‐Pro (10.5 %), while Arg (8 %) or Lys (9 %) in this position leads to a subtle decrease in the *cis*‐Pro population. These results corroborate the observations from Sebak and co‐workers in their statistical analysis of *cis*‐Pro populations in intrinsically disordered proteins.^[15]^ Another interesting observation was the relatively high %*cis* for the Tyr‐Pro‐Gly (31 %) and Tyr‐Pro‐Pro (39 %) peptides, which are approximately equivalent to the Aro‐Pro‐Aro *cis*‐populations.

A small number of discrepancies were noted between the reported literature *cis*‐Pro populations and those measured using ^19^F NMR. Notably, in our hands when basic amino acids were proximal to proline in Tyr‐Pro‐Z models we observed significantly higher *cis*‐Pro populations (Z=Arg 29 %, Lys 29.5 %, His 31 %) compared with Tyr‐Pro‐Ala (26.5 %) (Figure 4B).^[37]^ Our systems appeared to exhibit higher %*cis* than the corresponding models by Dyson et al.,^[10]^ wherein the basic amino acids exhibited rather low *cis‐*Pro (vs. 16, 17 and 13 %, respectively,) compared with Tyr‐Pro‐Ala. However, our data are mostly well aligned with Reimer et al. for Ac‐X‐Pro models (X=basic amino acid), who reported *cis*‐Pro populations of X=Arg (7.2 %), Lys (6.8 %) and His (16.5 %) at pH 6 but noted that the histidine peptide Ac‐Ala‐His‐Pro‐Ala‐Lys‐NH_2_ exhibited a lower *cis‐*Pro population (9.5 %) at pH 8.

Given that the literature experiments were conducted at different pHs – often to observe non‐proline amide NHs for ^1^H NMR analysis, we were concerned that basic‐Pro or Pro‐basic motifs may be sensitive to ionisation status or intramolecular electrostatic interactions. To try to understand this better, we resynthesized exemplar peptides related to those from Dyson et al. but containing 4‐fluorophenylalanine (AcYPZD(4FPhe) where Z=ionisable residues D, H, K or R) and measured the %*cis*‐Pro at two different pHs (4.1 and 7.4) using ^19^F NMR.

**Figure 5:**
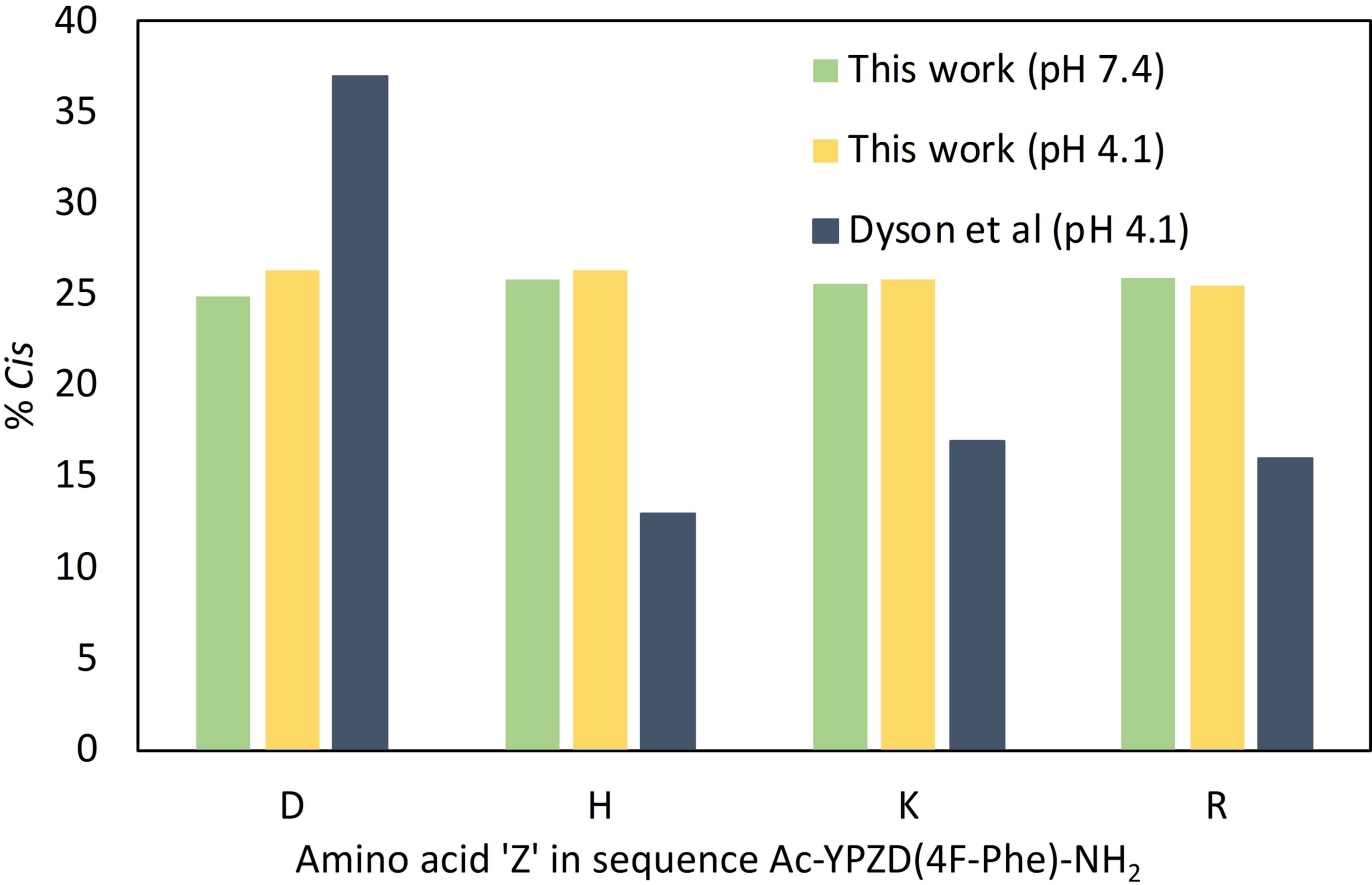
Comparison of ^19^F NMR derived *cis*‐Pro conformer populations for Dyson et al.^[10]^ models, and the resynthesized versions containing 4‐fluorophenylalanine, at pH 4.1 and 7.4.

In our analyses, the *cis‐*Pro populations were observed to align closely with our earlier models, dominated by the Tyr‐Pro motif (Figure 5), and importantly, the prolyl conformer populations were found to be largely insensitive to modest pH changes. This underlines that the use of ^19^F NMR at pH 7.4 accurately reflects what others have seen at acidic pH. This is a key advantage over ^1^H NMR methods that are difficult to evaluate at physiological pH. Therefore, the differences between this work and the Dyson work may be related to previous models containing a second adjacent ionisable group (i. e., Asp), which may provide a repulsive interaction. We also demonstrated that the %*cis* was unaffected by changes in salt (NaCl) concentration up to 1.2 M in the buffer solution (see Supporting Information, Figures S9–12).

We briefly investigated whether the effect of temperature on *cis*‐Pro population could be studied using ^19^F NMR, by heating NMR samples of peptide models Ac‐LPAA(4FPhe)‐CONH_2_ and Ac‐YPGA(4FPhe)‐CONH_2_ in pH 7.4 buffer between 10 °C–60 °C in 10 °C steps. In simple model prolines, this is shown to lead to an increase in the % *cis*‐Pro,^[23]^ whereas in small peptides, this was suggested to be a largely insignificant effect.^[38]^ In our hands, resonances relating to *cis* and *trans*‐Pro AcLPAA(4FPhe) was found to appear to coalesce towards a single signal with increasing temperature and correspondingly, the % *cis*‐Pro after line shaping analysis appeared to slightly increase above 30 °C (7 % at 10 °C to 12 % at 60 °C). Conversely, the peptide AcYPGA(4FPhe) seemed to trend towards marginally lower % *cis*‐Pro (26 % at 10 °C to 22 % at 60 °C) (see Supporting Information, Figure S7, Table S3). Albeit, in both cases, this was a minor change, this further emphasises that ^19^F NMR measurements can perhaps provide additional insight into subtle effects that are challenging to observe by ^1^H NMR.

**Figure 6:**
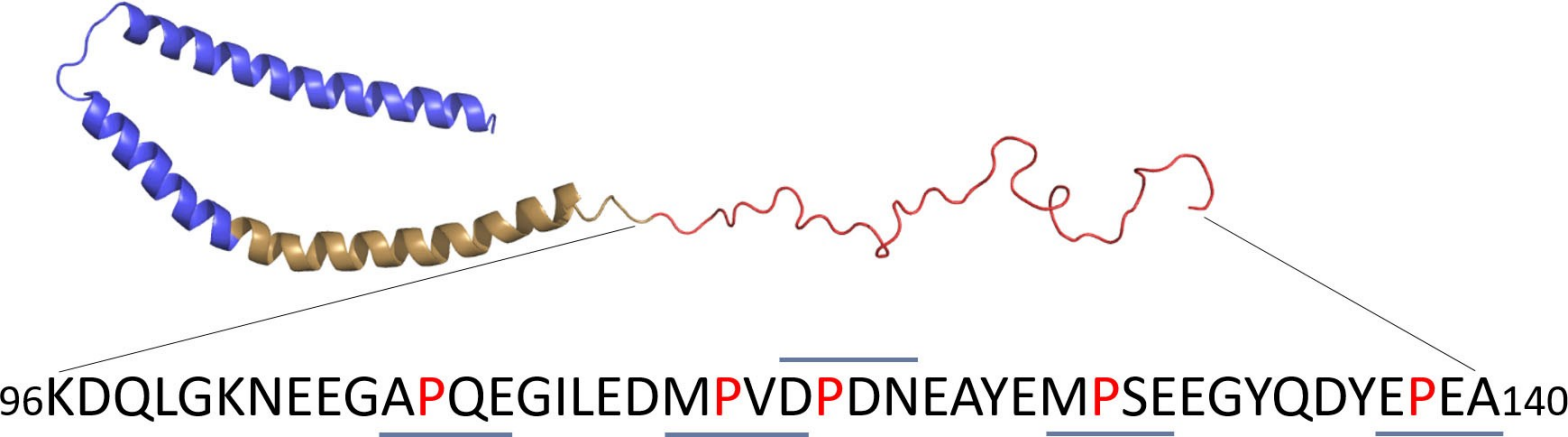
The intrinsically disordered C‐terminal region of α‐synuclein (PDB: 1XQ8), which consists of amino acids 96 to 140 (proline residues in red). Tetramer sequences, indicated with bold lines, informed the design of the 5 pentapeptide models used in this study.

α‐Synuclein is a generally disordered protein, but can assume stable misfolded protein oligomers and aggregates, which is a hallmark of Parkinson's disease pathophysiology. To demonstrate that the observed prolyl bond populations are relatively conserved in different peptides and are governed mainly by local sequences, we prepared a series of pentapeptide models of the proline‐rich (5 proline residues in full length protein) intrinsically disordered C‐terminal region of α‐synuclein (Figure 6, Table 1). These models contained disorder‐promoting amino acids for example Q, E, D, N rather than alanines between the proline and ^19^F reporter. It was clear from these experiments, that the %*cis‐*Pro in each model was similar to that observed in our earlier models and the observations of Sebak et al. in intrinsically‐disordered proteins. This indicates that prolyl bond conformation is controlled predominantly by the proline *N*‐acyl substituent in these models and that %*cis‐*Pro may be generally predictable within an unstructured polypeptide. Importantly, this also highlights that the ^19^F NMR approaches are broadly useful and sensitive to small changes in conformer populations.

**Table 1:** Pentapeptide models of the proline‐rich intrinsically disordered C‐terminal region of α‐synuclein containing 4‐fluorophenylalanine. % *cis‐*Pro measured by ^19^F NMR at pH 7.4.

Entry	Sequence	%*cis‐*Pro, pH 7.4
1	Ac‐APQE(4FPhe)‐NH_2_	8
2	Ac‐MPVD(4FPhe)‐NH_2_	12
3	Ac‐DPDN(4FPhe)‐NH_2_	10
4	Ac‐MPSE(4FPhe)‐NH_2_	6
5	Ac‐EPEA(4FPhe)‐NH_2_	11

## Conclusion

In summary, the prolyl bond conformational trends reported herein using ^19^F NMR are broadly similar to the previous observations using different techniques, and importantly, align with the general trends for prolyl bond conformation in folded protein structures – indicating that prolyl bond conformation is predominantly governed by local effects. Furthermore, very similar prolyl bond conformational populations were observed to occur in short models of the intrinsically disordered C‐terminal region of α‐synuclein, indicating that these prolyl bond populations are conserved in flexible peptides and proteins.

This work has demonstrated that incorporating fluorinated reporter groups via 4‐fluorophenylalanine is a reliable way to quantify prolyl bond status in peptide models. If positioned carefully, it does not influence the prolyl bond status and advantageously, does not need to be adjacent or attached to the proline to provide quantifiable and distinguishable signals, providing flexibility in model design. Furthermore, Fmoc‐L‐4‐fluorophenylalanine is commercially available at low cost, which makes this approach useful for a wide range of researchers. The simplicity of the ^19^F NMR read‐out compared to the corresponding ^1^H NMR spectrum and the ability to quantify *cis*‐Pro populations at a range of temperatures, physiological pH and in a variety of solvents and buffers without solvent‐suppression and in complex mixtures such as cell lysate, will be of broad and important use in biological chemistry. Specifically, this will be utilised in our future studies as a straightforward tool to understand the consequences of point mutations or the role of specific residues upon *cis‐trans* isomerism at specific proline residues in larger, more complex and intrinsically disordered and proline‐rich systems.

## Experimental Section

### Materials

All standard Fmoc‐L‐amino acids, Oxyma and Rink amide ProTide resin were purchased from CEM Microwave Technologies Ltd, UK. Diisopropylcarbodiimide (DIC), Fmoc‐L‐4‐fluorophenylalanine (Fmoc‐4F‐Phe) and trifluoroacetic acid (TFA) were obtained from Fluorochem, UK. Triisopropylsilane (TIPS), piperidine and acetic anhydride were purchased from Sigma Aldrich, UK. Solvents, including diethyl ether (Et_2_O), peptide synthesis grade dimethylformamide (DMF) and HPLC grade solvents: acetonitrile (MeCN) and methanol (MeOH) were purchased from Fisher Scientific, UK. NMR solvents (e. g. D_2_O) were purchased from Fluorochem, UK. All chemicals were of the highest analytical grade and were used without further purification.

### Synthesis of model peptides

Fmoc solid‐phase peptide synthesis was performed using automated Fmoc‐SPPS on a Liberty Blue microwave‐assisted peptide synthesiser (CEM). SPPS was conducted using Rink amide ProTide resin (0.56 mmol/g, 0.1 mmol, 179 mg). Fmoc protected amino acids were used in 5 equivalents (0.2 M in DMF) with DIC (1 M stock solution in DMF; 10 equiv.) and Oxyma Pure (1 M stock solution, 5 equiv) as activators. Standard coupling procedures employed single coupling of each amino acid (2.5 min, 90 °C). Amino acids bearing thermally‐sensitive protecting groups, for example, Fmoc‐L‐His(Trt)‐OH and Fmoc‐L‐Cys(Trt)‐OH, were coupled under milder conditions (50 °C for 10 min). Fmoc deprotection was performed by treatment of the resin with piperidine (20 % *v/v* in DMF, 4 mL). Following the removal of the final Fmoc‐protecting group, peptides were acetylated (where relevant) using acetic anhydride (20 % in DMF) at 37 °C for 15 min and repeated once, followed by washing the resin with DMF three times and drying under vacuum. The resin was washed several times with Et_2_O (5 mL) and cleaved from the resin using a cocktail of TFA/TIPS/H_2_O (95/2.5/2.5) for 2 h at 37 °C or 4 h at RT in a disposable fritted syringe. Afterwards, the suspension was filtered and precipitated in ice‐cold Et_2_O. The resulting suspension was centrifuged (5 min at 5000 rpm) and the pellet was resuspended with Et_2_O (10 mL). This was centrifuged again (5 min 5000 rpm) and the Et_2_O was discarded. Crude peptide mixtures were purified using RP‐HPLC (Agilent 1260), equipped with a Waters Xbridge peptide BEH C18 Prep column (19×100 mm, 5 μm, 130 Å) using a linear gradient from 5–95 % MeOH in water containing 0.1 % TFA over 20 minutes at a flow rate of 8 mL/min.

### Characterisation of model peptides

*High‐resolution mass spectrometry method A*: performed using an Agilent 6530 accurate mass QToF system. Separation was performed on an Agilent ZORBAX Eclipse Plus C18 Rapid Resolution HD analytical C18 column (1.8 μm particle size, 2.1×50 mm) at 25 °C. The mobile phase of the system consisted of water (with 0.1 % TFA, eluent A) and Methanol (with 0.1 % TFA, eluent B). the 12.5 min gradient was programmed as follows: 0–1 min 95 % A, 1.0–7.0 min to 0 % A, 7.0–9.0 min hold 0 %, 9.0–9.5 min to 95 % A, 9.5‐12.5 min hold 95 % A. The injection volume was set to 2 μL and the flow rate to 0.5 mL/min, respectively. Monitoring was performed at λ=215 nm. Mass spectral analysis of peptides was performed in the positive ionisation (ESI+) mode in full scan mode (*m/z* 100–3200). Operating pressures were in the range of 2000–3000 PSI. Electrospray ionisation mass spectrometry was conducted in positive ion mode (*m/z* range: 50–3200) using a fragmentor voltage of 150 V, gas temperature of 325 °C (flow 10 L/min) and sheath gas temperature of 400 °C (flow 11 L/min). Exact mass measurements of the products were based on the protonated ions [M+H]^+^ and were detected as sodiated adducts [M+Na]^+^.

*Analytical HPLC method B (retention times* ^b^ *)*: Separation was performed on a Waters XBridge C18 analytical column (3.5 μm particle size, 4.6×50 mm) at 40 °C. The mobile phase of the system consisted of water (with 0.1 % TFA, eluent A) and Methanol (with 0.1 % TFA, eluent B). The 15 min gradient was programmed as follows: 0–10 min 95 % A to 20 % A, 10–11 min to 5 % A, 11–12 min hold 5 % A, 12–13 min to 95 % A, 13–15 min hold 95 % A. The injection volume was set to 2 μL and the flow rate to 1 mL/min, respectively. Monitoring was performed at λ=215 nm.

### ^19^F NMR analysis of amide bond conformation

*General procedure*: Purified peptides (1 mg/mL) were dissolved in 1x PBS solution (pH 7.4, 10 mM by phosphate, 10 % D_2_O). The NMR experiments were conducted using a Bruker 400 MHz NMR spectrometer. The experiments employed were 1D ^19^F NMR: 386 MHz, proton decoupled, 128 scans, D1=10 sec, 23°C. The NMR data were processed using TopSpin (v4.0.5) or MNova (v14.2.1‐27684).

*pH Experiment Procedure*: Purified peptides (2 mg/mL) were dissolved in 10 % D_2_O/H_2_O. The pH was adjusted to 4.1 with HCl, in accordance with the Dyson method,^1,2^ or a combination of HCl and NaOH for pH 7.4. The NMR experiments were the same as the general procedure other than being completed in triplicate.

*Variable Temperature Experiment Procedure*: Purified peptides (2 mg/mL) were dissolved in 10 % D_2_O/H_2_O. The NMR acquisition parameters were the same as the general procedure other than being conducted over a range of temperatures from 10 °C to 60 °C over 10 °C steps.

## Conflict of interest

The authors declare no conflict of interest.

1

## Supporting information

As a service to our authors and readers, this journal provides supporting information supplied by the authors. Such materials are peer reviewed and may be re‐organized for online delivery, but are not copy‐edited or typeset. Technical support issues arising from supporting information (other than missing files) should be addressed to the authors.

Supporting Information

## Data Availability

The data that support the findings of this study are available in the supplementary material of this article.
