# Supplementary material for: Probing Peptidylprolyl Bond cis/trans Status Using Distal 19F NMR Reporters
Source: Chemistry. 2023 Feb 16;29(16):e202203017. doi: 10.1002/chem.202203017 (PMC10946801; doi:10.1002/chem.202203017)
Supplement: Supplementary file 1 — Supporting Information [file CHEM-29-0-s001.pdf]

Chemistry–A European Journal

Supporting Information

Probing Peptidylprolyl Bond *cis*/*trans* Status Using Distal ^{19}F NMR Reporters

Patrick M. Killoran, George S. M. Hanson, Sanne J. M. Verhoork, Madeleine Smith, Davide Del Gobbo, Lu-Yun Lian, and Christopher R. Coxon*

Probing peptidylprolyl bond *cis/trans* status using distal ^{19}F NMR reporters

Patrick M. Killoran,[a‡] George S.M. Hanson,[c‡] Sanne J. M. Verhoork,[a] Madeleine Smith,[a] Davide Del Gobbo,[a] Lu-Yun Lian,[b] Christopher R. Coxon[c*]

[a] School of Pharmacy and Biomolecular Sciences, Faculty of Science, Liverpool John Moores University, Liverpool, Merseyside, L3 3AF, UK

[b] Institute of Integrative Biology, Biosciences Building, The University of Liverpool, Crown Street, Liverpool, L69 7ZB, UK

[c] School of Chemistry, The University of Edinburgh, Joseph Black Building, David Brewster Road, Edinburgh, EH14 4AS, UK

‡Authors contributed equally and can both list as first author.

*Corresponding author. Email: chris.coxon@ed.ac.uk

Supplementary information

1. **Characterisation and analysis**

   1.1 Probing the effect of proximity of the ^{19}F NMR reporter to the prolyl-bond……………………………………………………………………………..2

   1.2 Probing the effect of amino acids proximal to the prolyl-bond……………….3

   1.3 Probing the effect of pH……………………………………………………..13

   1.4 Probing the effect of temperature on cis-Pro……………………………...14

   1.5 α-Synuclein C-terminal region model pentapeptides………………………..15

   1.6 Probing the effect of NaCl concentration (0 to 1.2 M NaCl)………………...16

   1.7 Probing the effect of DMSO on %cis……………………………..…………21

2. ***Appendix***

   2.1 Analytical data for AcYP(A)n(4FPhe) model peptides…..…………………22

   2.2 Analytical data for X-Pro-Z model peptides ……………………….………26

1. Characterisation and analysis

1.1 Probing the effect of proximity of the ^{19}F NMR reporter to the prolyl-bond

What is the effect of reporter proximity to Pro?

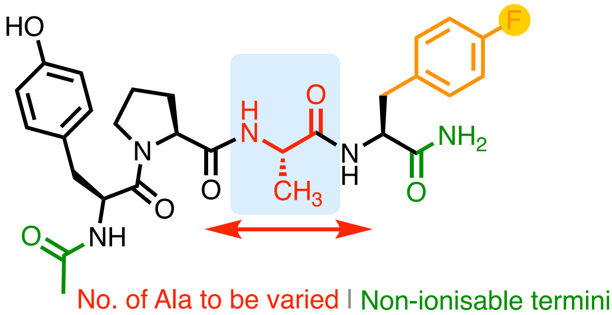

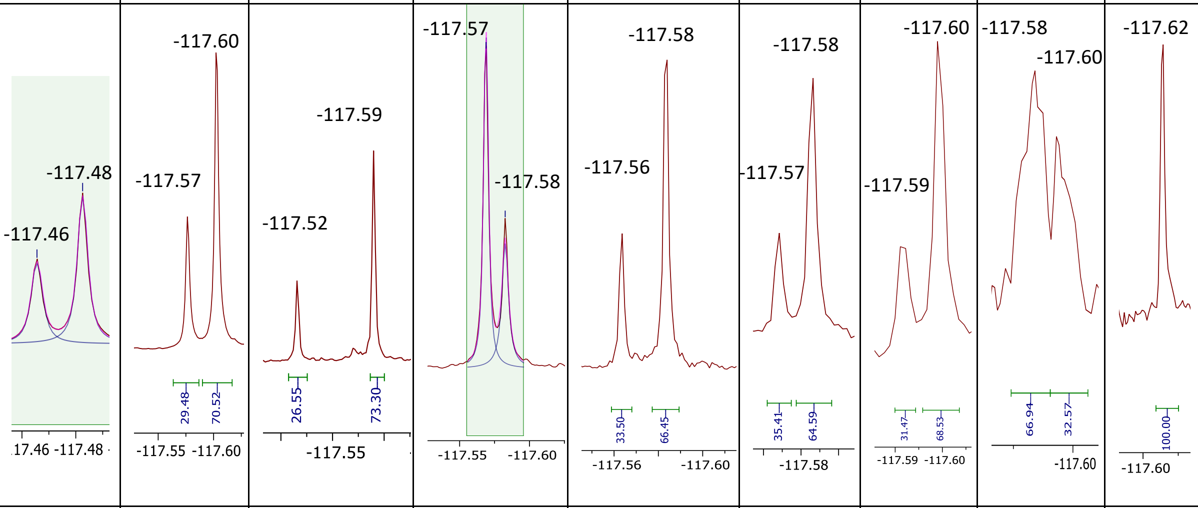

Number of alanine residues								
0	1	2	3	4	5	6	7	0
-117.48<br>-117.46<br>17.46 -117.48	-117.60<br>-117.57<br>29.48 70.52<br>-117.55 -117.60	-117.59<br>-117.52<br>26.55 73.30<br>-117.55	-117.57<br>-117.58<br>-117.55 -117.60	-117.58<br>-117.56<br>33.50 66.45<br>-117.56 -117.60	-117.58<br>-117.57<br>35.41 64.59<br>-117.58	-117.60<br>-117.59<br>31.47 68.53<br>-117.59 -117.60	-117.58<br>-117.60<br>66.94 32.57<br>-117.60	-117.62<br>100.00<br>-117.60
AcYP(4FPh e)	AcYPA-(4FPhe)	AcYPAA-(4FPhe)	AcYPAAA-(4FPhe)	AcYPAAAA-(4FPhe)	AcYPAAA-AA(4FPhe)	AcYPAAA-AAA-(4FPhe)	AcYPAAA-AAAA-(4FPhe)<br>**Note:** poorly soluble	YAAA-(4FPhe)

Figure S1. ^{19}F NMR spectra for the 9 model peptides designed to probe the effect of proximity of the reporter top the proline residue. Line fitting (shaded green) has been applied (Mnova) where baseline resolution is absent. Chemical shifts are in ppm and resonances are referenced to TFA (-76.55 ppm)

1.2 Probing the effect of amino acids proximal to the prolyl-bond

What is the effect of X & Z on *trans*:*cis*?

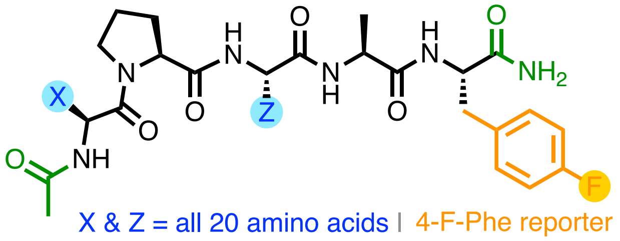

Figure S2. ^{19}F NMR analysis of *cis-trans*-Pro populations. Chemical shifts in ppm.

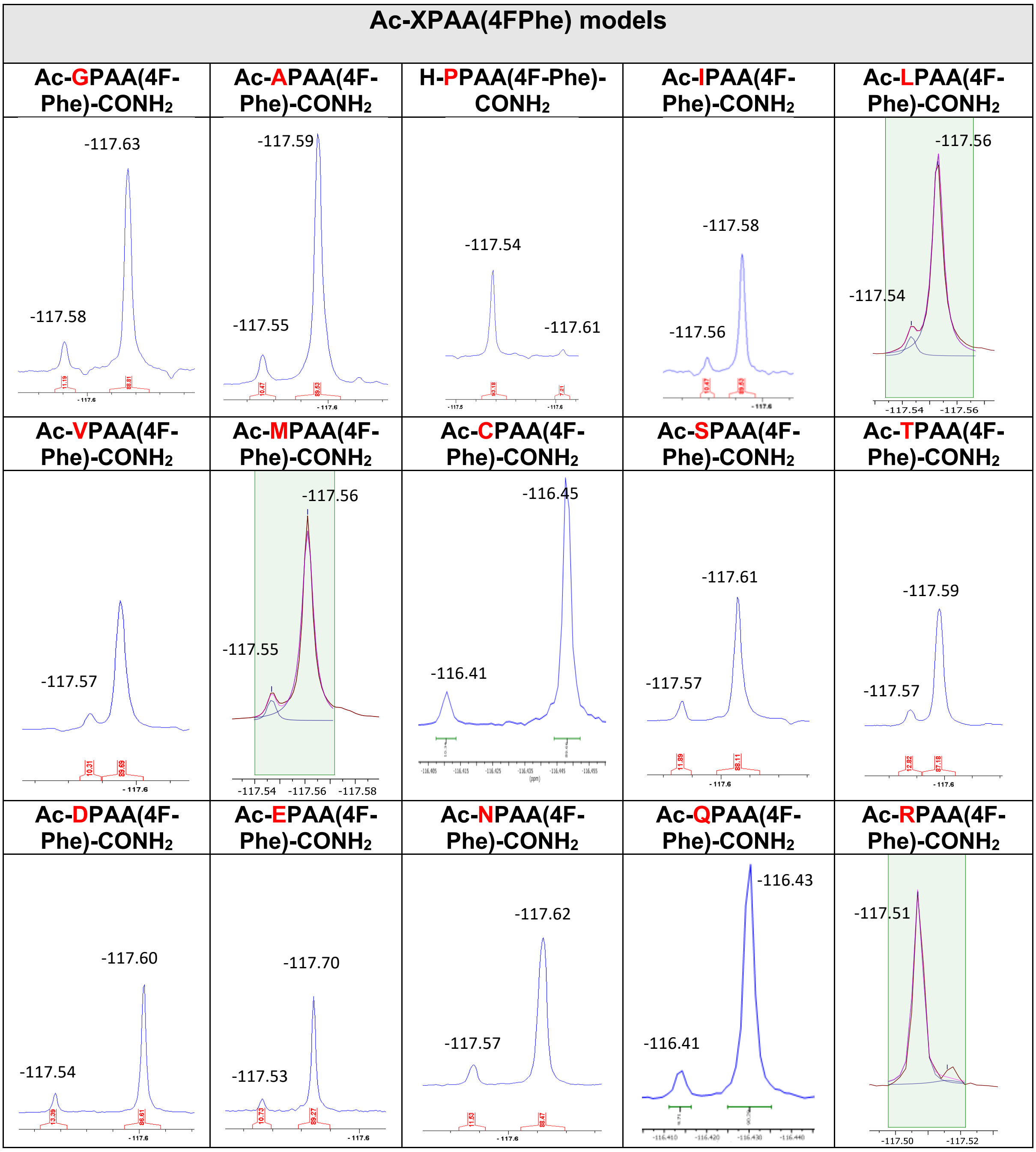

Ac-KPAA(4F-Phe)-$CONH_2$	Ac-HPAA(4F-Phe)-$CONH_2$	Ac-FPAA(4F-Phe)-$CONH_2$	Ac-YPAA(4F-Phe)-$CONH_2$	Ac-WPAA(4F-Phe)-$CONH_2$
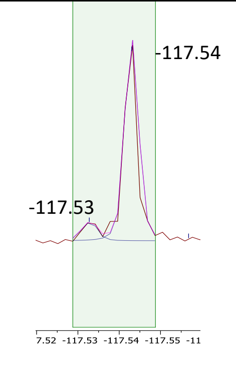	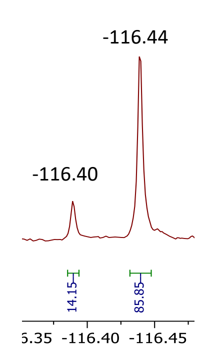	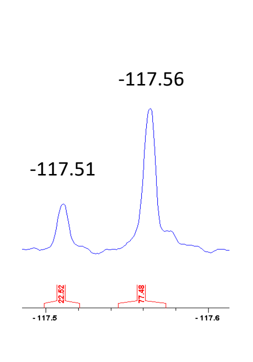	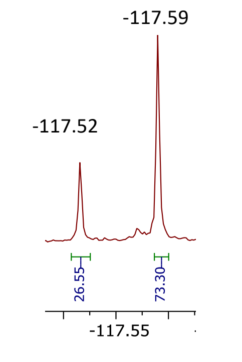	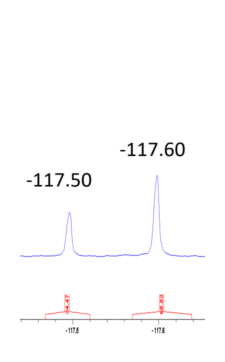

Ac-YPZA(4FPhe) models

Ac-YPGA(4F-Phe)-$CONH_2$	Ac-YPPA(4F-Phe)-$CONH_2$	Ac-YPIA(4F-Phe)-$CONH_2$	Ac-YPLA(4F-Phe)-$CONH_2$	Ac-YPVA(4F-Phe)-$CONH_2$
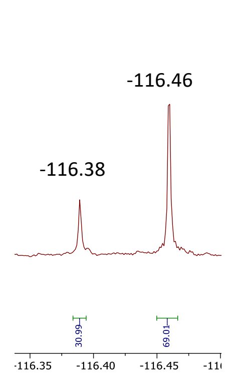	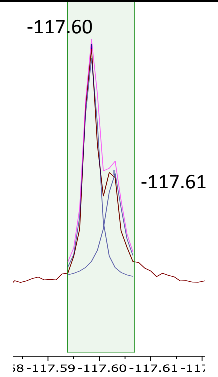	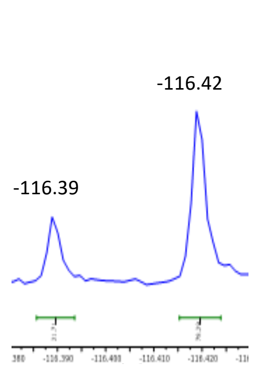	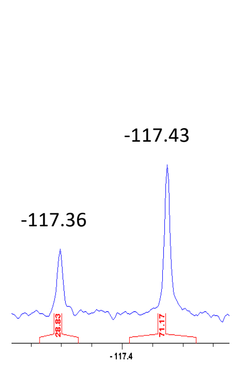	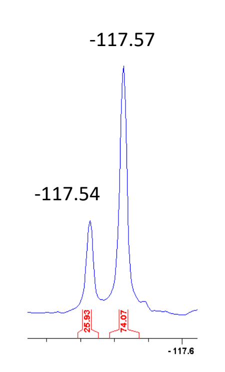
**Ac-YPMA(4F-Phe)-$CONH_2$**	**Ac-YPCA(4F-Phe)-$CONH_2$**	**Ac-YPSA(4F-Phe)-$CONH_2$**	**Ac-YPTA(4F-Phe)-$CONH_2$**	**Ac-YPDA(4F-Phe)-$CONH_2$**
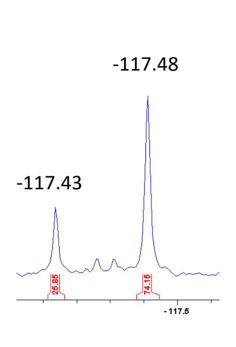	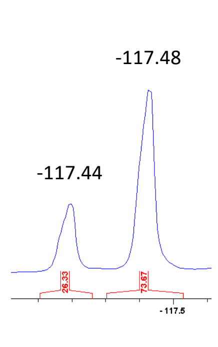	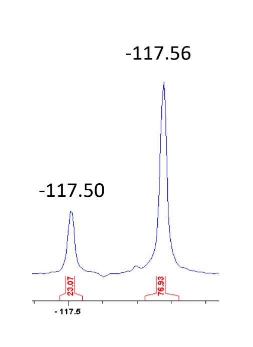	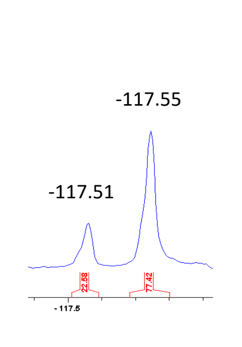	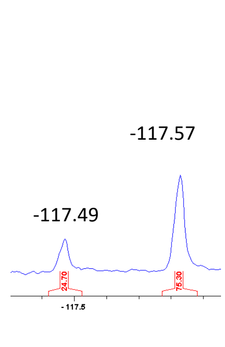
**Ac-YPEA(4F-Phe)-$CONH_2$**	**Ac-YPNA(4F-Phe)-$CONH_2$**	**Ac-YPQA(4F-Phe)-$CONH_2$**	**Ac-YPRA(4F-Phe)-$CONH_2$**	**Ac-YPKA(4F-Phe)-$CONH_2$**
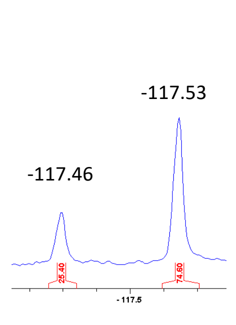	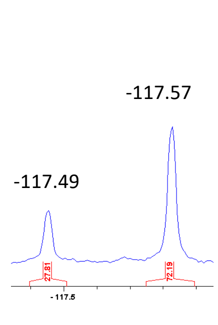	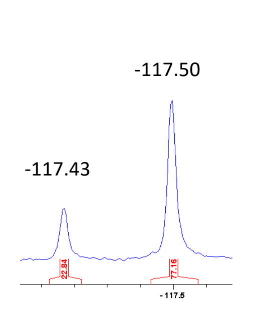	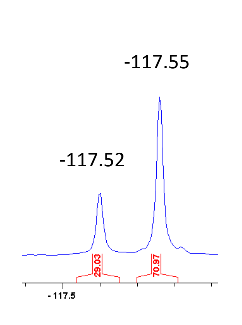	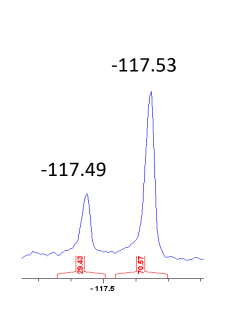

Ac-YPHA(4F-Phe)-$CONH_2$	Ac-YPFA(4F-Phe)-$CONH_2$	Ac-YPYA(4F-Phe)-$CONH_2$	Ac-YPWA(4F-Phe)-$CONH_2$

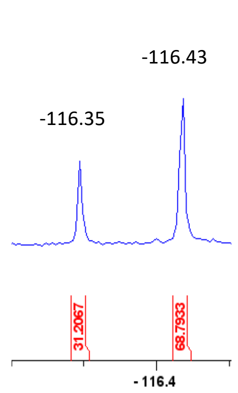

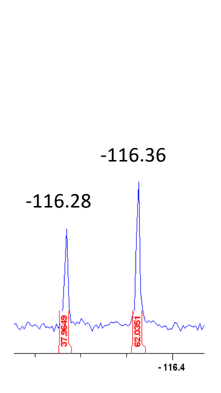

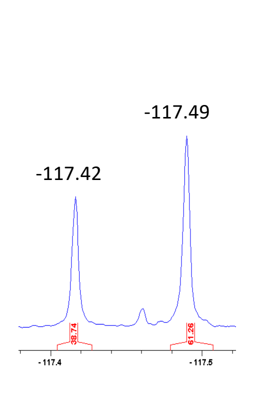

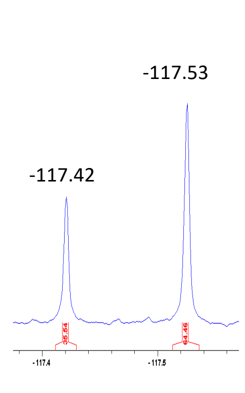

Note: Line shape analysis (green shading) has been performed (Mnova) in cases where baseline resolution was absent. Resonances referenced to TFA (-76.55 ppm)

Table S1. The effect of replacing amino acids flanking proline residues on measured *trans:cis* ratio in model peptides. Modified amino acid in each model indicated in red. 4FPhe = 4-fluorophenylalanine reporter group. All ^{19}F NMR spectra were acquired at 386 MHz at 300 K with a range of scans (16 - 256) as required. Performed in 10 mM, pH 7.4 phosphate buffered saline (includes NaCl (137 mM) & KCl (2.7 mM)) at 1 mg/mL peptide concentration.

**Entry**			**Sequence**				***cis*-Pro (%)**	***trans*-Pro (%)**	**$K_{trans/cis}$**	**Type**
1	Ac	G	P	A	A	4FPhe	11	89	8.1	Small
2	Ac	A	P	A	A	4FPhe	10.5	89.5	8.5	Small
3	Ac	P	P	A	A	4FPhe	7	93	13.3	Small
4	Ac	I	P	A	A	4FPhe	10.5	89.5	8.5	Hydrophobic
5	Ac	L	P	A	A	4FPhe	7	93	13.3	Hydrophobic
6	Ac	V	P	A	A	4FPhe	10.5	89.5	8.5	Hydrophobic
7	Ac	M	P	A	A	4FPhe	7	93	13.3	Hydrophobic
8	Ac	C	P	A	A	4FPhe	10.5	89.5	8.5	Nucleophilic
9	Ac	S	P	A	A	4FPhe	12	88	7.3	Nucleophilic
10	Ac	T	P	A	A	4FPhe	13	87	6.7	Nucleophilic
11	Ac	D	P	A	A	4FPhe	13.5	86.5	6.4	Acidic
12	Ac	E	P	A	A	4FPhe	10.5	89.5	8.5	Acidic
13	Ac	N	P	A	A	4FPhe	11.5	88.5	7.7	Amide
14	Ac	Q	P	A	A	4FPhe	9.5	90.5	9.5	Amide
15	Ac	R	P	A	A	4FPhe	8	92	11.5	Basic
16	Ac	K	P	A	A	4FPhe	9	91	10.1	Basic
17	Ac	H	P	A	A	4FPhe	14	86	6.1	Basic/Aromatic
18	Ac	F	P	A	A	4FPhe	22.5	77.5	3.4	Aromatic
19	Ac	Y	P	A	A	4FPhe	26.5	73.5	2.8	Aromatic
20	Ac	W	P	A	A	4FPhe	34.5	65.5	1.9	Aromatic
21	Ac	Y	P	G	A	4FPhe	31	69	2.2	Small
22	Ac	Y	P	P	A	4FPhe	39	61	1.6	Small
23	Ac	Y	P	I	A	4FPhe	21.5	78.5	3.7	Hydrophobic
24	Ac	Y	P	L	A	4FPhe	29	71	2.4	Hydrophobic
25	Ac	Y	P	V	A	4FPhe	26	74	2.8	Hydrophobic
26	Ac	Y	P	M	A	4FPhe	26	74	2.8	Hydrophobic
27	Ac	Y	P	C	A	4FPhe	26.5	73.5	2.8	Nucleophilic

28	Ac	Y	P	S	A	4FPhe	23	77	3.3	Nucleophilic
29	Ac	Y	P	T	A	4FPhe	22.5	77.5	3.4	Nucleophilic
30	Ac	Y	P	D	A	4FPhe	24.5	75.5	3.1	Acidic
31	Ac	Y	P	E	A	4FPhe	25.5	74.5	2.9	Acidic
32	Ac	Y	P	N	A	4FPhe	28	72	2.6	Amide
33	Ac	Y	P	Q	A	4FPhe	23	77	3.3	Amide
34	Ac	Y	P	R	A	4FPhe	29	71	2.4	Basic
35	Ac	Y	P	K	A	4FPhe	29.5	70.5	2.4	Basic
36	Ac	Y	P	H	A	4FPhe	31	69	2.2	Basic/Aromatic
37	Ac	Y	P	F	A	4FPhe	38	62	1.6	Aromatic
38	Ac	Y	P	Y	A	4FPhe	38.5	61.5	1.6	Aromatic
39	Ac	Y	P	W	A	4FPhe	35.5	64.5	1.8	Aromatic

Table S2. Summary of analytical data for peptides **1** - **39**.

**Peptide 1**	**Ac-GPAA(4FPhe)-CONH$_2$**
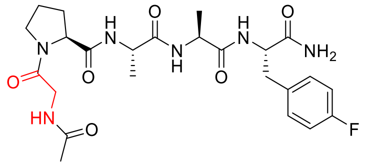	HPLC RT: 5.84 min<br>HPLC-MS (ESI+) Calc. $C_{24}H_{33}FN_6O_6$: 520.5538.<br>Found: 521.2518 $[M+H]^+$<br>^{19}F NMR: δ (ppm) -117.58, -117.63
**Peptide 2**	**Ac-APAA(4FPhe)-CONH$_2$**
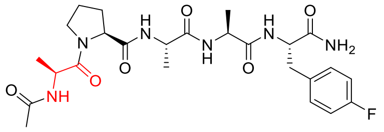	HPLC RT: 5.95 min<br>HPLC-MS (ESI+) Calc. $C_{25}H_{35}FN_6O_6$: 534.5804.<br>Found: 535.2674 $[M+H]^+$<br>^{19}F NMR: δ (ppm) -117.55, -117.59
**Peptide 3**	**H-PPAA(4FPhe)-CONH$_2$**
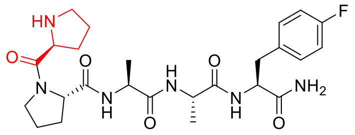	HPLC RT: 6.35 min[b]<br>HPLC-MS (ESI+) Calc. $C_{27}H_{37}FN_6O_6$: 560.6177.<br>Found: 561.2837 $[M+H]^+$<br>^{19}F NMR: δ (ppm) -117.54, -117.61
**Peptide 4**	**Ac-IPAA(4FPhe)-CONH$_2$**
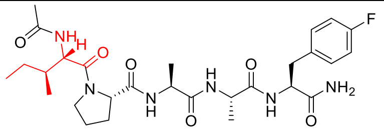	HPLC RT: 6.78 min<br>HPLC-MS (ESI+) Calc. $C_{28}H_{41}FN_6O_6$: 576.6601.<br>Found: 577.3143 $[M+H]^+$<br>^{19}F NMR: δ (ppm) -117.56, -117.58
**Peptide 5**	**Ac-LPAA(4FPhe)-CONH$_2$**
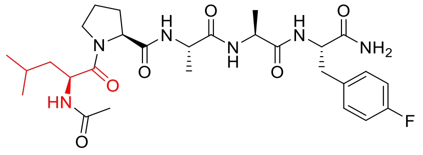	HPLC RT: 6.85 min<br>HPLC-MS (ESI+) Calc. $C_{28}H_{41}FN_6O_6$: 576.6601.<br>Found: 577.3141 $[M+H]^+$<br>^{19}F NMR: δ (ppm) -117.54, -117.56
**Peptide 6**	**Ac-VPAA(4FPhe)-CONH$_2$**
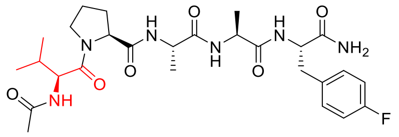	HPLC RT: 6.42 min<br>HPLC-MS (ESI+) Calc. $C_{27}H_{39}FN_6O_6$: 562.6335.<br>Found: 563.2986 $[M+H]^+$<br>^{19}F NMR: δ (ppm) -117.57 -117.59
**Peptide 7**	**Ac-MPAA(4FPhe)-CONH$_2$**
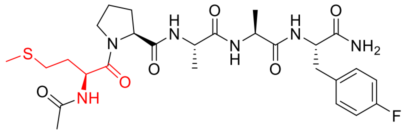	HPLC RT: 6.48 min<br>HPLC-MS (ESI+) Calc. $C_{27}H_{39}FN_6O_6S$: 594.6986.<br>Found: 595.2704 $[M+H]^+$<br>^{19}F NMR: δ (ppm) -117.55, -117.56
**Peptide 8**	**Ac-CPAA(4FPhe)-CONH$_2$**
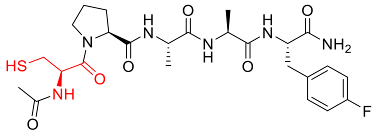	HPLC RT: 6.52 min[b]<br>HPLC-MS (ESI+) Calc. $C_{25}H_{35}FN_6O_6S$: 566.6454.<br>Found: 567.2390 $[M+H]^+$<br>^{19}F NMR: δ (ppm) -116.41, -116.45

**Peptide 9**	**Ac-SPAA(4FPhe)-CONH$_2$**
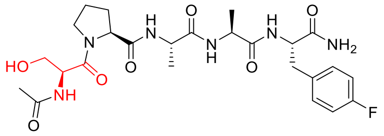	HPLC RT: 5.75 min<br>HPLC-MS (ESI+) Calc. $C_{25}H_{35}FN_6O_7$: 550.5798.<br>Found: 551.2621 $[M+H]^+$<br>^{19}F NMR: δ (ppm) -117.57, -117.61
**Peptide 10**	**Ac-TPAA(4FPhe)-CONH$_2$**
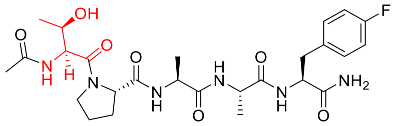	HPLC RT: 5.81 min<br>HPLC-MS (ESI+) Calc. $C_{26}H_{37}FN_6O_7$: 564.6064.<br>Found: 565.2773 $[M+H]^+$<br>^{19}F NMR: δ (ppm) -117.57, -117.59
**Peptide 11**	**Ac-DPAA(4FPhe)-CONH$_2$**
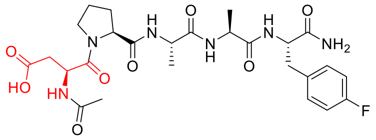	HPLC RT: 6.06 min<br>HPLC-MS (ESI+) Calc. $C_{26}H_{35}FN_6O_8$: 578.5899.<br>Found: 579.2575 $[M+H]^+$<br>^{19}F NMR: δ (ppm) -117.54, -117.60
**Peptide 12**	**Ac-EPAA(4FPhe)-CONH$_2$**
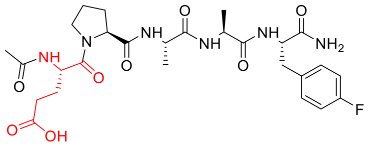	HPLC RT: 4.22 min<br>HPLC-MS (ESI+) Calc. $C_{27}H_{37}FN_6O_8$: 592.6165.<br>Found: 593.2735 $[M+H]^+$<br>^{19}F NMR: δ (ppm) -117.53, -117.70
**Peptide 13**	**Ac-NPAA(4FPhe)-CONH$_2$**
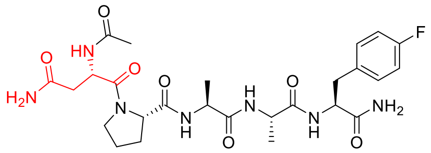	HPLC RT: 5.78 min<br>HPLC-MS (ESI+) Calc. $C_{26}H_{36}FN_7O_7$: 577.6051.<br>Found: 578.2731 $[M+H]^+$<br>^{19}F NMR: δ (ppm) -117.57, -117.62
**Peptide 14**	**Ac-QPAA(4FPhe)-CONH$_2$**
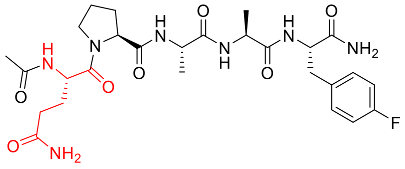	HPLC RT: 5.55 min<br>HPLC-MS (ESI+) Calc. $C_{27}H_{38}FN_7O_7$: 591.6317.<br>Found: 592.2891 $[M+H]^+$<br>^{19}F NMR: δ (ppm) -116.41, -116.43
**Peptide 15**	**Ac-RPAA(4FPhe)-CONH$_2$**
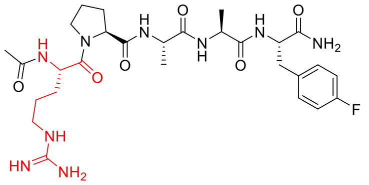	HPLC RT: 5.23 min<br>HPLC-MS (ESI+) Calc. $C_{28}H_{42}FN_9O_6$: 619.6882.<br>Found: 620.3307 $[M+H]^+$<br>^{19}F NMR: δ (ppm) -117.51, -117.52
**Peptide 16**	**Ac-KPAA(4FPhe)-CONH$_2$**
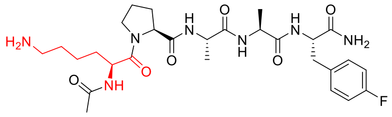	HPLC RT: 4.83 min<br>HPLC-MS (ESI+) Calc. $C_{28}H_{42}FN_7O_6$: 591.6748.<br>Found: 592.3251 $[M+H]^+$<br>^{19}F NMR: δ (ppm) -117.53, -117.54

**Peptide 17**	**Ac-HPAA(4FPhe)-CONH$_2$**
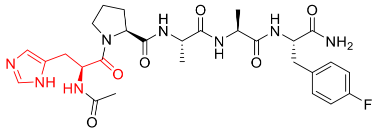	HPLC RT: 5.19 min[b]<br>HPLC-MS (ESI+) Calc. $C_{28}H_{37}FN_8O_6$: 600.6418.<br>Found: 601.2895 $[M+H]^+$<br>^{19}F NMR: δ (ppm) -116.40, -116.44
**Peptide 18**	**Ac-FPAA(4FPhe)-CONH$_2$**
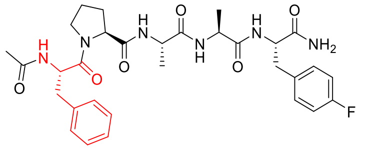	HPLC RT: 5.43 min<br>HPLC-MS (ESI+) Calc. $C_{31}H_{39}FN_6O_6$: 610.6764.<br>Found: 611.2990 $[M+H]^+$<br>^{19}F NMR: δ (ppm) -117.51, -117.56
**Peptide 19**	**Ac-YPAA(4FPhe)-CONH$_2$**
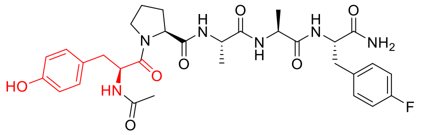	HPLC RT: 4.95 min<br>HPLC-MS (ESI+) Calc. $C_{31}H_{39}FN_6O_7$: 626.6758.<br>Found: 627.2934 $[M+H]^+$<br>^{19}F NMR: δ (ppm) -117.52, -117.59
**Peptide 20**	**Ac-WPAA(4FPhe)-CONH$_2$**
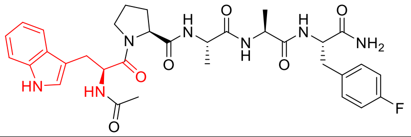	HPLC RT: 7.10 min<br>HPLC-MS (ESI+) Calc. $C_{33}H_{40}FN_7O_6$: 649.7124.<br>Found: 650.3095 $[M+H]^+$<br>^{19}F NMR: δ (ppm) -117.50, -117.60
**Peptide 21**	**Ac-YPGA(4FPhe)-CONH$_2$**
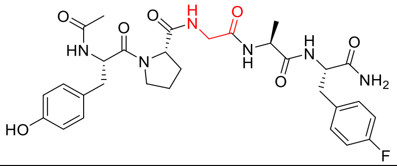	HPLC RT: 7.74 min[b]<br>HPLC-MS (ESI+) Calc. $C_{30}H_{37}FN_6O_7$: 612.6492.<br>Found: 613.2777 $[M+H]^+$<br>^{19}F NMR: δ (ppm) -116.38, -116.46
**Peptide 22**	**Ac-YPPA(4FPhe)-CONH$_2$**
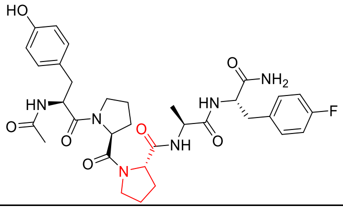	HPLC RT: 4.61 min<br>HPLC-MS (ESI+) Calc. $C_{33}H_{41}FN_6O_7$ 652.7130.<br>Found: 653.3093 $[M+H]^+$<br>^{19}F NMR: δ (ppm) -117.60, -117.61
**Peptide 23**	**Ac-YPIA(4FPhe)-CONH$_2$**
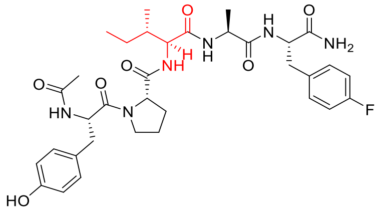	HPLC RT: 9.22 min[b]<br>HPLC-MS (ESI+) Calc. $C_{34}H_{45}FN_6O_7$: 668.7555.<br>Found: 669.3390 $[M+H]^+$<br>^{19}F NMR: δ (ppm) -116.39, -116.42
**Peptide 24**	**Ac-YPLA(4FPhe)-CONH$_2$**
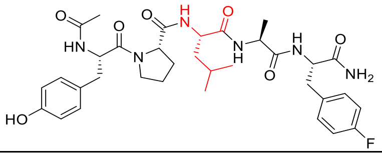	HPLC RT: 5.59 min<br>HPLC-MS (ESI+) Calc. $C_{34}H_{45}FN_6O_7$: 668.7555.<br>Found: 669.3401 $[M+H]^+$<br>^{19}F NMR: δ (ppm) -117.36, -117.43
**Peptide 25**	**Ac-YPVA(4FPhe)-CONH$_2$**
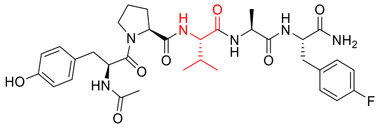	HPLC RT: 5.33 min<br>HPLC-MS (ESI+) Calc. $C_{33}H_{43}FN_6O_7$ 654.7289.<br>Found: 655.3254 $[M+H]^+$<br>^{19}F NMR: δ (ppm) -117.54, -117.57

**Peptide 26**	**Ac-YPMA(4FPhe)-$CONH_2$**
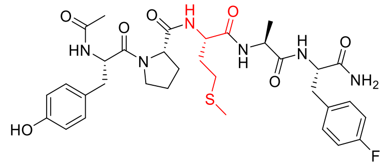	HPLC RT: 5.44 min<br>HPLC-MS (ESI+) Calc. $C_{33}H_{43}FN_6O_7S$: 686.7939.<br>Found: 687.2967 $[M+H]^+$<br>^{19}F NMR: δ (ppm) -117.43, -117.48
**Peptide 27**	**Ac-YPCA(4FPhe)-$CONH_2$**
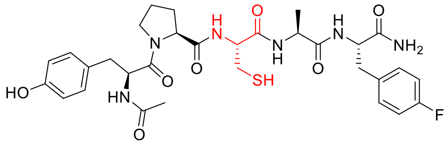	HPLC RT: 8.26 min[b]<br>HPLC-MS (ESI+) Calc. $C_{31}H_{39}FN_6O_7S$: 658.7408.<br>Found: 659.2657 $[M+H]^+$<br>^{19}F NMR: δ (ppm) -117.44, -117.48
**Peptide 28**	**Ac-YPSA(4FPhe)-$CONH_2$**
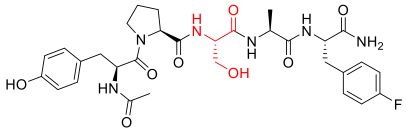	HPLC RT: 4.83 min<br>HPLC-MS (ESI+) Calc. $C_{31}H_{39}FN_6O_8$ 642.6752.<br>Found: 643.2890 $[M+H]^+$<br>^{19}F NMR: δ (ppm) -117.50, -117.56
**Peptide 29**	**Ac-YPTA(4FPhe)-$CONH_2$**
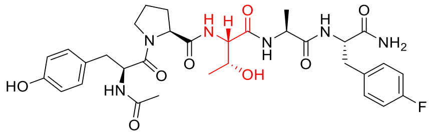	HPLC RT: 7.84 min[b]<br>HPLC-MS (ESI+) Calc. $C_{32}H_{41}FN_6O_8$ 656.7017.<br>Found: 657.3046 $[M+H]^+$<br>^{19}F NMR: δ (ppm) -117.51, -117.55
**Peptide 30**	**Ac-YPDA(4FPhe)-$CONH_2$**
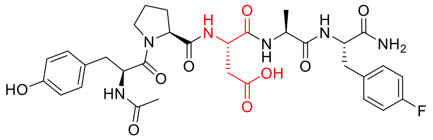	HPLC RT: 5.00 min<br>HPLC-MS (ESI+) Calc. $C_{32}H_{39}FN_6O_9$: 670.6853.<br>Found: 671.2830 $[M+H]^+$<br>^{19}F NMR: δ (ppm) -117.49, -117.57
**Peptide 31**	**Ac-YPEA(4FPhe)-$CONH_2$**
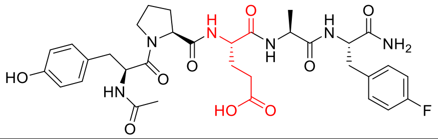	HPLC RT: 4.95 min<br>HPLC-MS (ESI+) Calc. $C_{33}H_{41}FN_6O_9$: 684.7118.<br>Found: 685.2986 $[M+H]^+$<br>^{19}F NMR: δ (ppm) -117.46, -117.53
**Peptide 32**	**Ac-YPNA(4FPhe)-$CONH_2$**
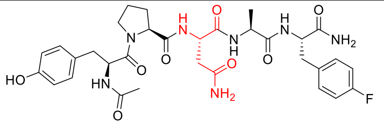	HPLC RT: 4.79 min<br>HPLC-MS (ESI+) Calc. $C_{32}H_{40}FN_7O_8$: 669.7005.<br>Found: 670.2992 $[M+H]^+$<br>^{19}F NMR: δ (ppm) -117.49, -117.57
**Peptide 33**	**Ac-YPQA(4FPhe)-$CONH_2$**
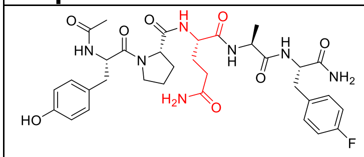	HPLC RT: 4.87 min<br>HPLC-MS (ESI+) Calc. $C_{33}H_{42}FN_7O_8$: 683.7271.<br>Found: 684.3147 $[M+H]^+$<br>^{19}F NMR: δ (ppm) -117.43, -117.50
**Peptide 34**	**Ac-YPRA(4FPhe)-$CONH_2$**
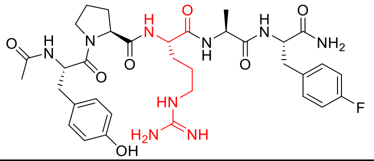	HPLC RT: 4.33 min<br>HPLC-MS (ESI+) Calc. $C_{34}H_{46}FN_9O_7$: 711.7835.<br>Found: 712.3583 $[M+H]^+$<br>^{19}F NMR: δ (ppm) -117.52, -117.55

**Peptide 35**	**Ac-YPKA(4FPhe)-CONH₂**
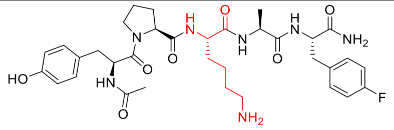	HPLC RT: 4.23 min<br>HPLC-MS (ESI+) Calc. $C_{34}H_{46}FN_7O_7$: 683.7701.<br>Found: 684.3511 $[M+H]^+$<br>^{19}F NMR: δ (ppm) -117.49, -117.53
**Peptide 36**	**Ac-YPHA(4FPhe)-CONH₂**
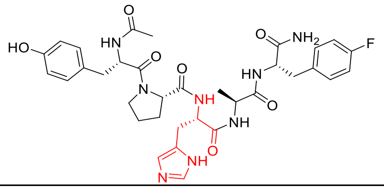	HPLC RT: 6.18 min[b]<br>HPLC-MS (ESI+) Calc. $C_{34}H_{41}FN_8O_7$: 692.7371.<br>Found: 693.3208 $[M+H]^+$<br>^{19}F NMR: δ (ppm) -116.35, -116.43
**Peptide 37**	**Ac-YPFA(4FPhe)-CONH₂**
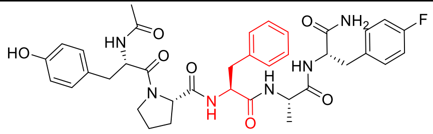	HPLC RT: 9.74 min[b]<br>HPLC-MS (ESI+) Calc. $C_{37}H_{43}FN_6O_7$: 702.7717.<br>Found: 703.3250 $[M+H]^+$<br>^{19}F NMR: δ (ppm) -116.28, -116.36
**Peptide 38**	**Ac-YPYA(4FPhe)-CONH₂**
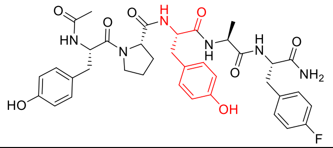	HPLC RT: 5.06 min<br>HPLC-MS (ESI+) Calc. $C_{37}H_{43}FN_6O_8$ 718.7711.<br>Found: 719.3197 $[M+H]^+$<br>^{19}F NMR: δ (ppm) -117.42, -117.49
**Peptide 39**	**Ac-YPWA(4FPhe)-CONH₂**
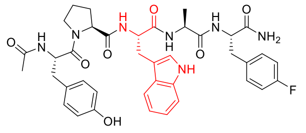	HPLC RT: 8.20 min[b]<br>HPLC-MS (ESI+) Calc. $C_{39}H_{44}FN_7O_7$ 741.8078.<br>Found: 742.3401 $[M+H]^+$<br>^{19}F NMR: δ (ppm) -117.42, -117.53

1.3 Probing the effect of pH

Peptides based on those reported by Dyson *et al* [1] but containing 4-fluorophenyl-alanine were analysed in triplicate (entries a - c) by ^{19}F NMR (in ppm) at each pH.

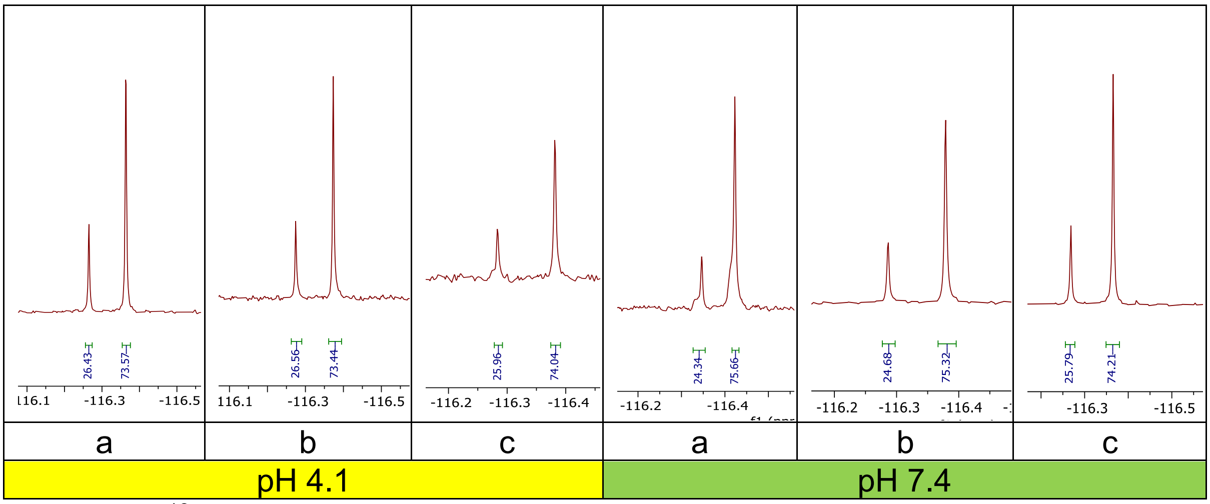

Figure S3. ^{19}F NMR (pH 4.1, pH 7.4) Ac-YPDD(4F-Phe)-$CONH_2$ **(Peptide 40)**.

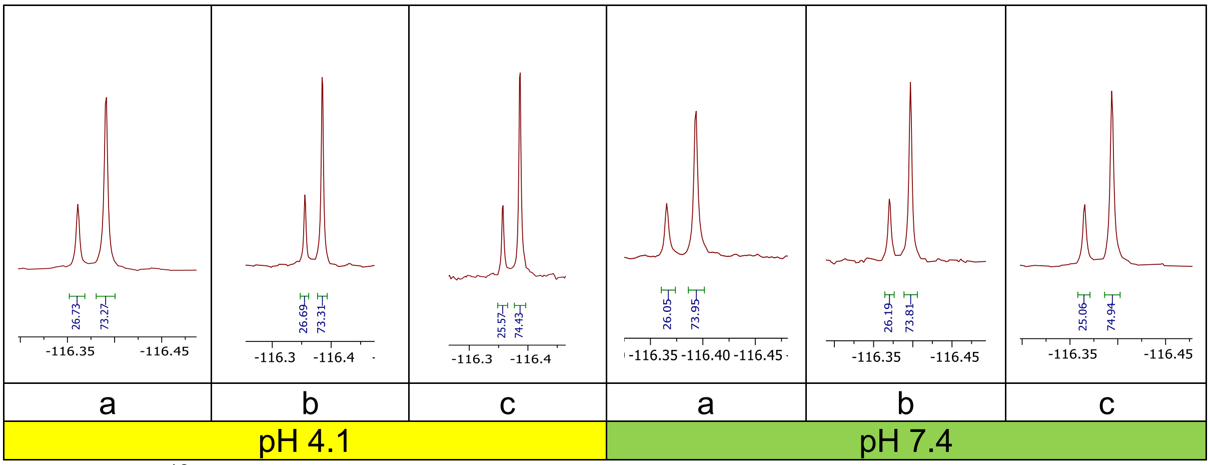

Figure S4. ^{19}F NMR (pH 4.1, pH 7.4) Ac-YPHD(4FPhe)-$CONH_2$ **(Peptide 41)**.

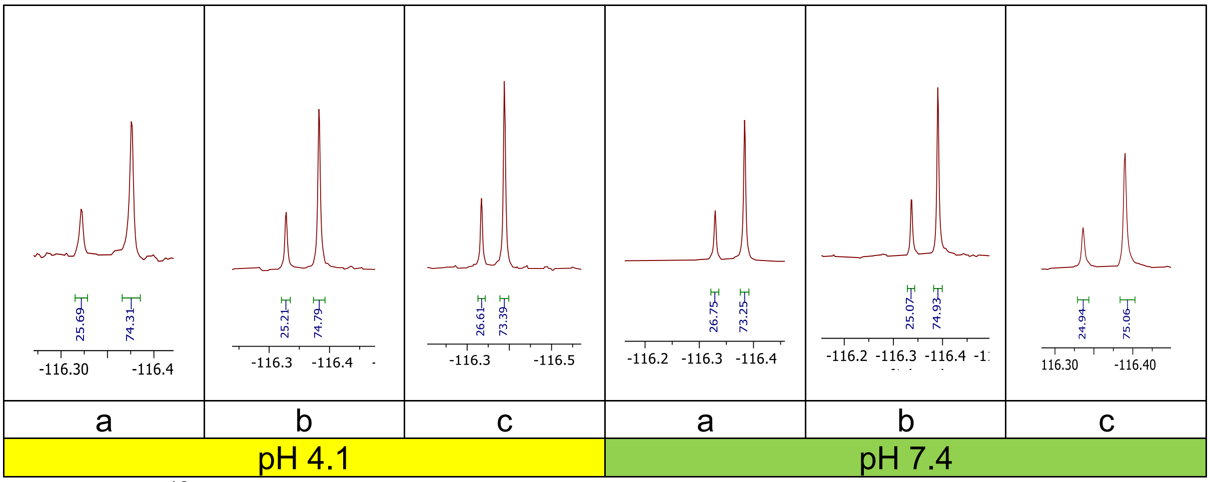

Figure S5. ^{19}F NMR (pH 4.1, pH 7.4) Ac-YPKD(4FPhe)-$CONH_2$ **(Peptide 42)**

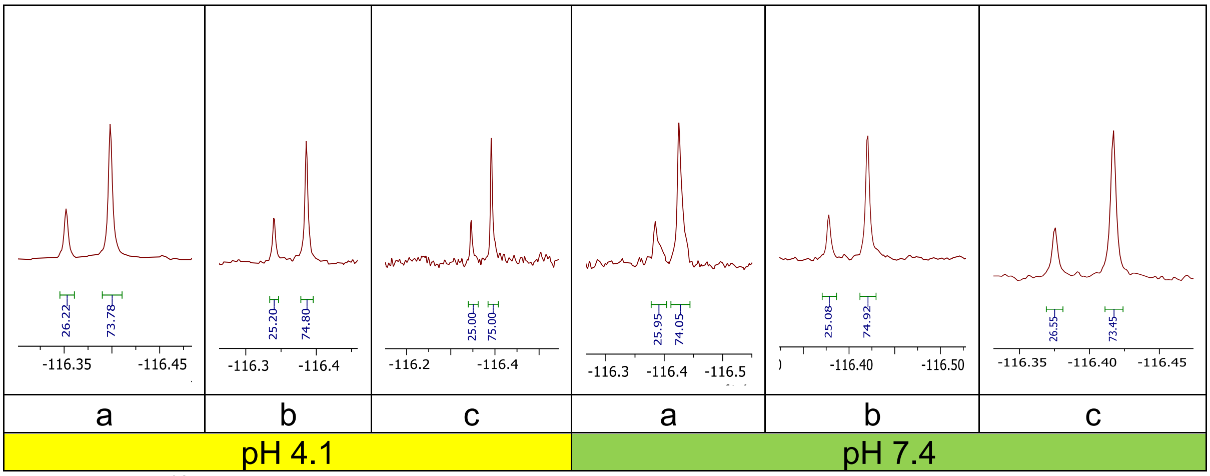

Figure S6. ^{19}F NMR (pH 4.1, pH 7.4) Ac-YPRD(4FPhe)-$CONH_2$ **(Peptide 43)**.

1.4 Probing the effect of temperature on *cis*-Pro

In reports of small proline models (e.g. Ac(F)ProOMe) the effect of temperature is noticeable – the *cis:trans* tends towards lower $K_{trans/cis}$, [2] in principle eventually reaching 1:1 ratio at a high temperature. However, in a peptide model [3] the *cis:trans* populations were found to be independent of temperature between 5 °C and 70 °C. To probe this using ^{19}F NMR, we subjected our peptide models Ac-LPAA(4F-Phe)-$CONH_2$ and Ac-YPGA(4F-Phe)-$CONH_2$ in pH 7.4 buffer to a variable temperature NMR experiment between 10 ºC – 60 ºC in 10 ºC steps. In our hands, the model peptide AcLPAA(4FPhe) – poorly resolved to begin with, was found to appear to coalesce towards one resonance with increasing temperature and correspondingly, the % *cis*-Pro after line shaping analysis appeared to slightly increase. Conversely and perhaps unexpectedly, the peptide AcYPGA(4FPhe) seemed to trend towards marginally lower % *cis*-Pro.

It is worth noting an up-field shift was observed in each series of experiments with increasing temperature.

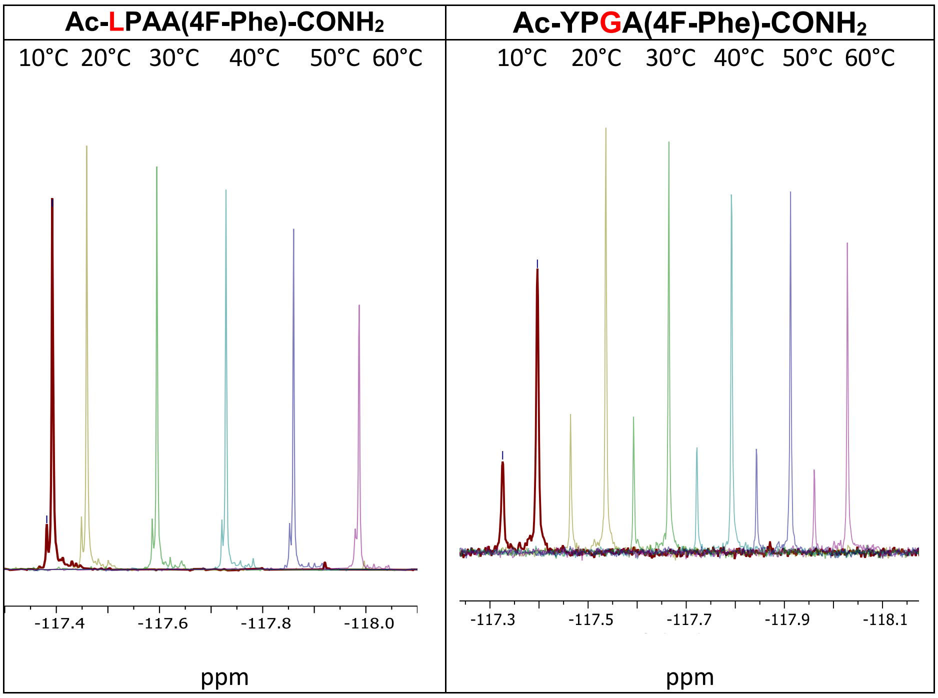

Figure S7. *cis*-Pro and *trans*-Pro (%) as a function of temperature (spectra referenced to TFA (-76.55 ppm))

Table S3. *cis*-Pro (%) as a function of temperature.

Ac-LPAA(4F-Phe)-$CONH_2$		Ac-YPGA(4F-Phe)-$CONH_2$	
°C	%*cis*-Pro *	°C	%*cis*-Pro
10	7	10	26
20	7	20	24
30	7	30	24
40	9	40	22
50	11	50	23
60	12	60	22

*after line-shaping analysis

1.5 α-Synuclein C-terminal region model pentapeptides

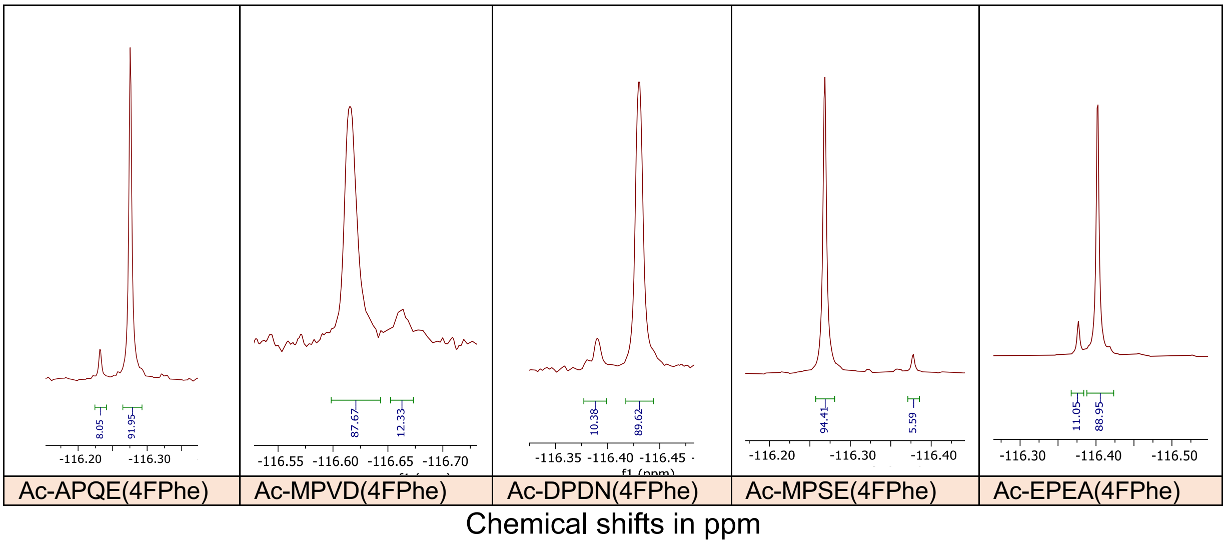

Figure S8. ^{19}F NMR spectra for the 5 model peptides.

1.6 Probing the effect of NaCl concentration (0 to 1.2 M NaCl)

Ac-APAA(4FPhe)-$CONH_2$ (Peptide 2)

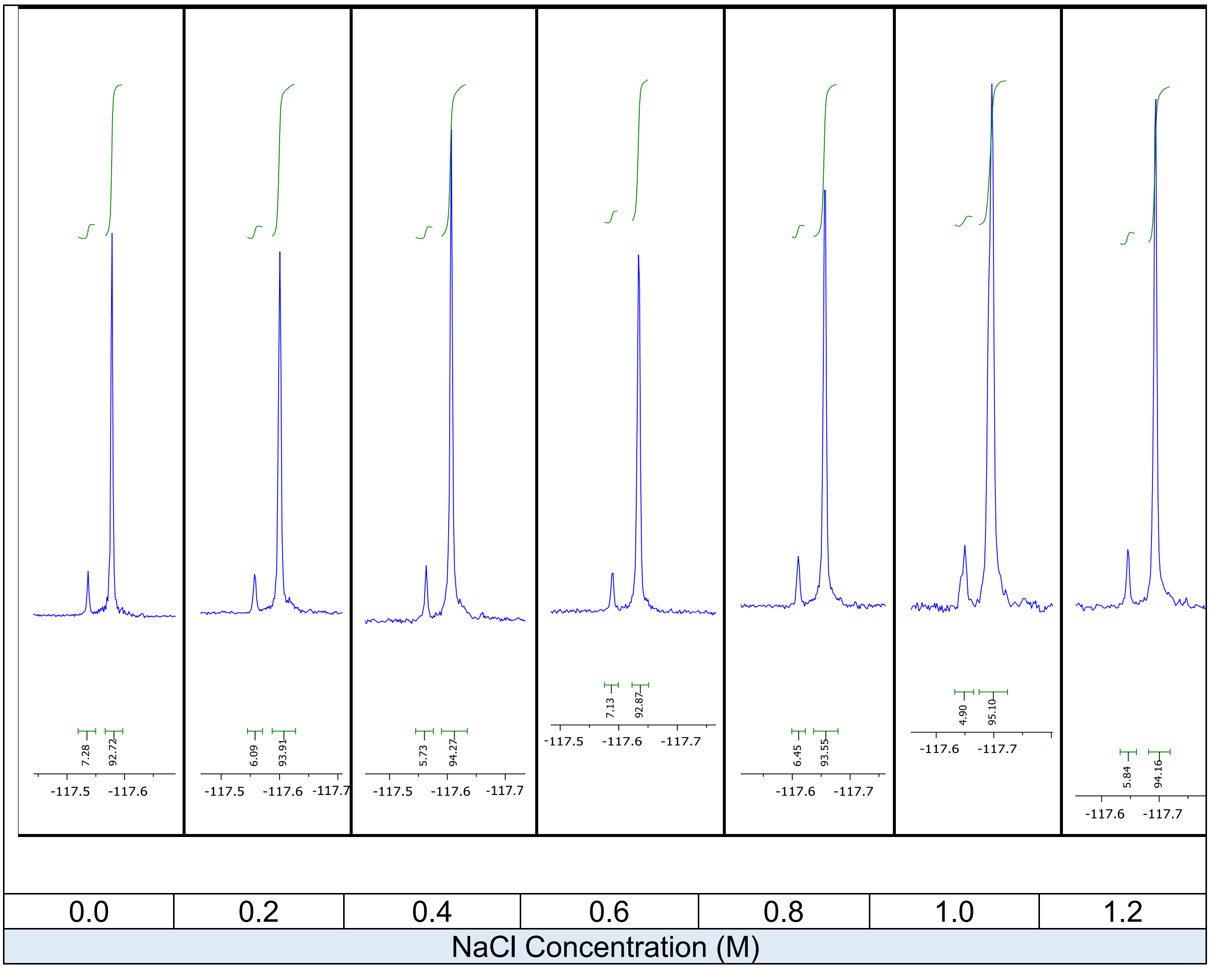

Figure S9. Stacked ^{19}F NMR spectra at 0 – 1.2 M NaCl

Ac-EPAA(4FPhe)-CONH$_2$ (Peptide 12)

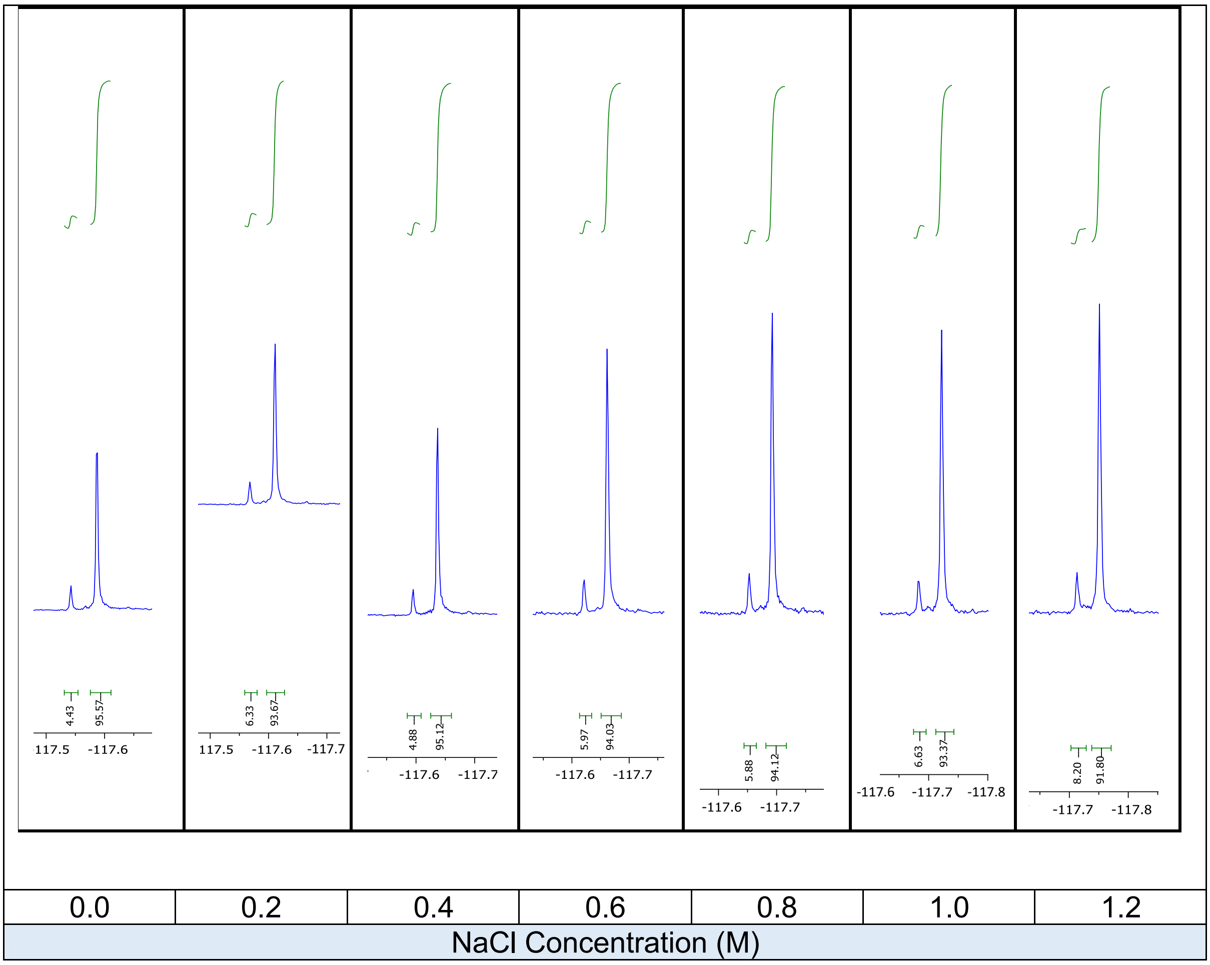

Figure S10. Stacked ^{19}F NMR spectra at 0 – 1.2 M NaCl

Ac-KPAA(4FPhe)-CONH$_2$ (Peptide 16)

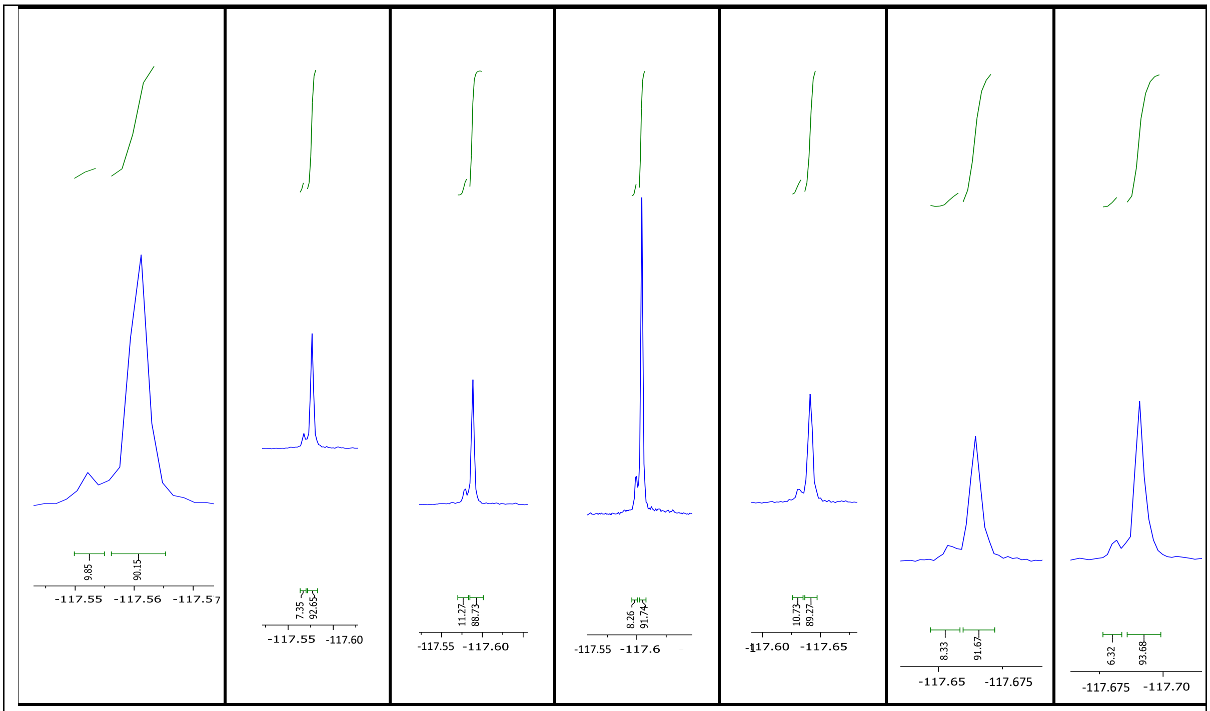

0.0	0.2	0.4	0.6	0.8	1.0	1.2
NaCl Concentration (M)						

Figure S11. Stacked ^{19}F NMR spectra at 0 – 1.2 M NaCl

Ac-YPAA(4FPhe)-CONH$_2$ (Peptide 19)

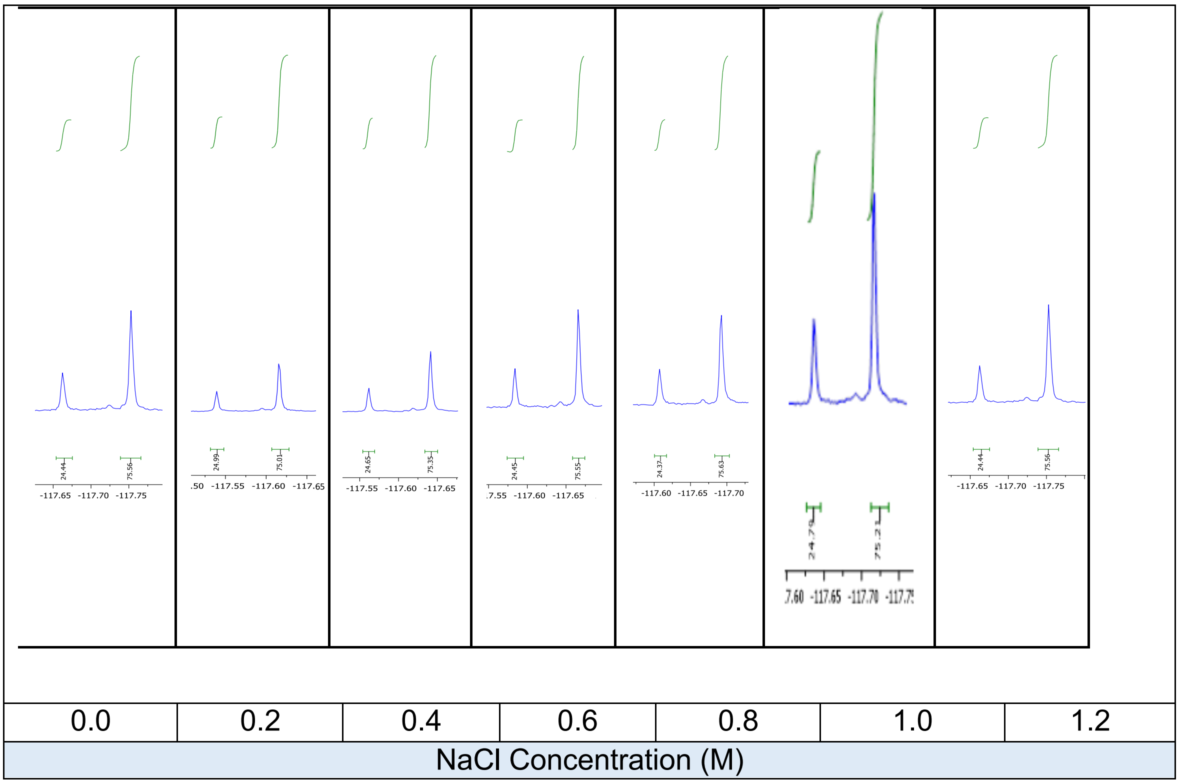

Figure S12. Stacked ^{19}F NMR spectra at 0 – 1.2 M NaCl

1.7 Probing the effect of DMSO on *cis*-Pro %

Incidentally, we also observed there was a noticeable deleterious effect of the addition of higher % DMSO to solubilise peptide models (supporting information), so DMSO was avoided subsequently. Therefore, all peptides were dissolved in aqueous buffer for ^{19}F NMR analysis.

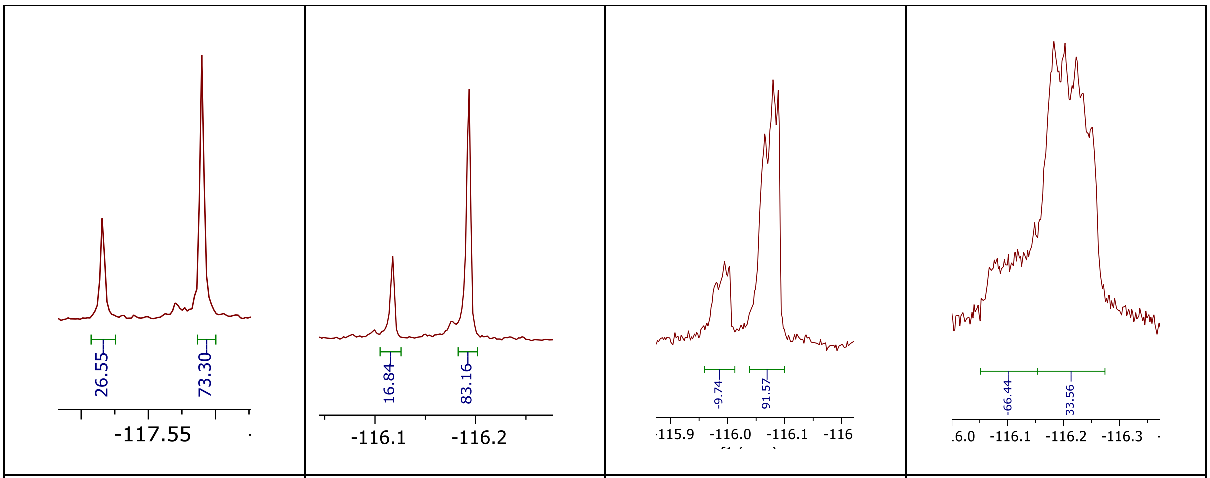

0%	10%	20%	40%
DMSO Concentration			

Figure S13. The effect of %DMSO on measured %*cis* prolyl conformer. Stacked ^{19}F NMR spectra for peptide Ac-YPAA(4FPhe)-NH_2. Chemical shifts in ppm.

2. APPENDIX

2.1 Analytical data for AcYP(A)$_n$(4FPhe) model peptides

Ac-YP(4FPhe)-NH$_2$

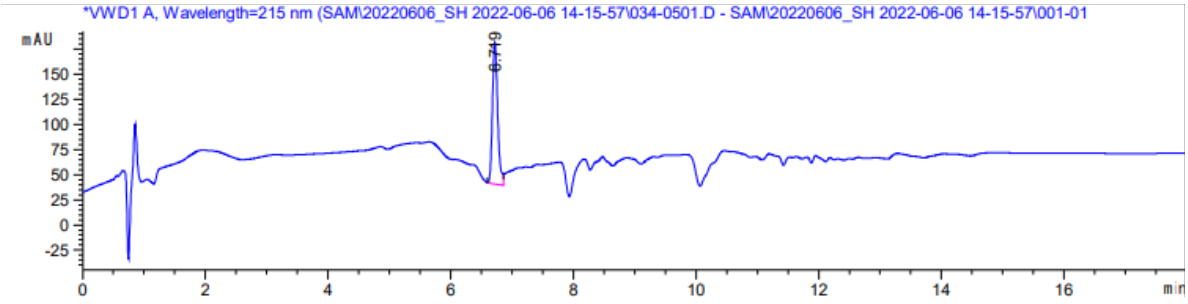

Figure S14. Analytical HPLC.

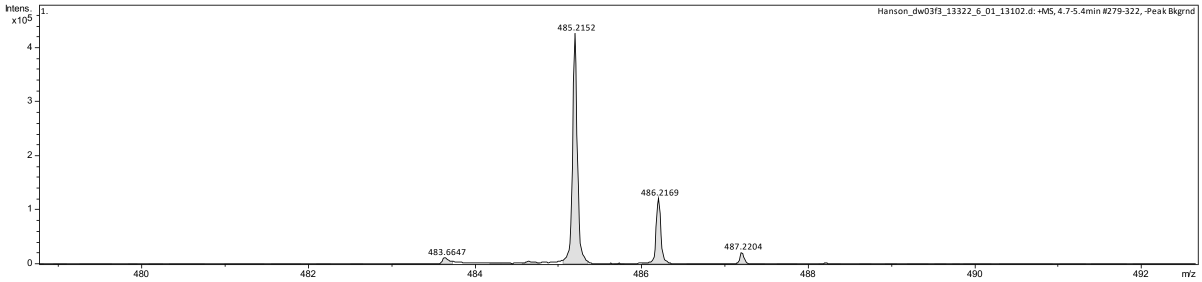

Figure S15. Mass spectrum ([M+H]$^+$). Peptide mass: 484.2122.

Ac-YPA(4FPhe)-NH$_2$

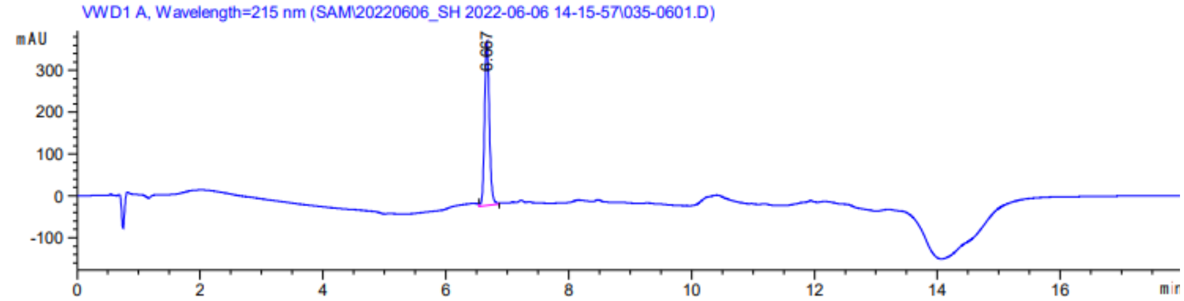

Figure S16. Analytical HPLC.

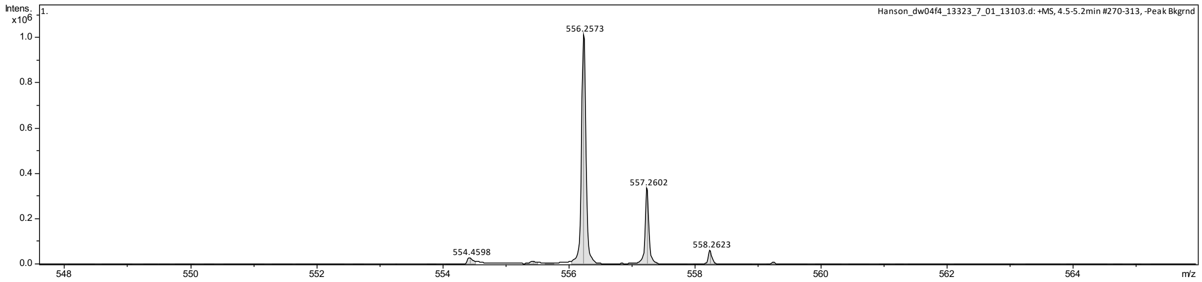

Figure S17. Mass spectrum ([M+H]$^+$). Peptide mass: 555.2493.

Ac-YPAAA(4FPhe)-NH_2

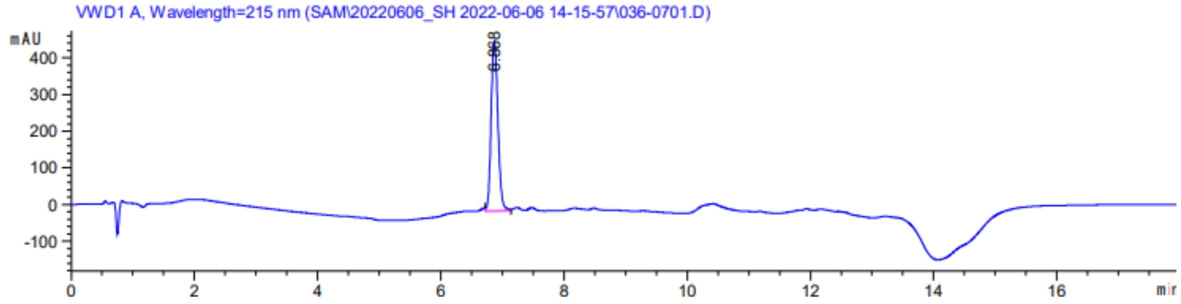

Figure S18. Analytical HPLC.

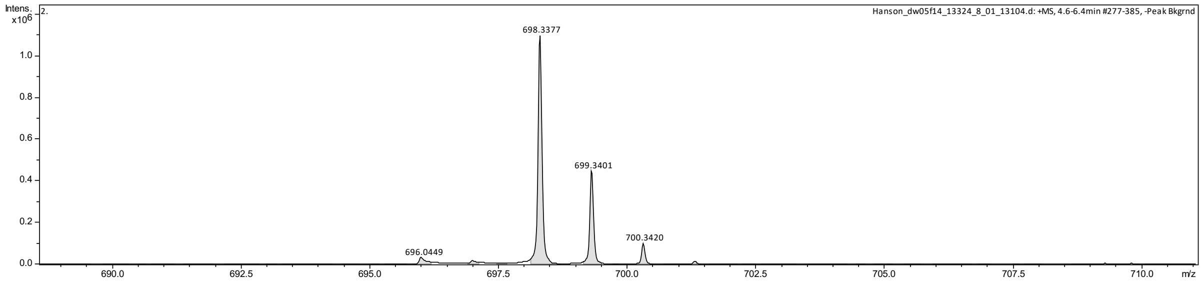

Figure S19. Mass spectrum ($[M+H]^+$). Peptide mass: 697.3235.

Ac-YPAAAA(4FPhe)-NH_2

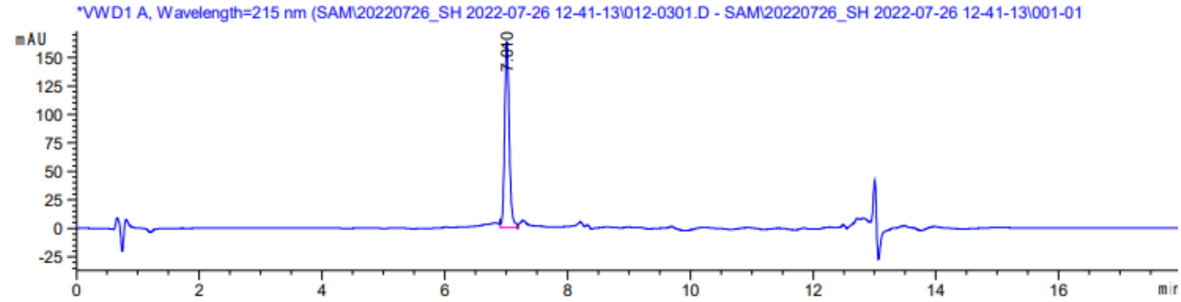

Figure S20. Analytical HPLC.

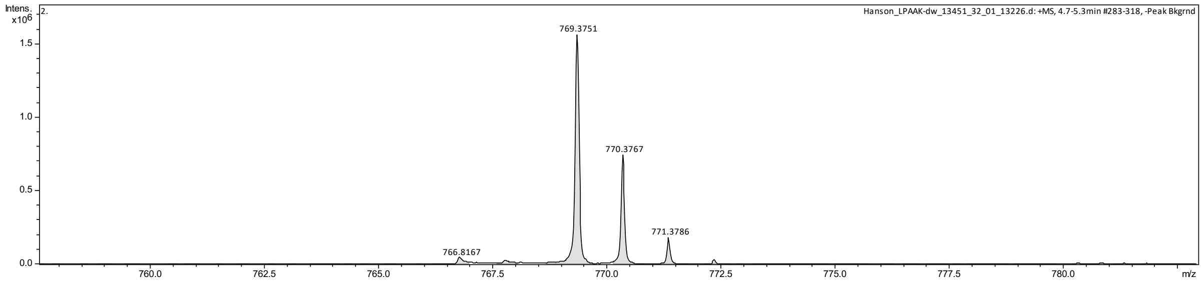

Figure S21. Mass spectrum ($[M+H]^+$). Peptide mass: 768.3607.

Ac-YPAAAAA(4FPhe)-NH2

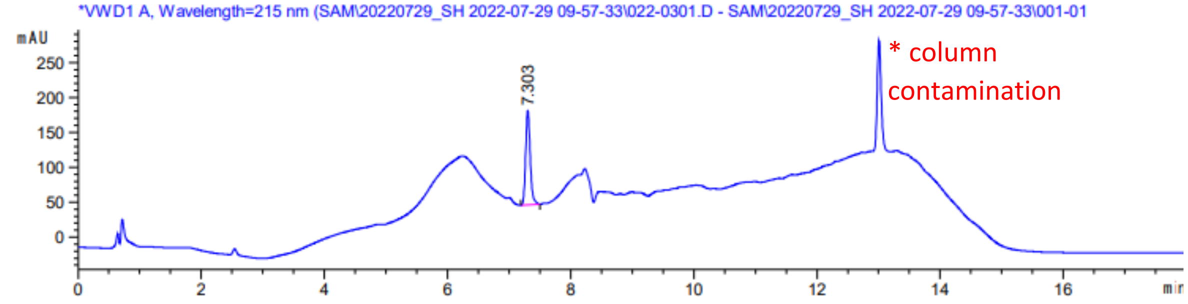

Figure S22. Analytical HPLC.

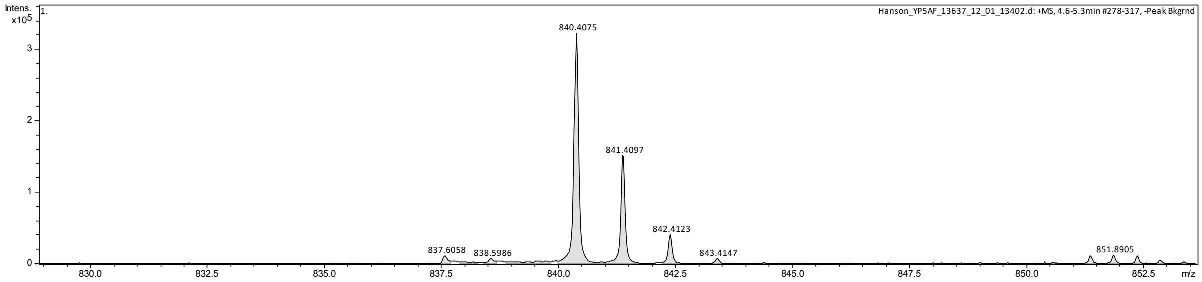

Figure S23. Mass spectrum ($[M+H]^+$). Peptide mass: 839.3978.

Ac-YPAAAAAA(4FPhe)-NH2

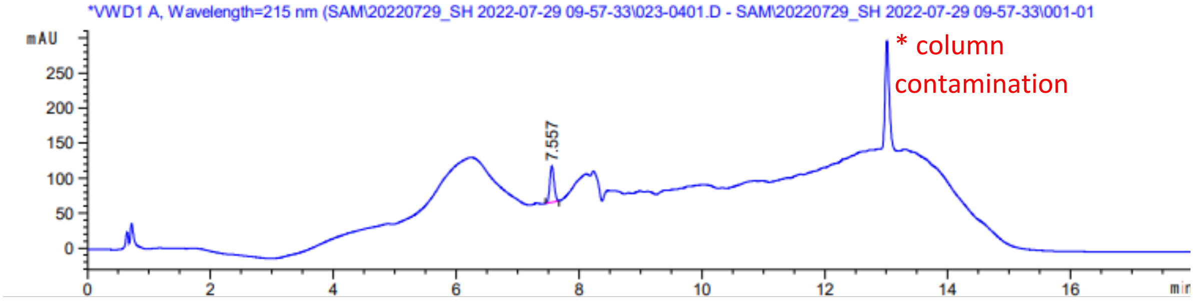

Figure S24. Analytical HPLC.

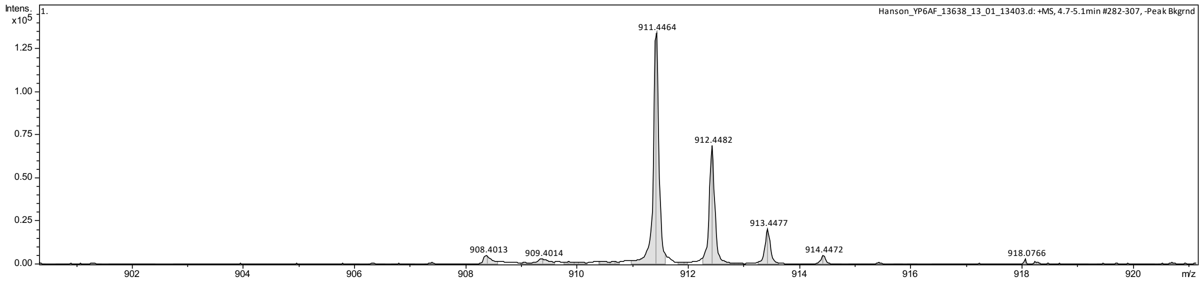

Figure S25. Mass spectrum ($[M+H]^+$). Peptide mass: 910.4349.

Ac-YPAAAAAAA(4FPhe)-NH2

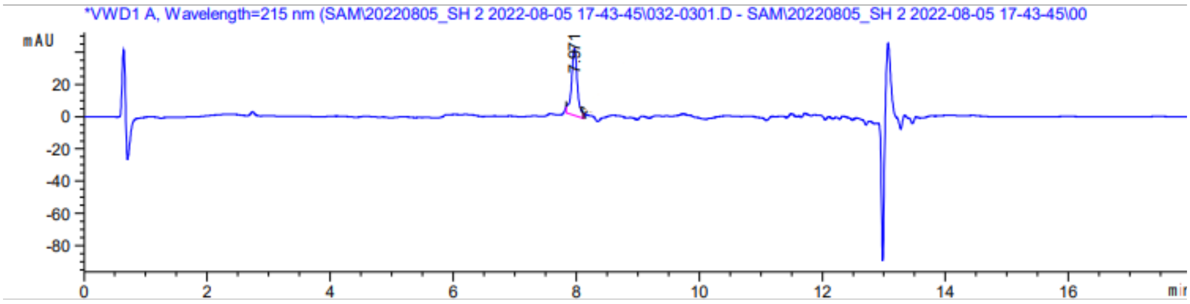

Figure S26. Analytical HPLC.

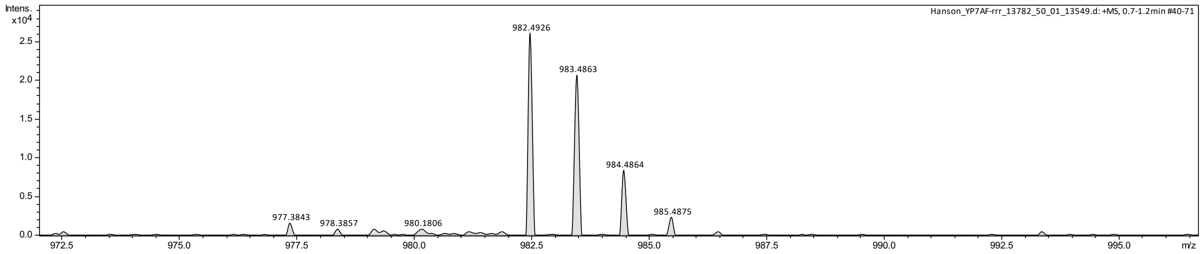

Figure S27. Mass spectrum ($[M+H]^+$). Peptide mass: 981.4720.

Ac-YAAA(4FPhe)-NH2

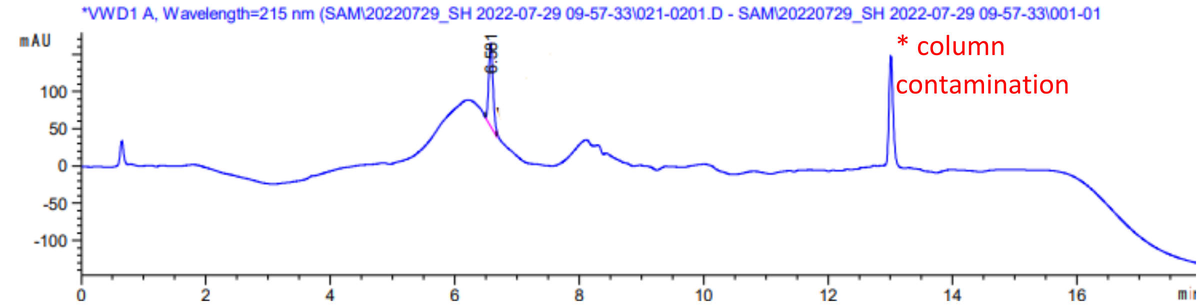

Figure S28. Analytical HPLC.

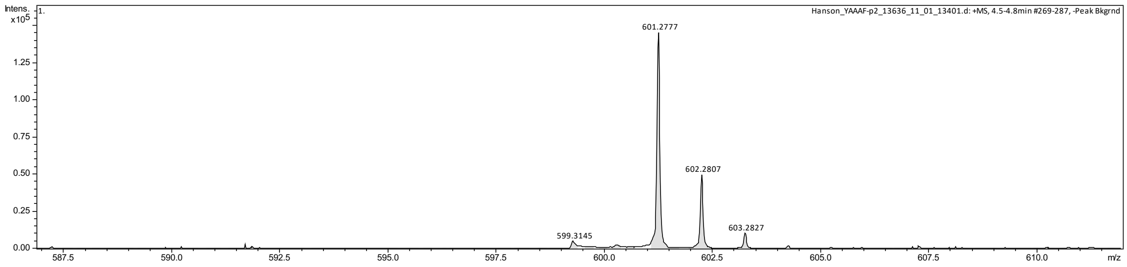

Figure S29. Mass spectrum ($[M+H]^+$). Peptide mass: 600.2708.

2.2 Analytical data for X-Pro-Z model peptides

Ac-GPAA(4FPhe)-CONH$_2$ (Peptide 1)

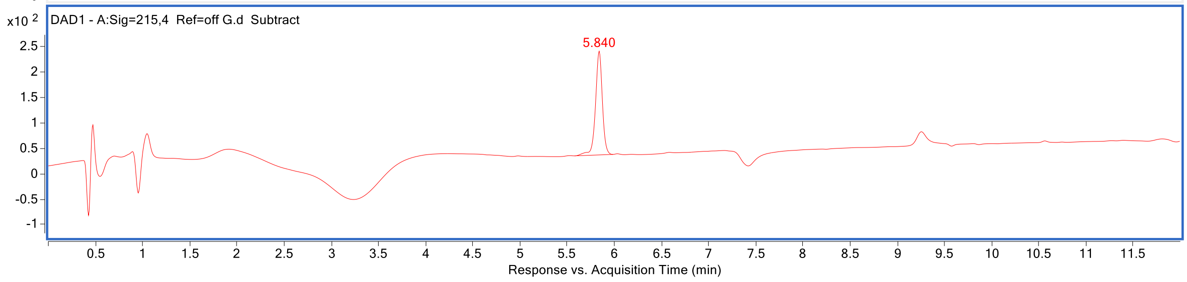

Figure S30. Analytical HPLC.

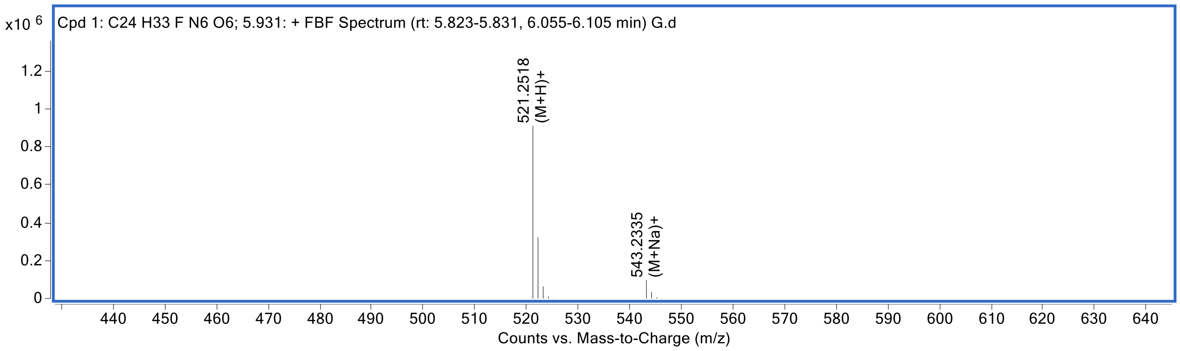

Figure S31. Mass spectrum.

Ac-APAA(4FPhe)-CONH$_2$ (Peptide 2)

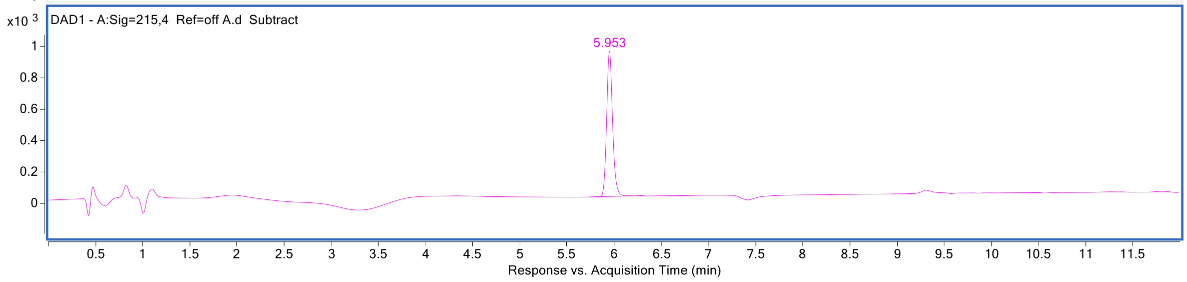

Figure S32. Analytical HPLC.

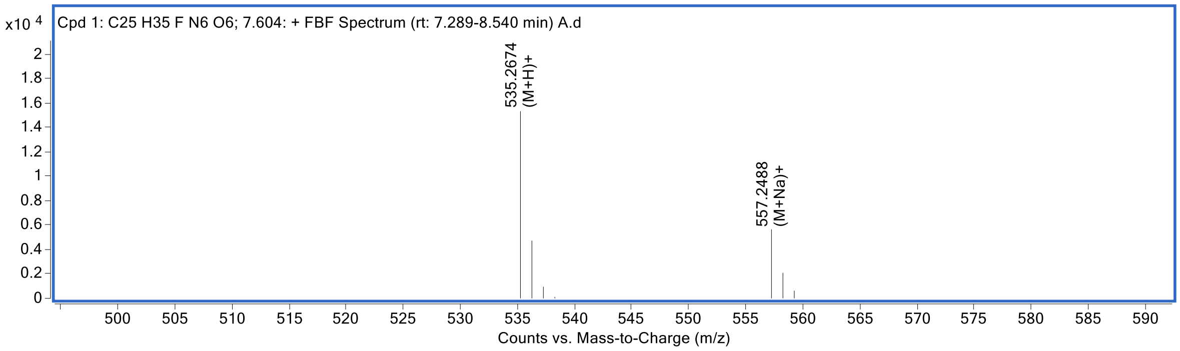

Figure S33. Mass spectrum.

H_2N-PPAA(4FPhe)-$CONH_2$ (Peptide 3)

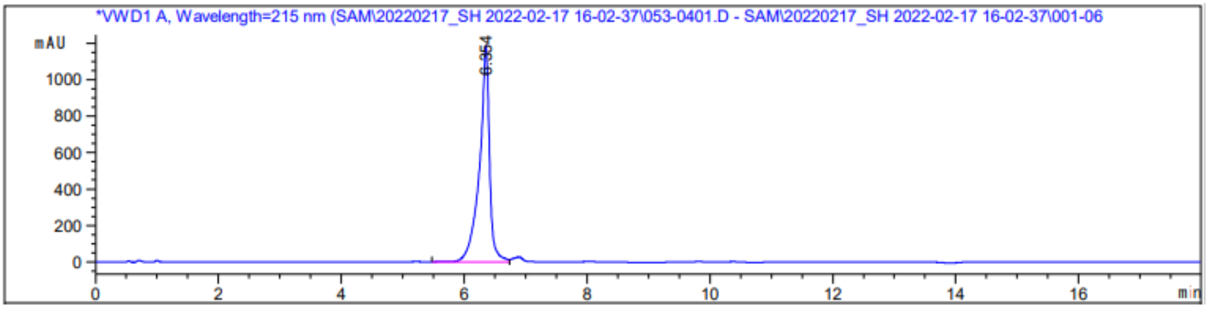

Figure S34. Analytical HPLC.

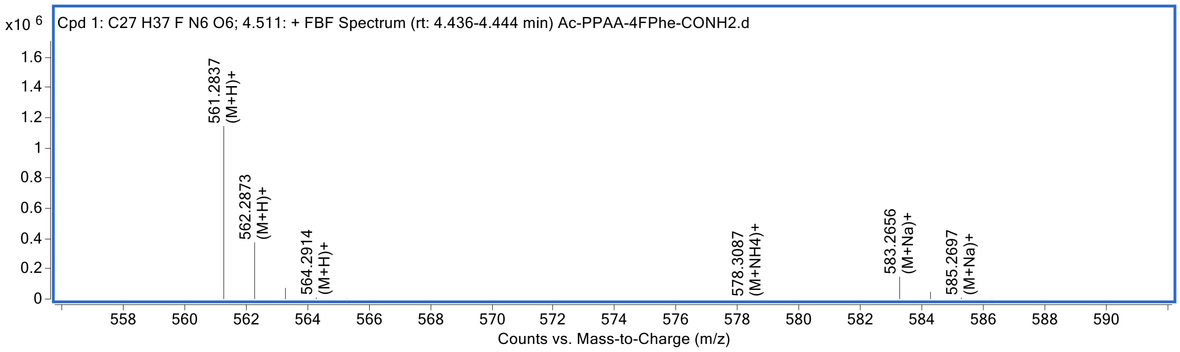

Figure S35. Mass spectrum.

Ac-IPAA(4FPhe)-$CONH_2$ (Peptide 4)

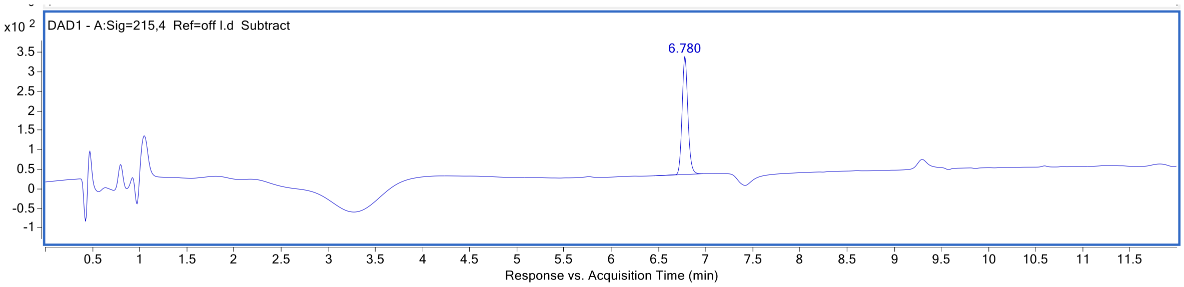

Figure S36. Analytical HPLC.

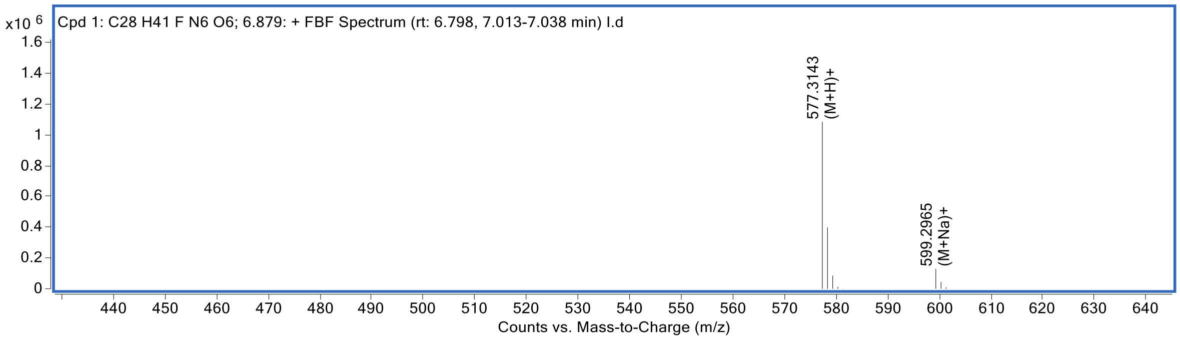

Figure S37. Mass spectrum.

Ac-LPAA(4FPhe)-$CONH_2$ (Peptide 5)

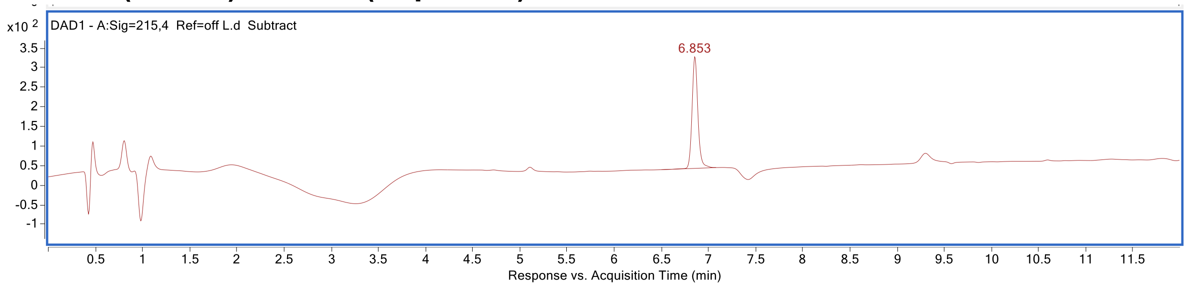

Figure S38. Analytical HPLC.

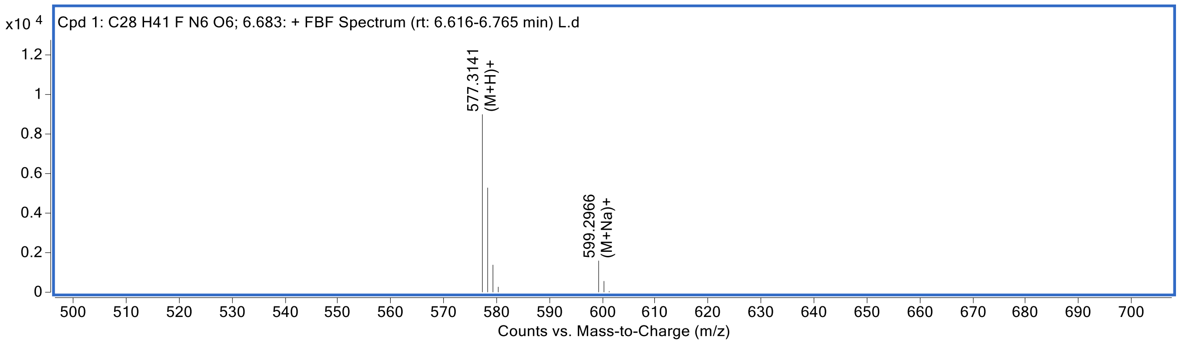

Figure S39. Mass spectrum.

Ac-VPAA(4FPhe)-$CONH_2$ (Peptide 6)

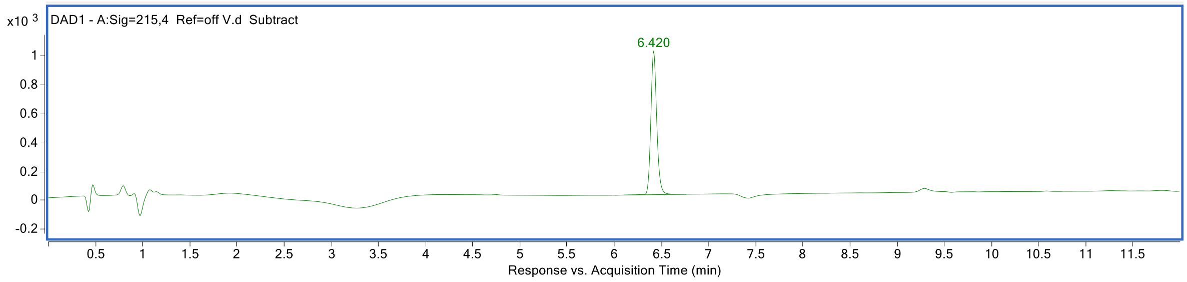

Figure S40. Analytical HPLC.

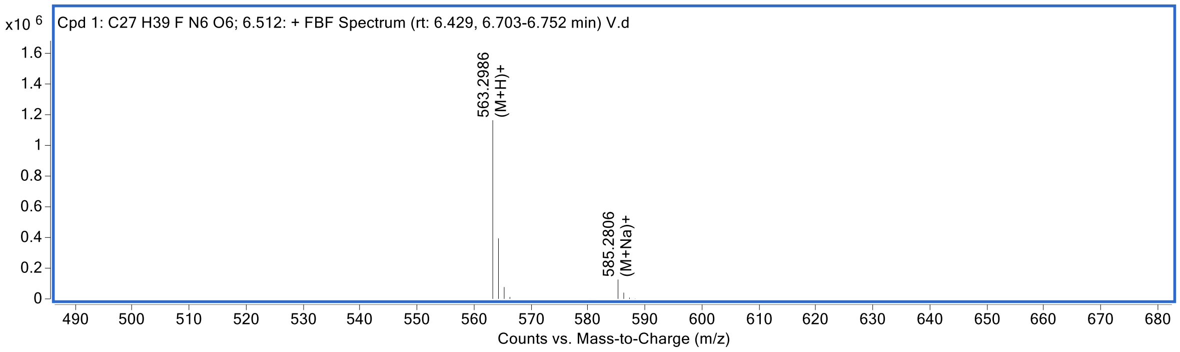

Figure S41. Mass spectrum.

Ac-**M**PAA(4FPhe)-CONH$_2$ (Peptide 7)

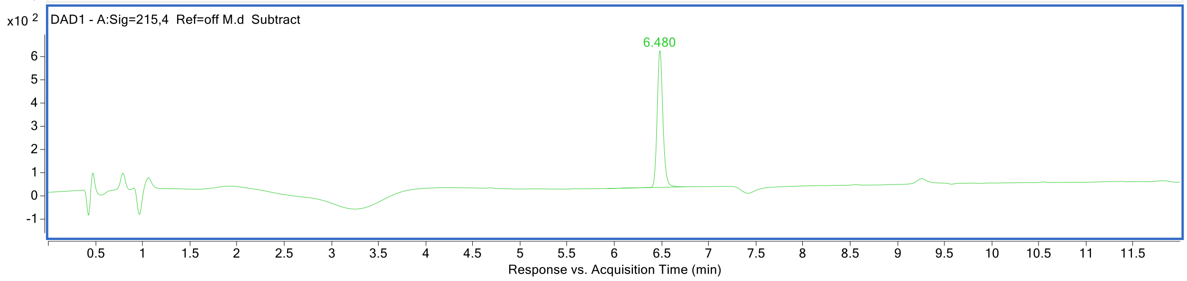

Figure S42. Analytical HPLC.

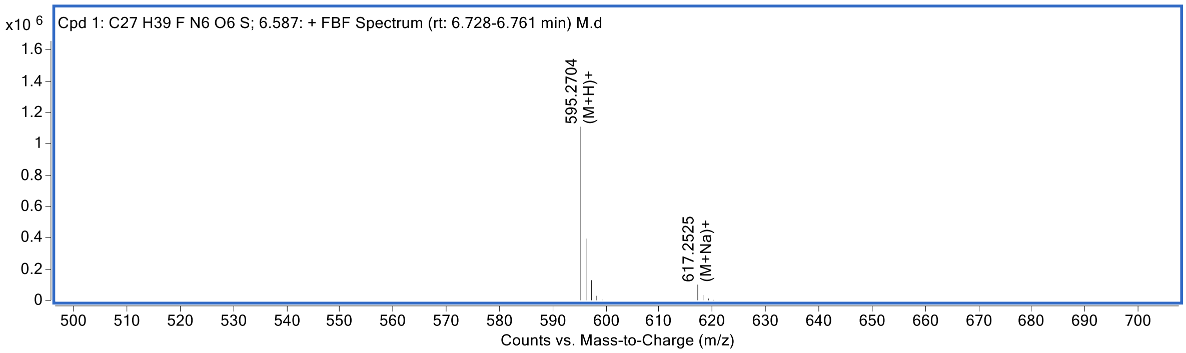

Figure S43. Mass spectrum.

Ac-**C**PAA(4FPhe)-CONH$_2$ (Peptide 8)

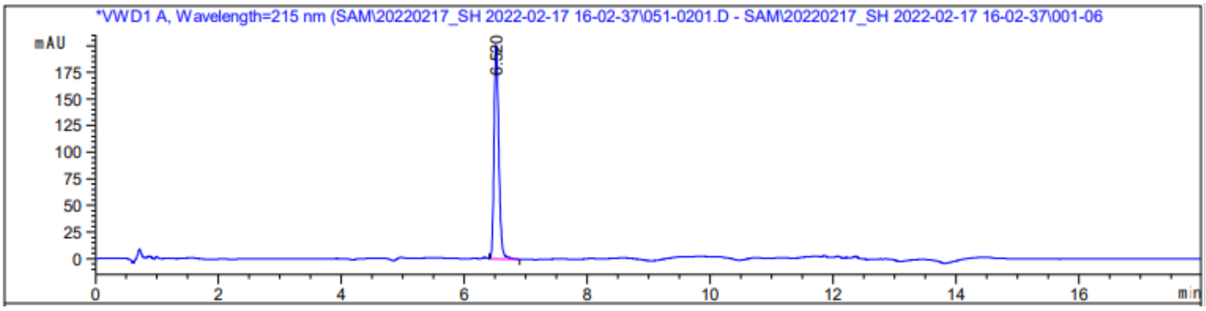

Figure S44. Analytical HPLC.

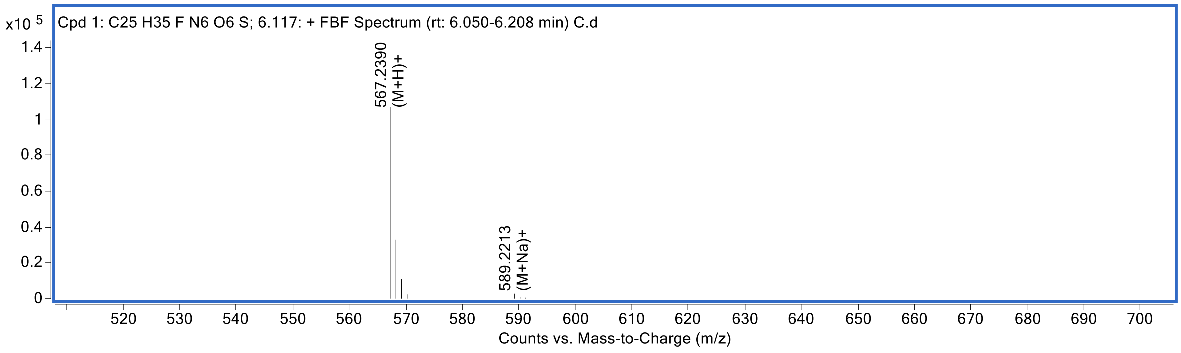

Figure S45. Mass spectrum.

Ac-SPAA(4FPhe)-CONH$_2$ (Peptide 9)

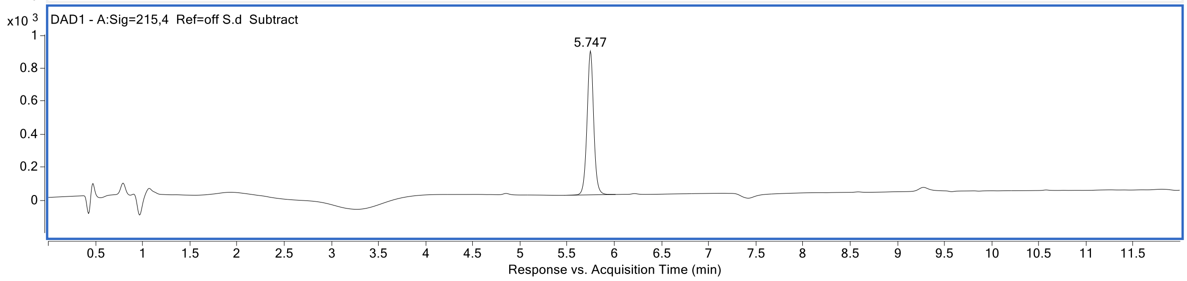

Figure S46. Analytical HPLC.

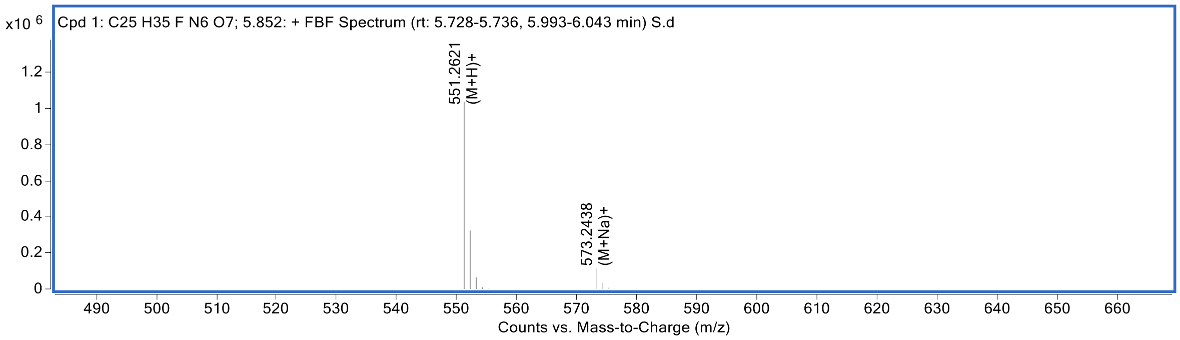

Figure S47. Mass spectrum.

Ac-TPAA(4FPhe)-CONH$_2$ (Peptide 10)

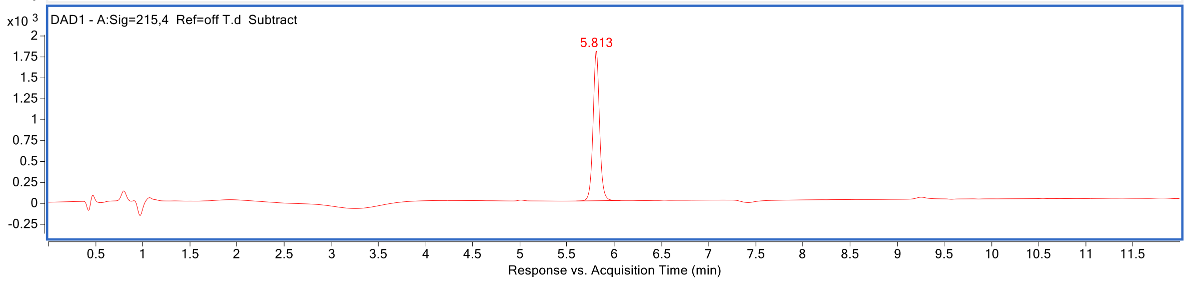

Figure S48. Analytical HPLC.

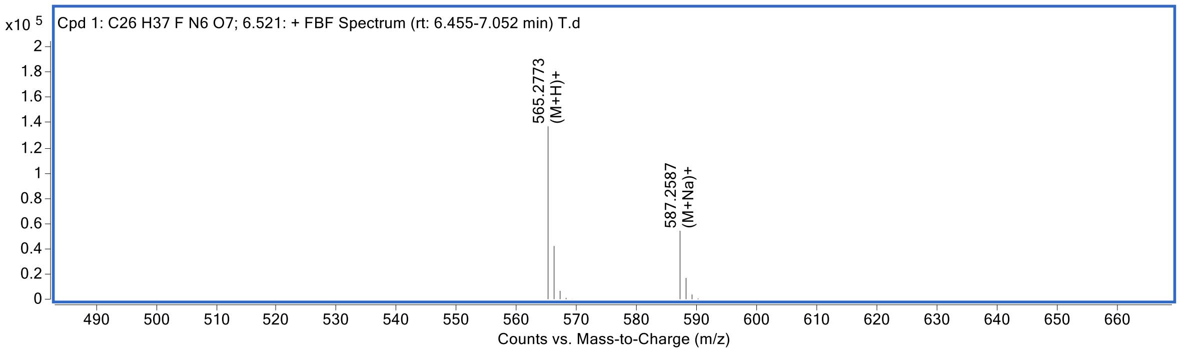

Figure S49. Mass spectrum.

Ac-DPAA(4FPhe)-CONH$_2$ (Peptide 11)

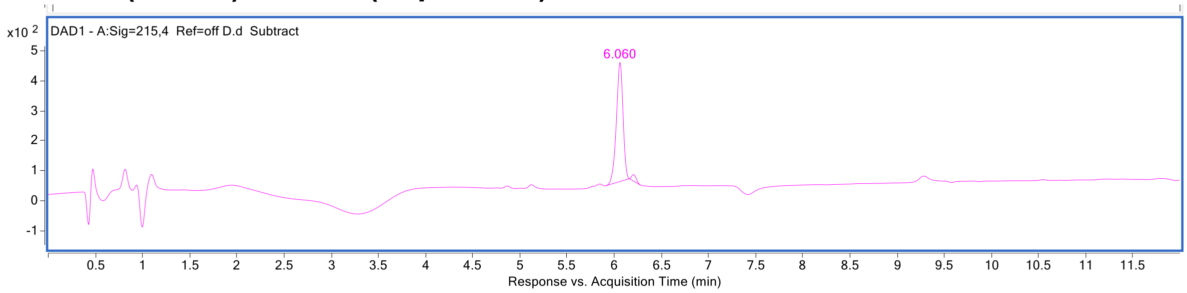

Figure S50. Analytical HPLC.

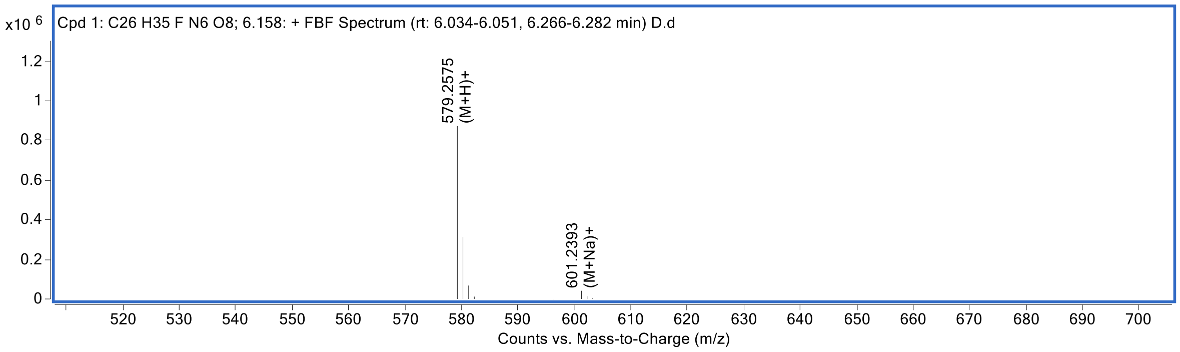

Figure S51. Mass spectrum.

Ac-EPAA(4FPhe)-CONH$_2$ (Peptide 12)

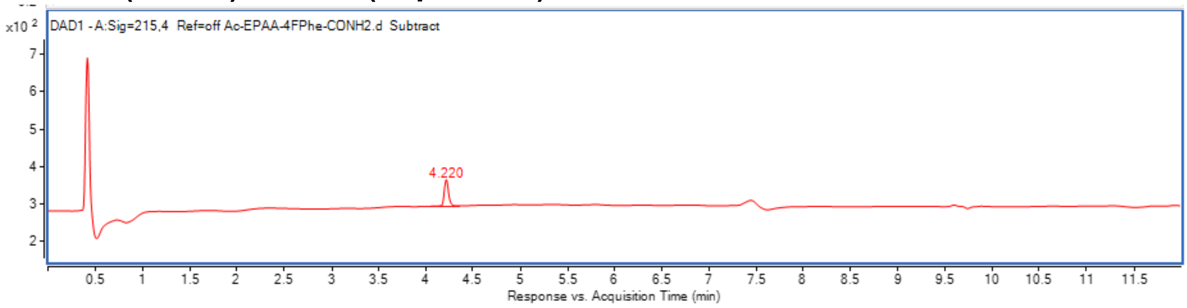

Figure S52. Analytical HPLC.

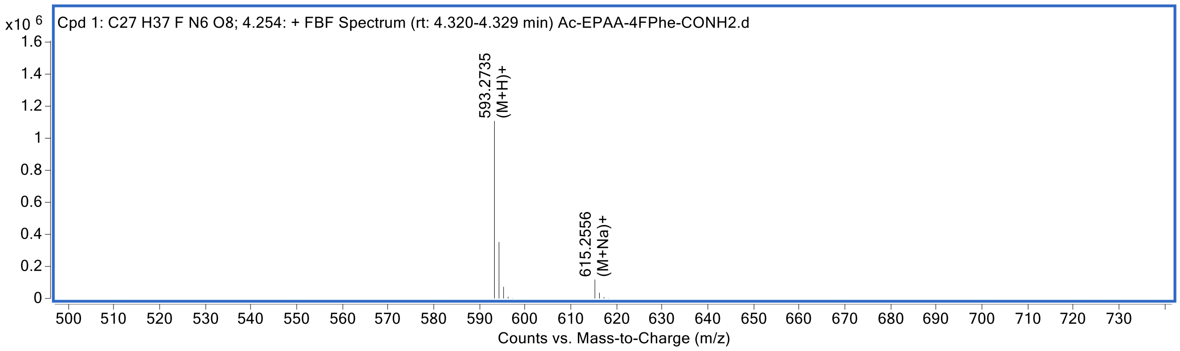

Figure S53. Mass spectrum.

Ac-NPAA(4FPhe)-CONH$_2$ (Peptide 13)

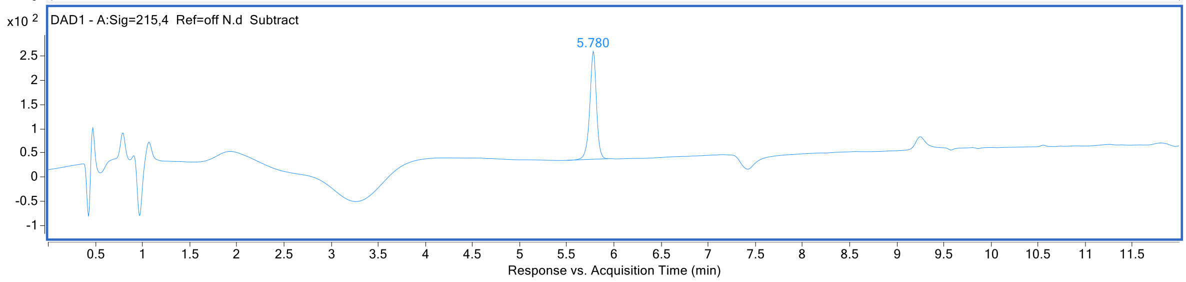

Figure S54. Analytical HPLC.

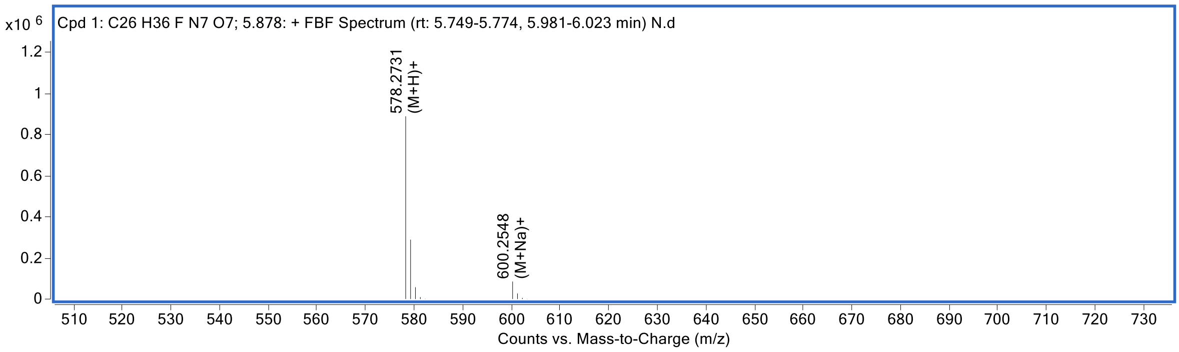

Figure S55. Mass spectrum.

Ac-QPAA(4FPhe)-CONH$_2$ (Peptide 14)

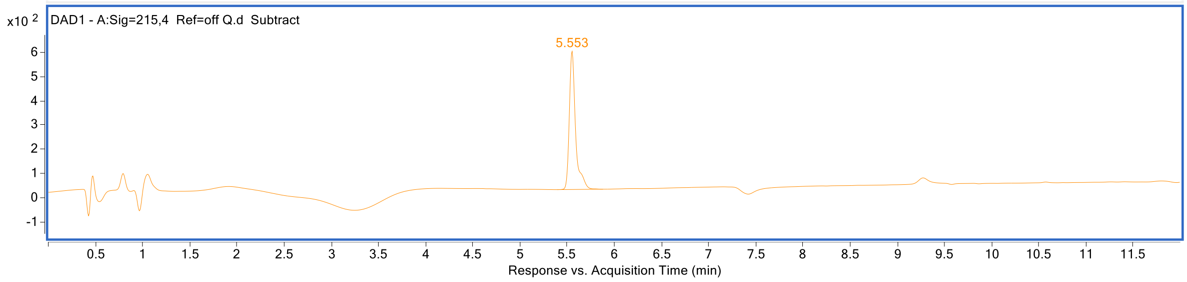

Figure S56. Analytical HPLC.

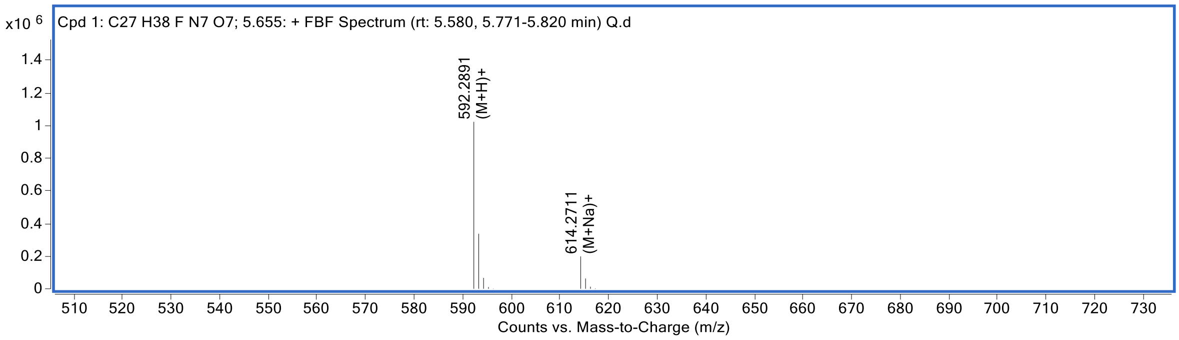

Figure S57. Mass spectrum.

Ac-RPAA(4FPhe)-CONH$_2$ (Peptide 15)

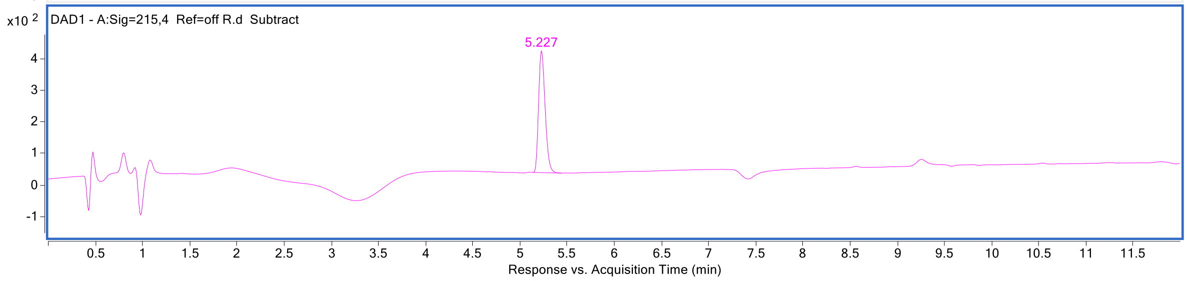

Figure S58. Analytical HPLC.

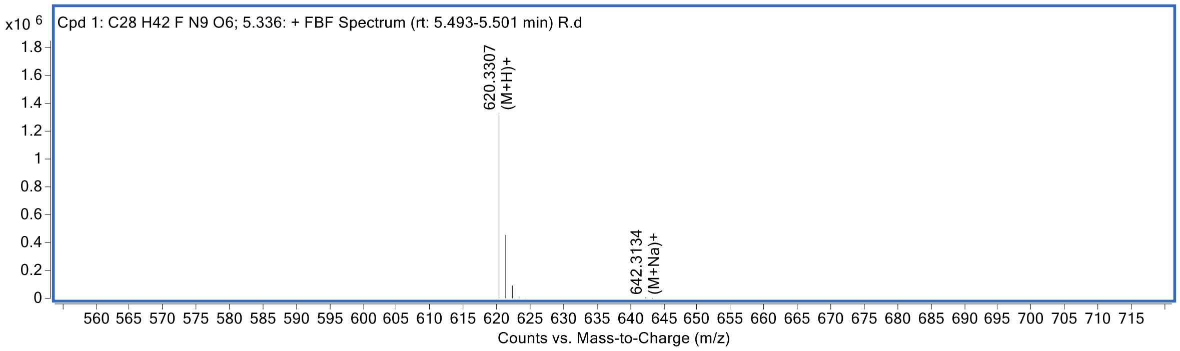

Figure S59. Mass spectrum.

Ac-KPAA(4FPhe)-CONH$_2$ (Peptide 16)

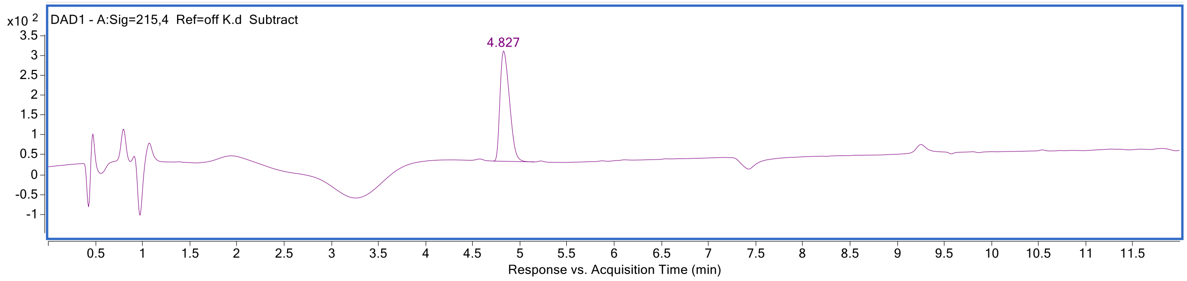

Figure S60. Analytical HPLC.

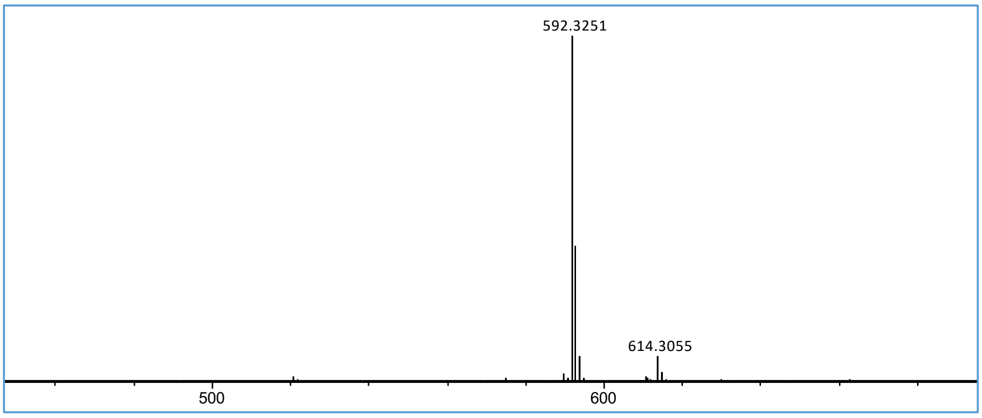

Figure S61. Mass spectrum.

Ac-HPAA(4FPhe)-CONH$_2$ (Peptide 17)

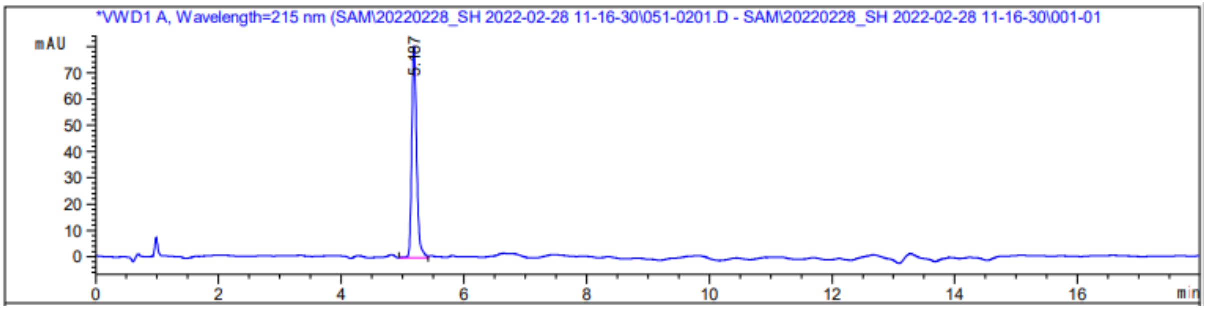

Figure S62. Analytical HPLC.

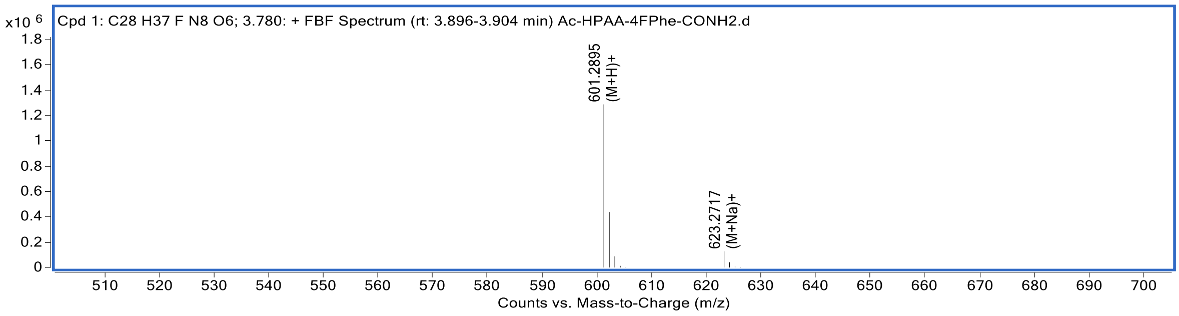

Figure S63. Mass spectrum.

Ac-FPAA(4FPhe)-CONH$_2$ (Peptide 18)

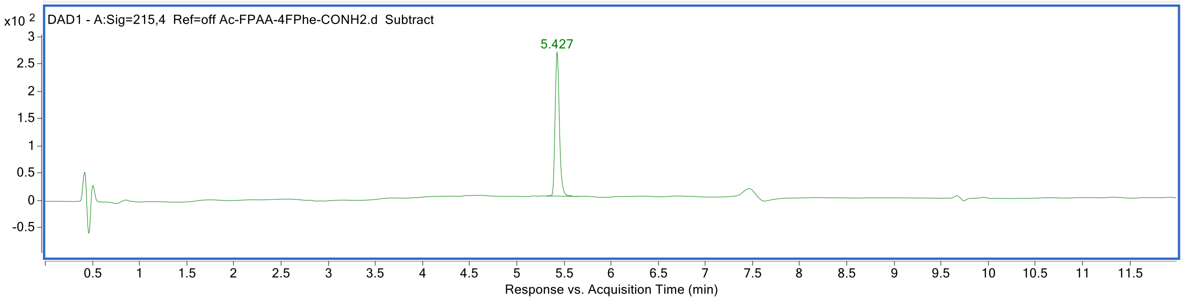

Figure S64. Analytical HPLC.

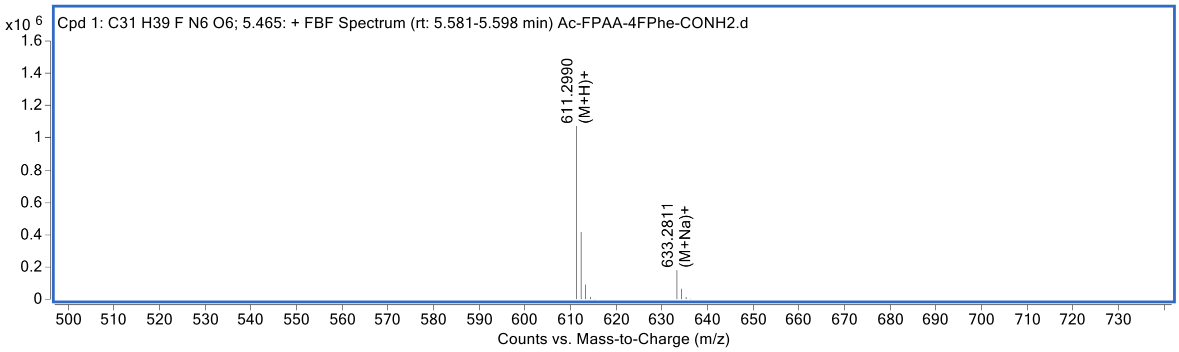

Figure S65. Mass spectrum.

Ac-YPAA(4FPhe)-CONH₂ (Peptide 19)

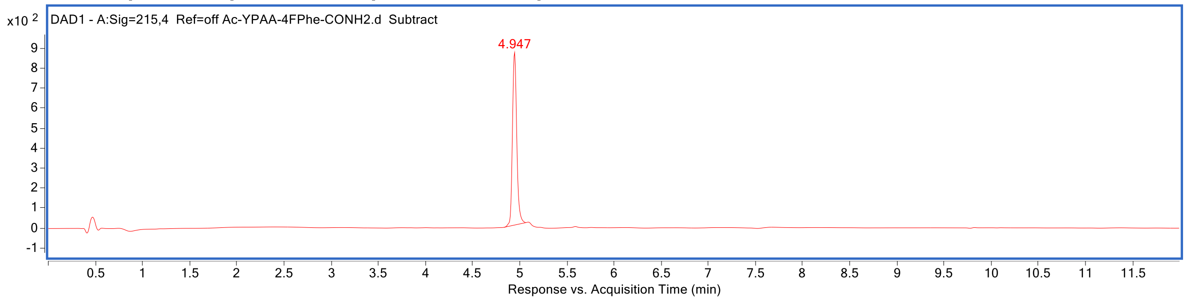

Figure S66. Analytical HPLC.

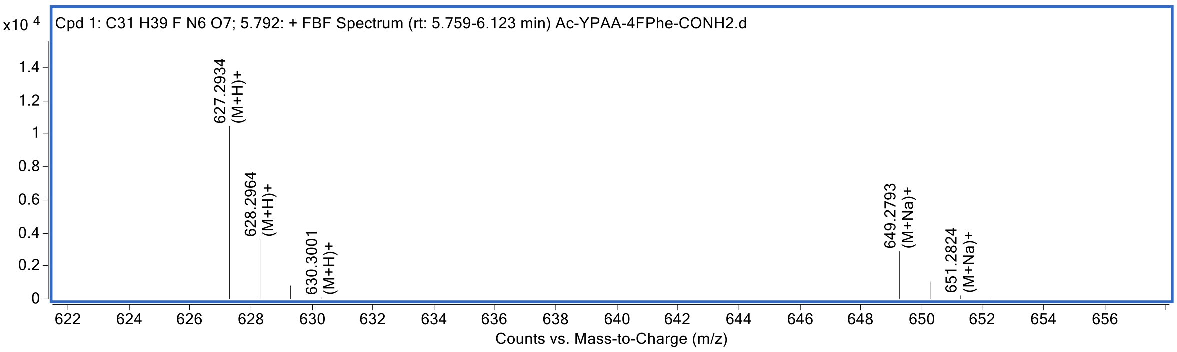

Figure S67. Mass spectrum.

Ac-WPAA(4FPhe)-CONH₂ (Peptide 20)

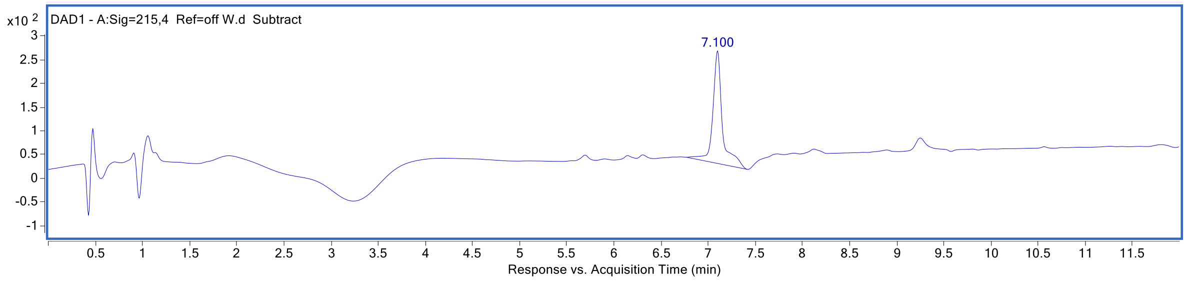

Figure S68. Analytical HPLC.

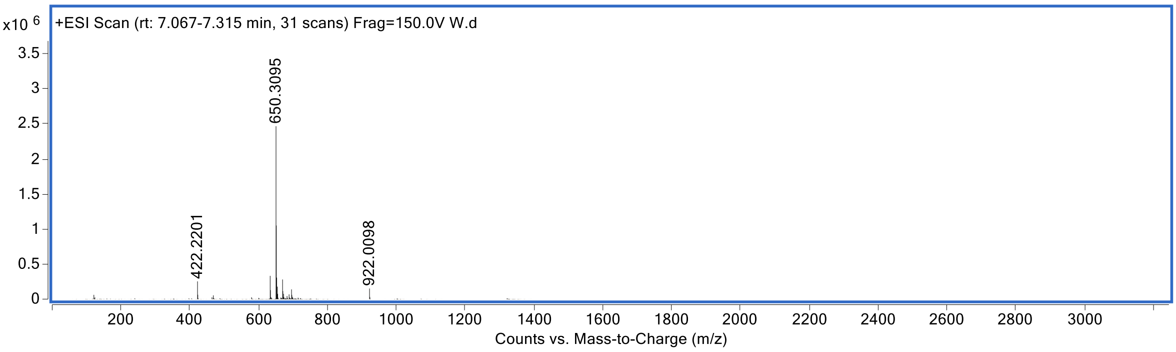

Figure S69. Mass spectrum.

Ac-YPGA(4FPhe)-CONH$_2$ (Peptide 21)

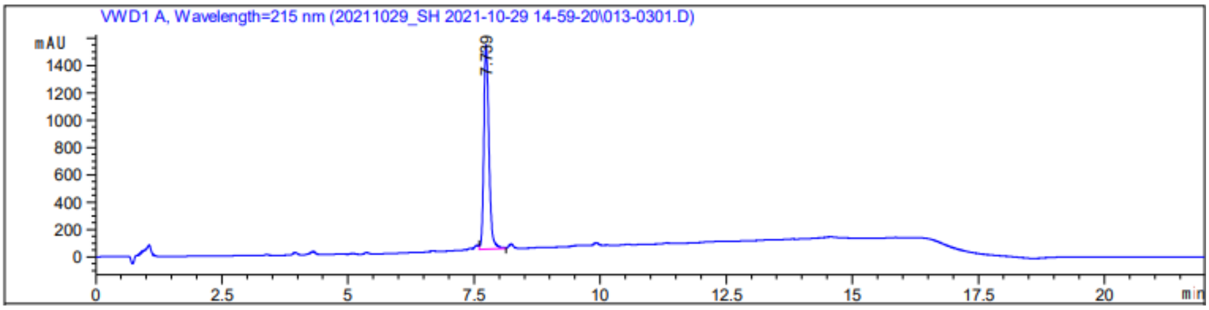

Figure S70. Analytical HPLC.

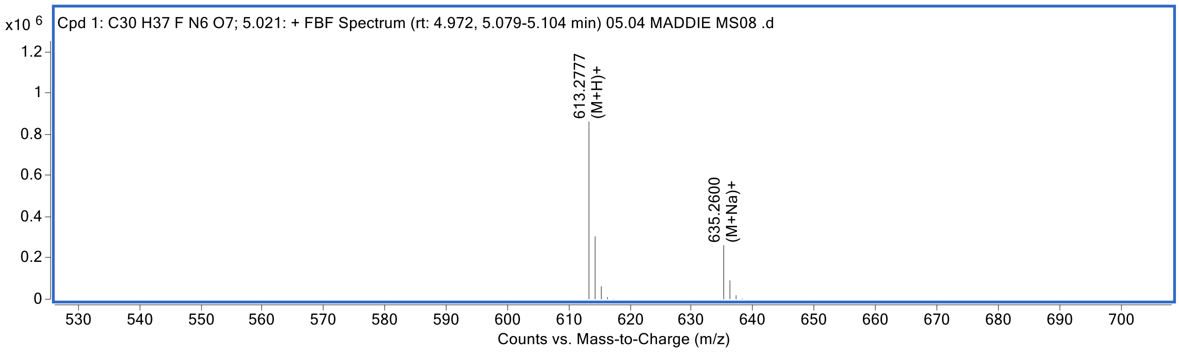

Figure S71. Mass spectrum.

Ac-YPPA(4FPhe)-CONH$_2$ (Peptide 22)

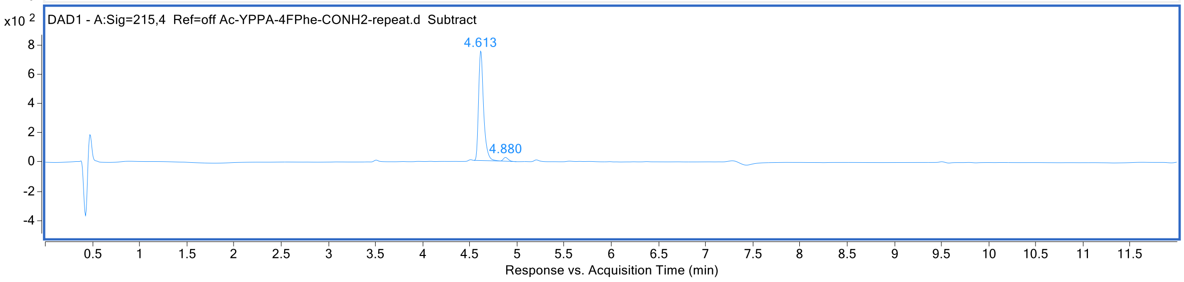

Figure S72. Analytical HPLC.

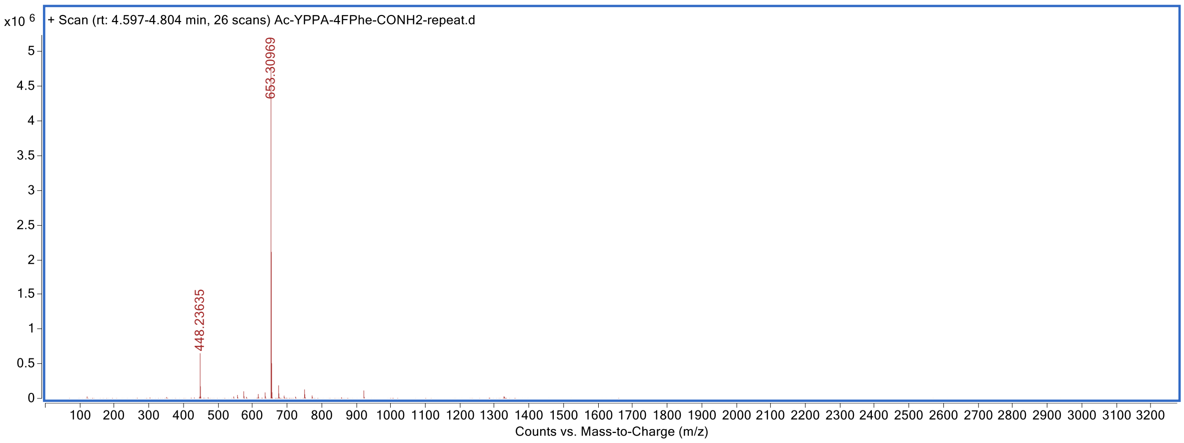

Figure S73. Mass spectrum.

Ac-YPIA(4FPhe)-CONH₂ (Peptide 23)

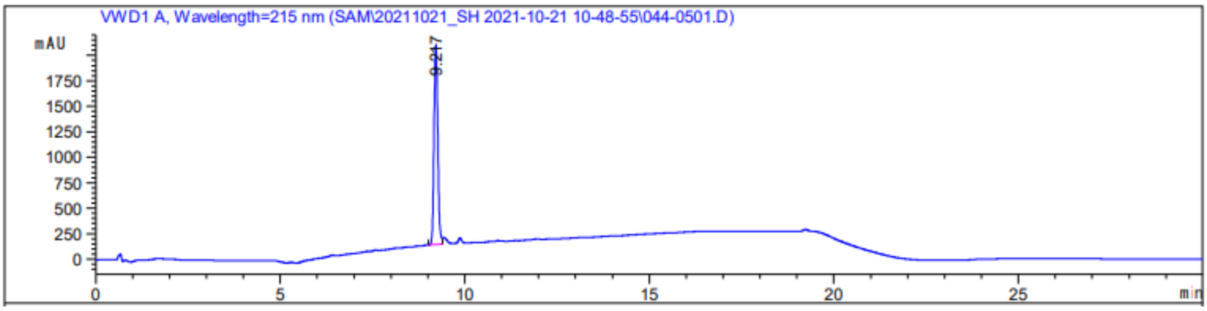

Figure S74. Analytical HPLC.

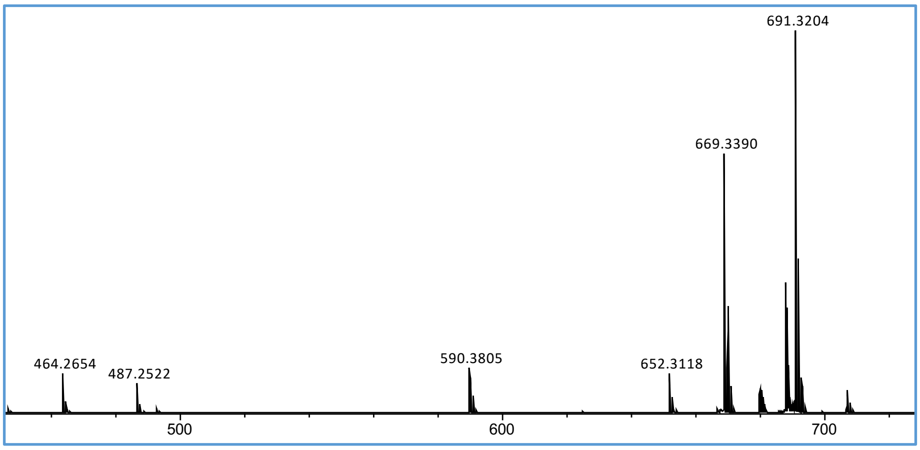

Figure S75. Mass spectrum.

Ac-YPLA(4FPhe)-CONH₂ (Peptide 24)

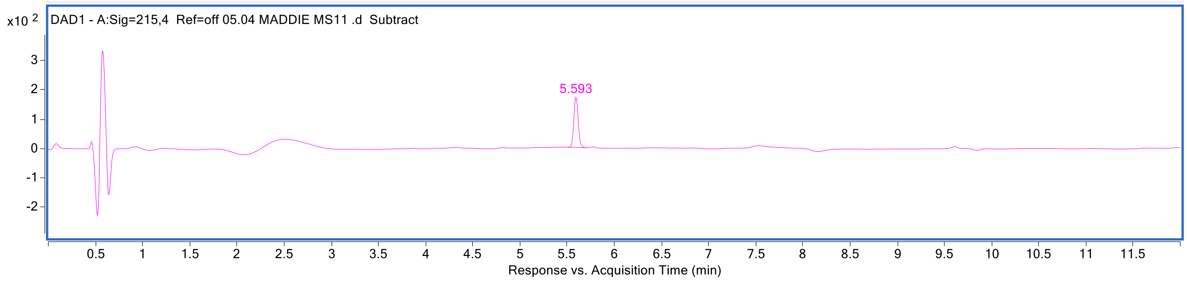

Figure S76. Analytical HPLC.

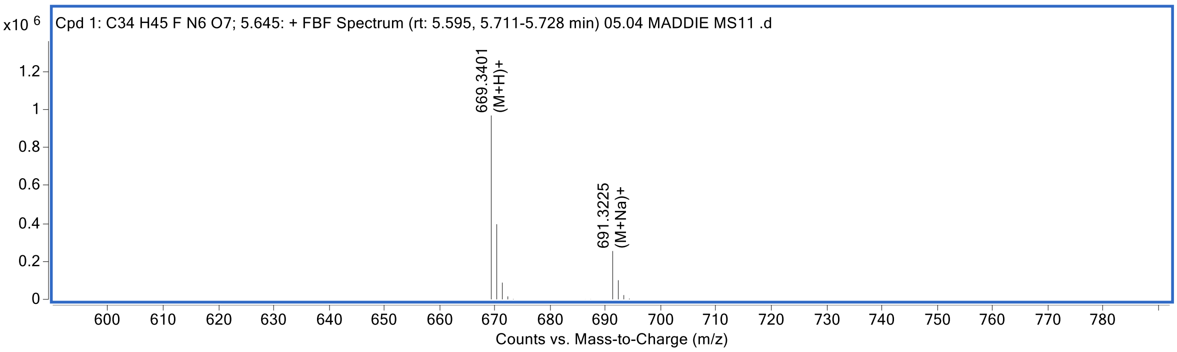

Figure S77. Mass spectrum.

Ac-YPVA(4FPhe)-CONH$_2$ (Peptide 25)

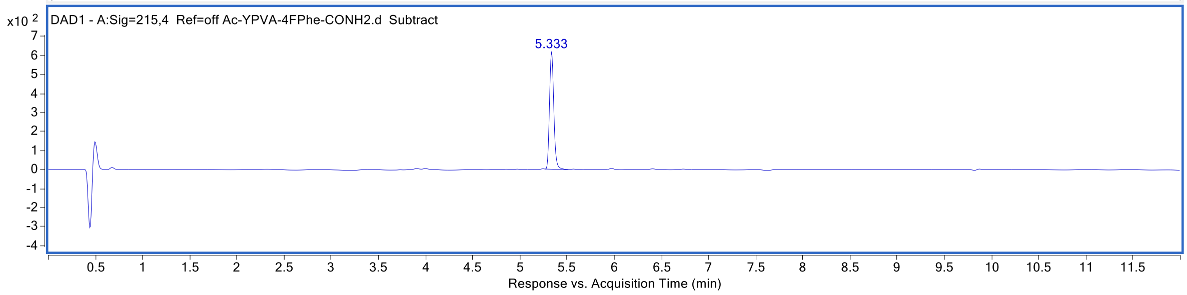

Figure S78. Analytical HPLC.

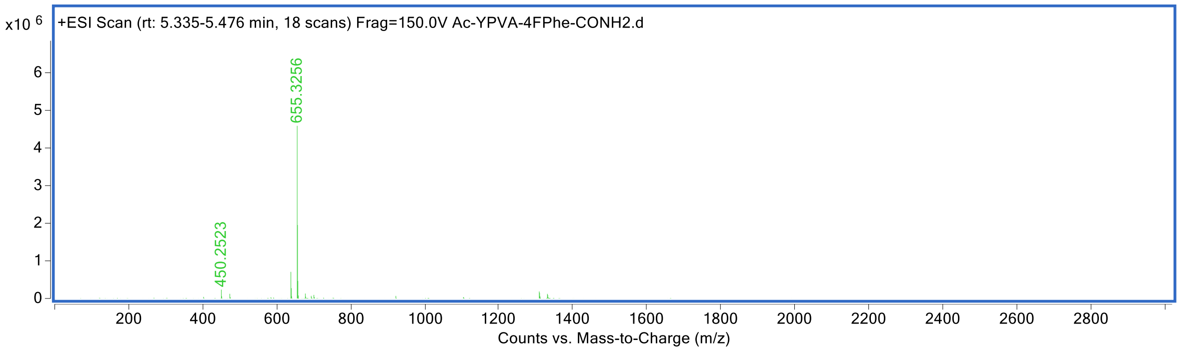

Figure S79. Mass spectrum.

Ac-YPMA(4FPhe)-CONH$_2$ (Peptide 26)

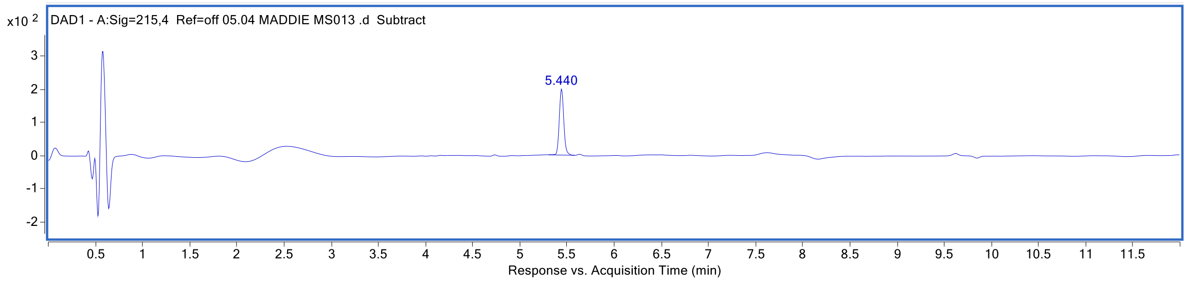

Figure S80. Analytical HPLC.

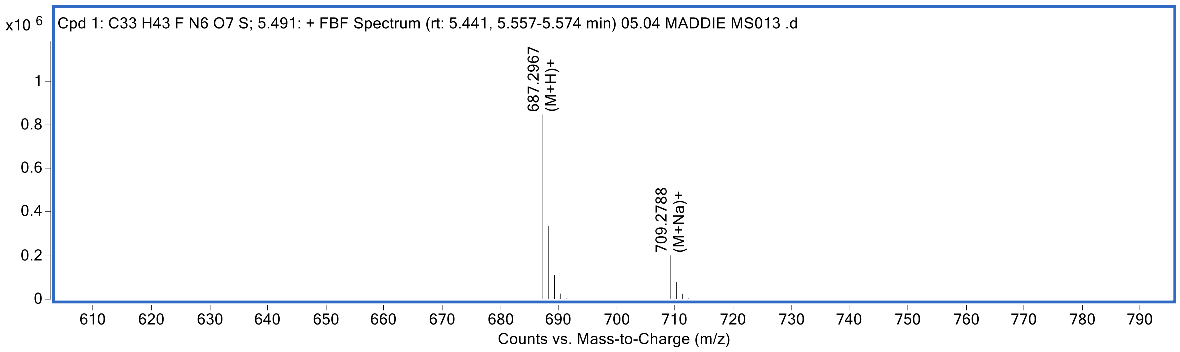

Figure S81. Mass spectrum.

YPCA(4FPhe)-CONH$_2$ (Peptide 27)

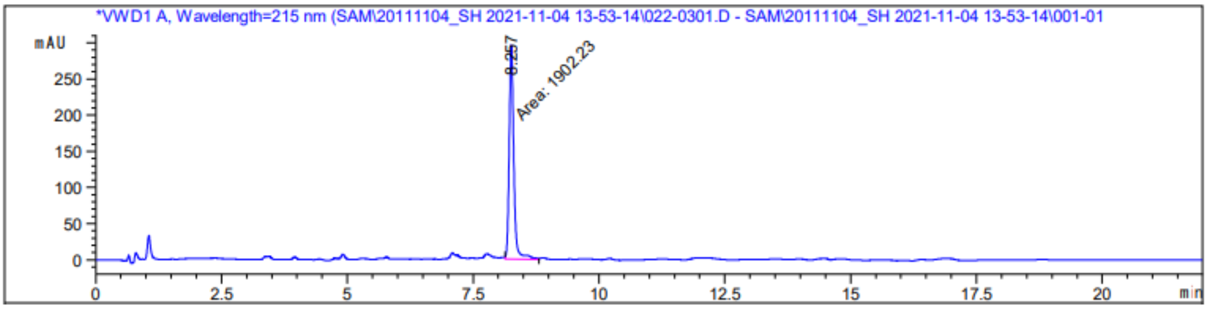

Figure S82. Analytical HPLC.

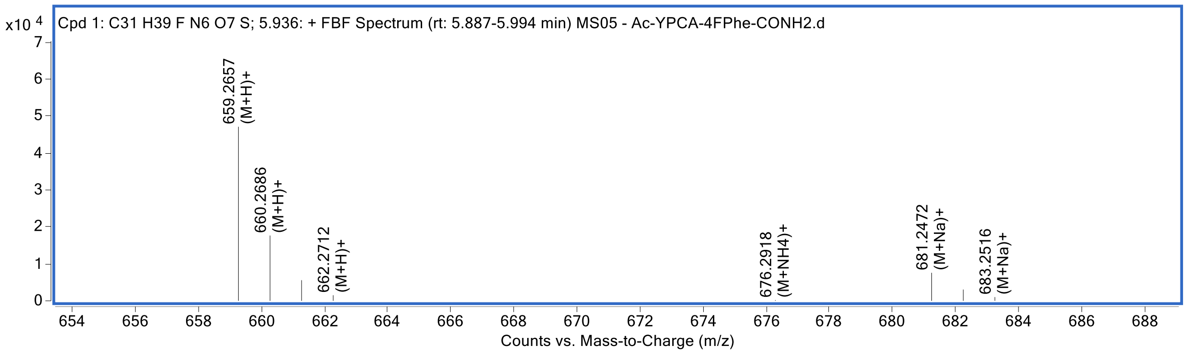

Figure S83. Mass spectrum.

Ac-YPSA(4FPhe)-CONH$_2$ (Peptide 28)

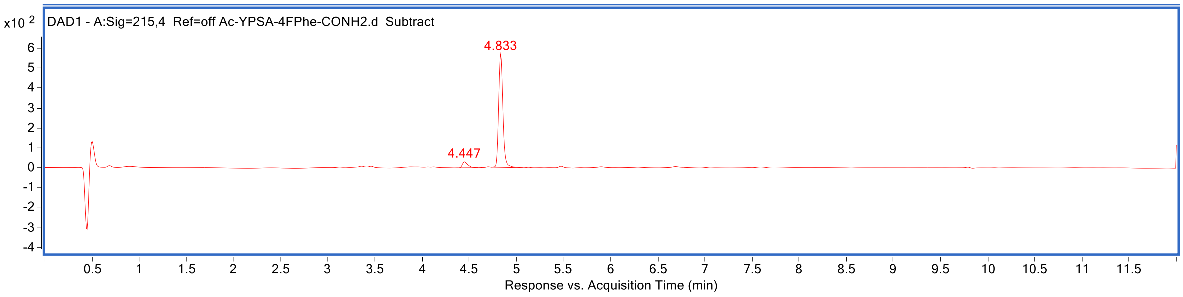

Figure S84. Analytical HPLC.

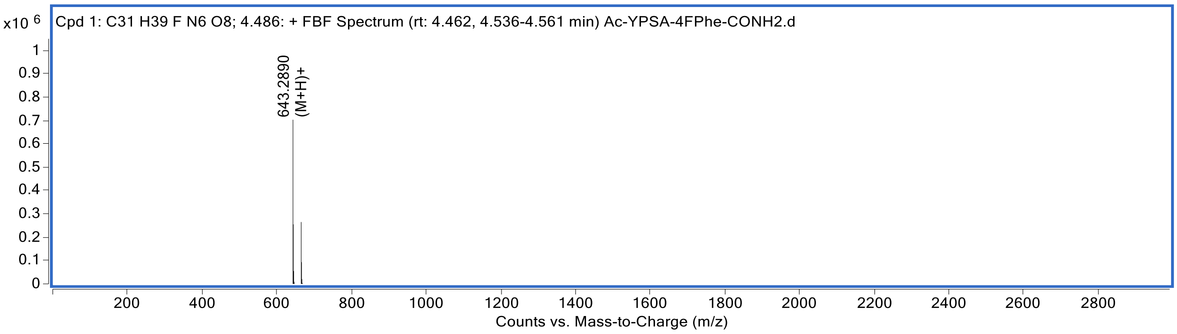

Figure S85. Mass spectrum.

Ac-YPTA(4FPhe)-CONH$_2$ (Peptide 29)

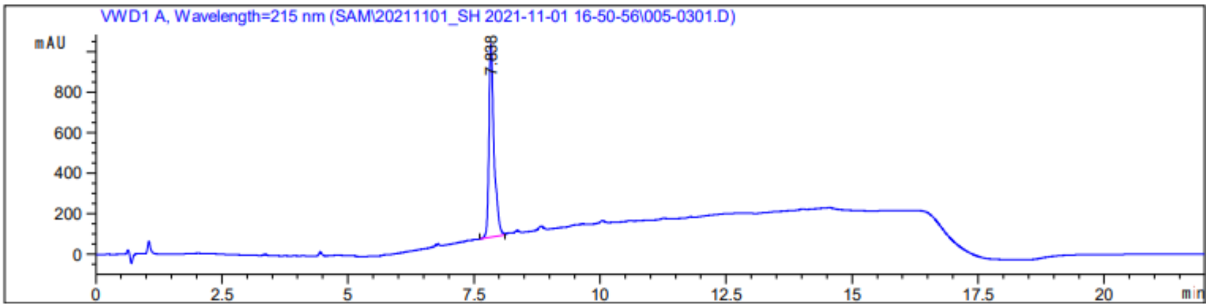

Figure S86. Analytical HPLC.

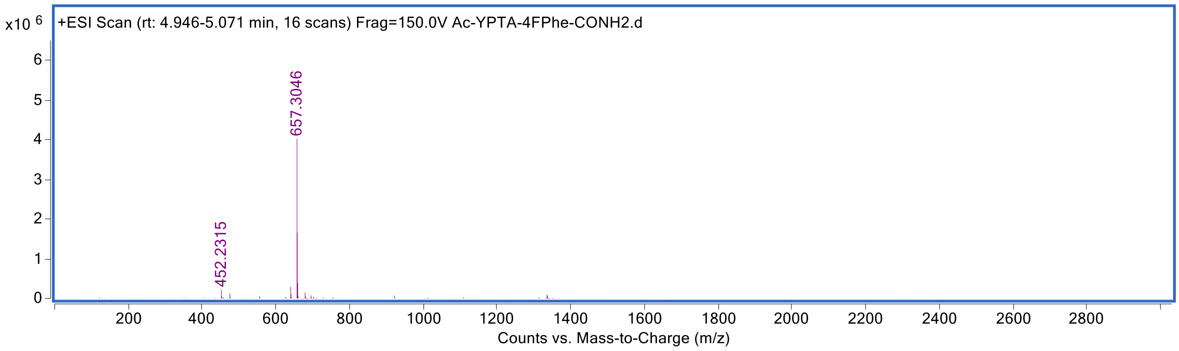

Figure S87. Mass spectrum.

Ac-YPDA(4FPhe)-CONH$_2$ (Peptide 30)

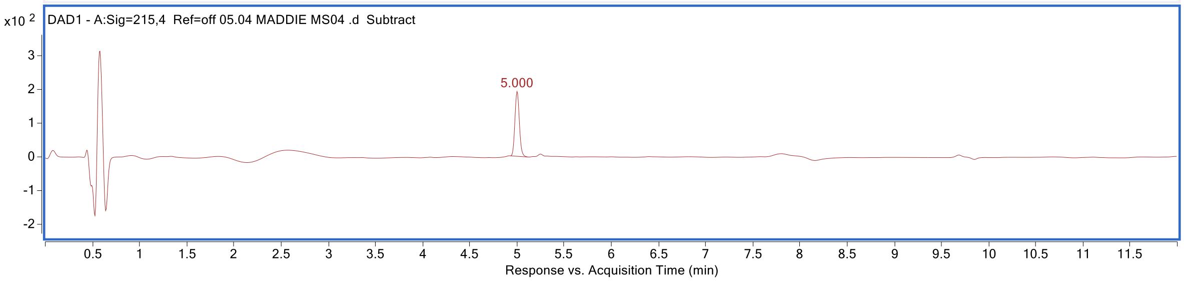

Figure S88. Analytical HPLC.

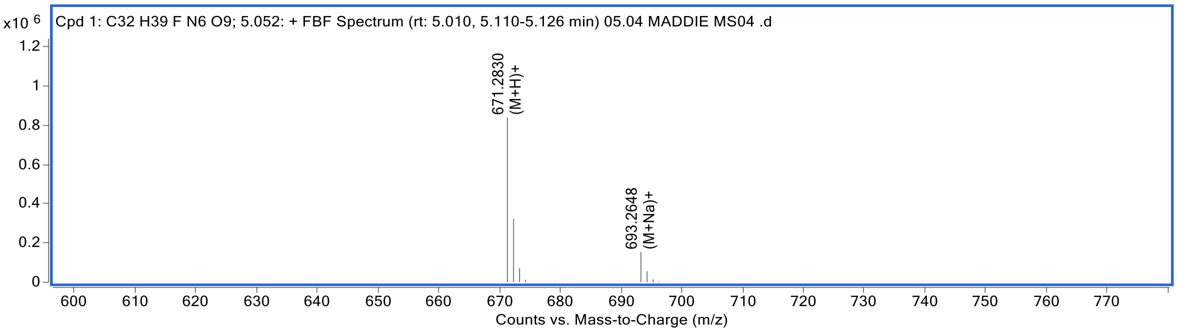

Figure S89. Mass spectrum.

Ac-YPEA(4FPhe)-CONH$_2$ (Peptide 31)

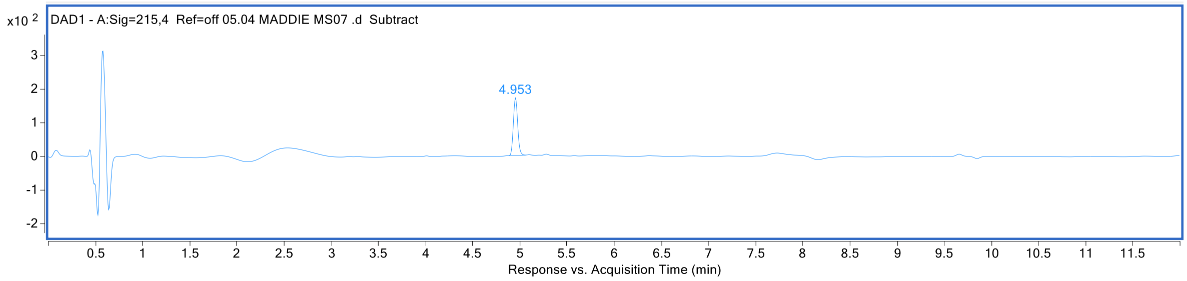

Figure S90. Analytical HPLC.

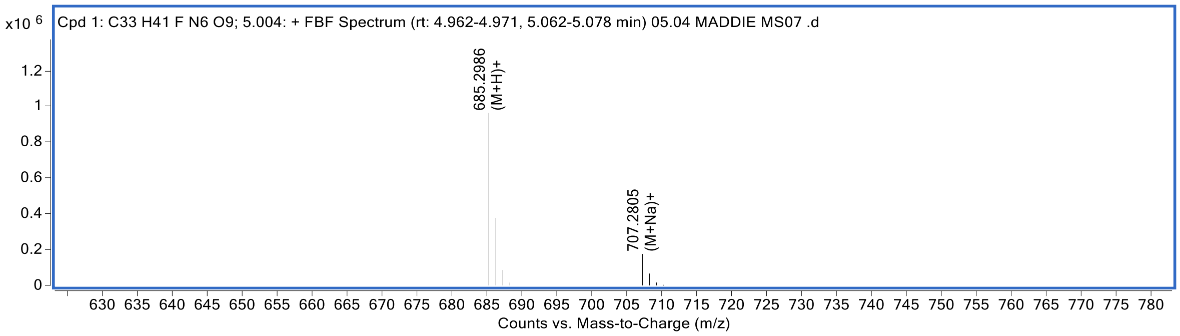

Figure S91. Mass spectrum.

Ac-YPNA(4FPhe)-CONH$_2$ (Peptide 32)

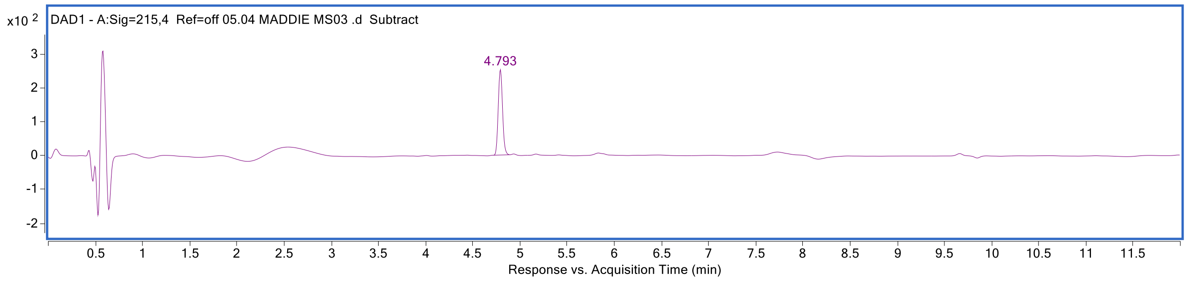

Figure S92. Analytical HPLC.

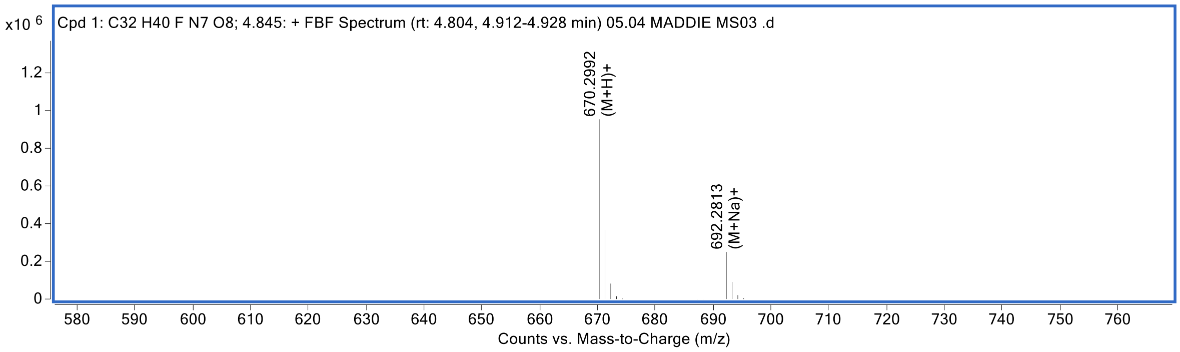

Figure S93. Mass spectrum.

Ac-YPQA(4FPhe)-CONH$_2$ (Peptide 33)

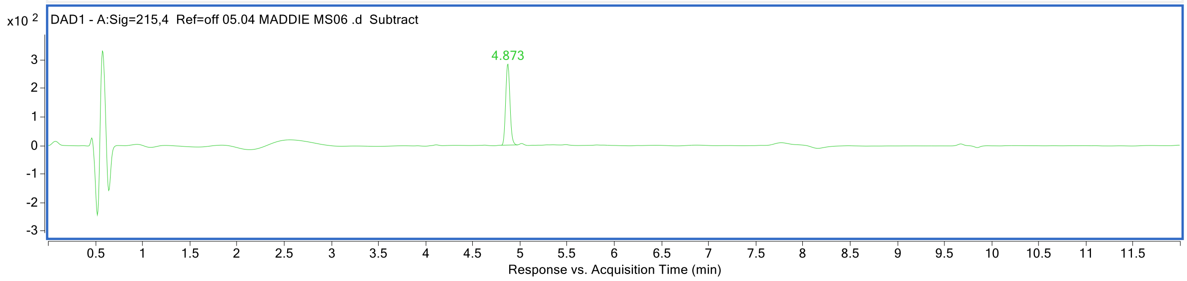

Figure S94. Analytical HPLC.

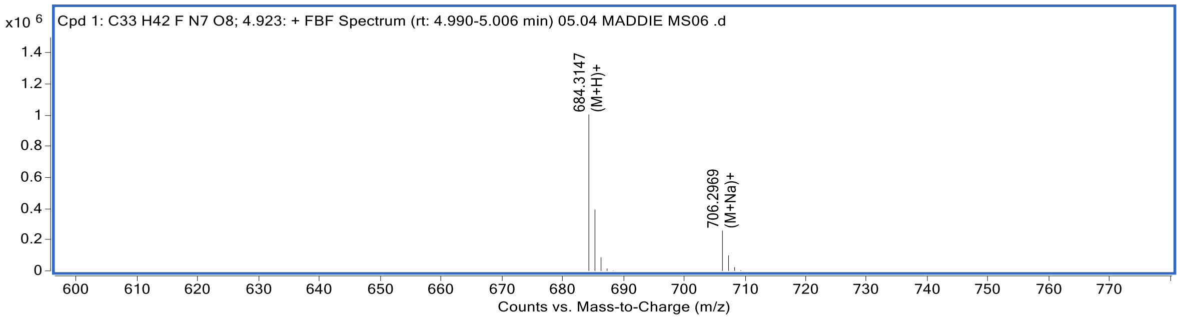

Figure S95. Mass spectrum.

Ac-YPRA(4FPhe)-CONH$_2$ (Peptide 34)

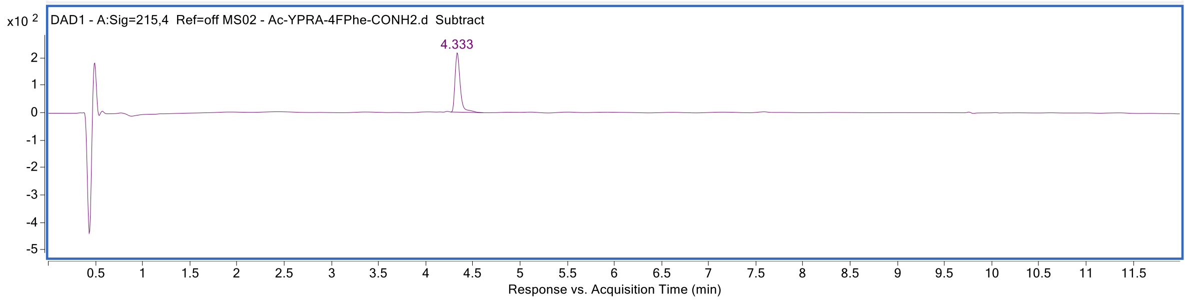

Figure S96. Analytical HPLC.

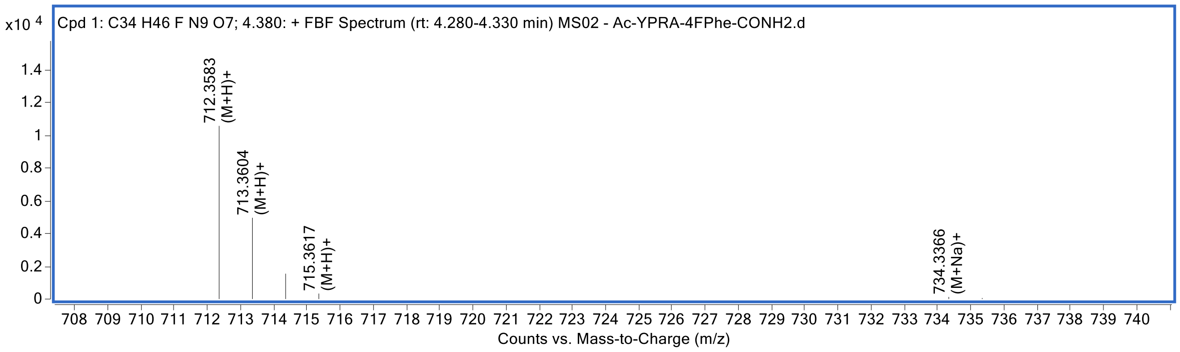

Figure S97. Mass spectrum.

Ac-YPKA(4FPhe)-CONH$_2$ (Peptide 35)

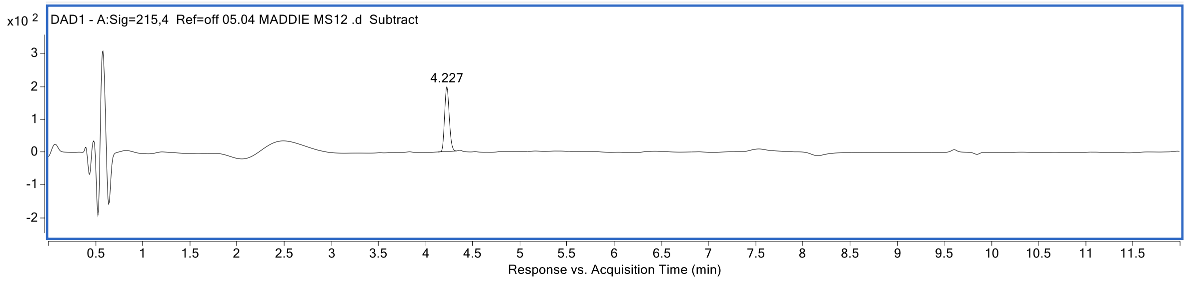

Figure S98. Analytical HPLC.

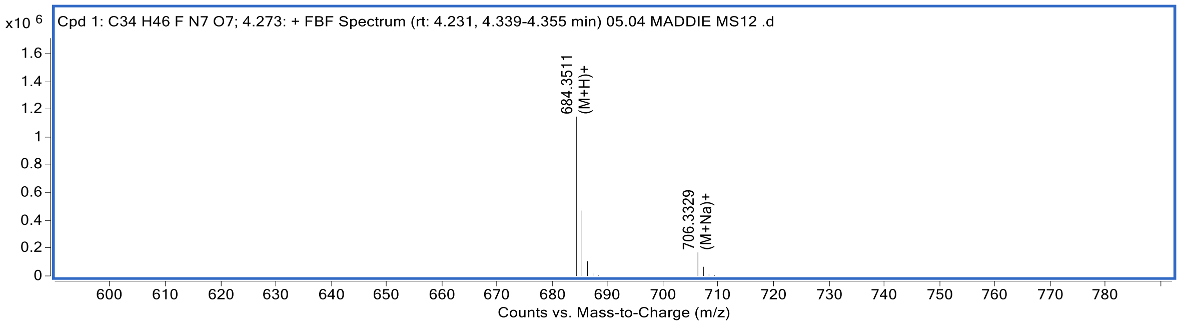

Figure S99. Mass spectrum.

Ac-YPHA(4FPhe)-CONH$_2$ (Peptide 36)

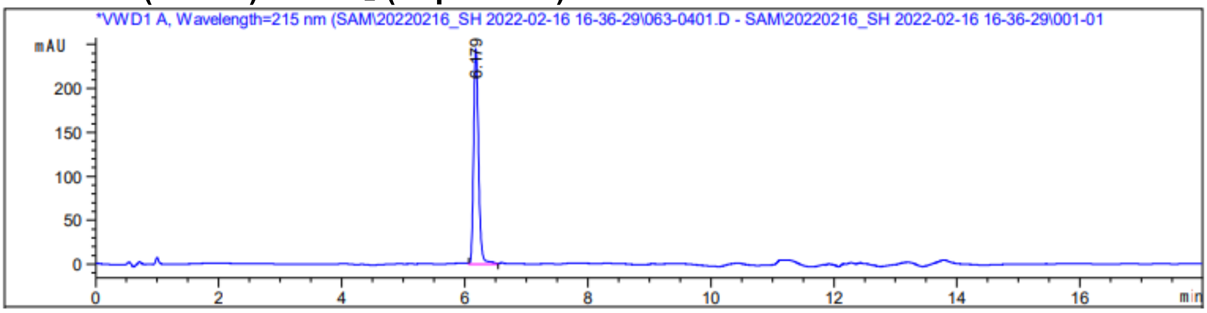

Figure S100. Analytical HPLC.

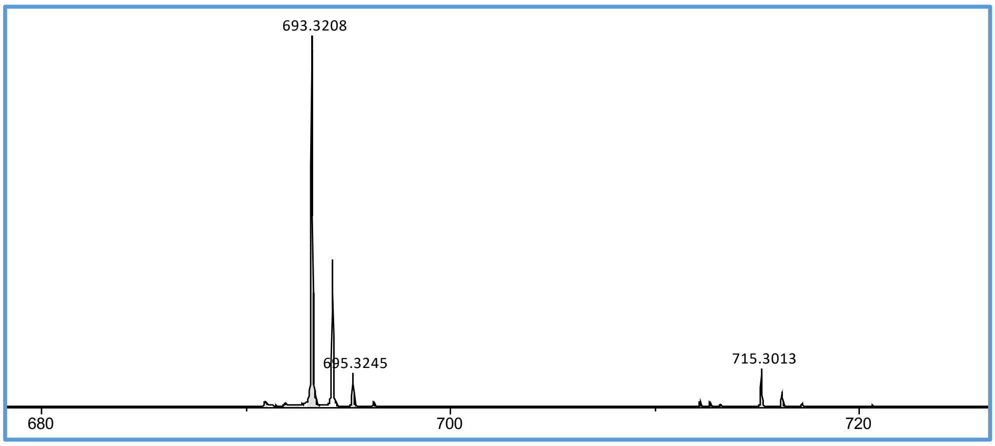

Figure S101. Mass spectrum.

Ac-YPFA(4FPhe)-CONH$_2$ (Peptide 37)

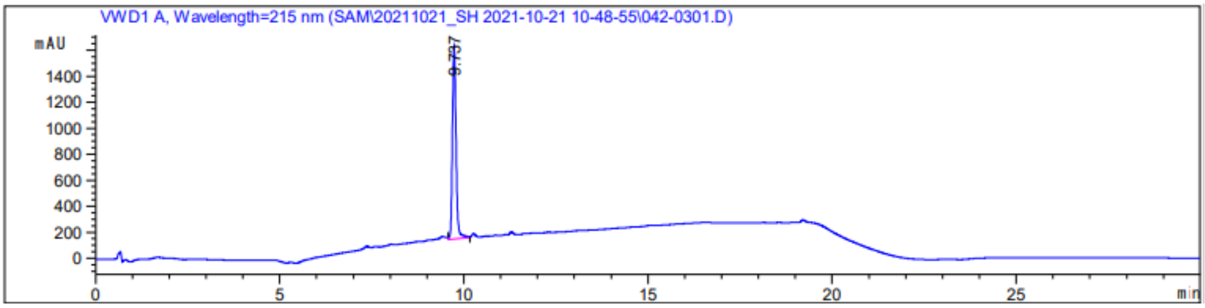

Figure S102. Analytical HPLC.

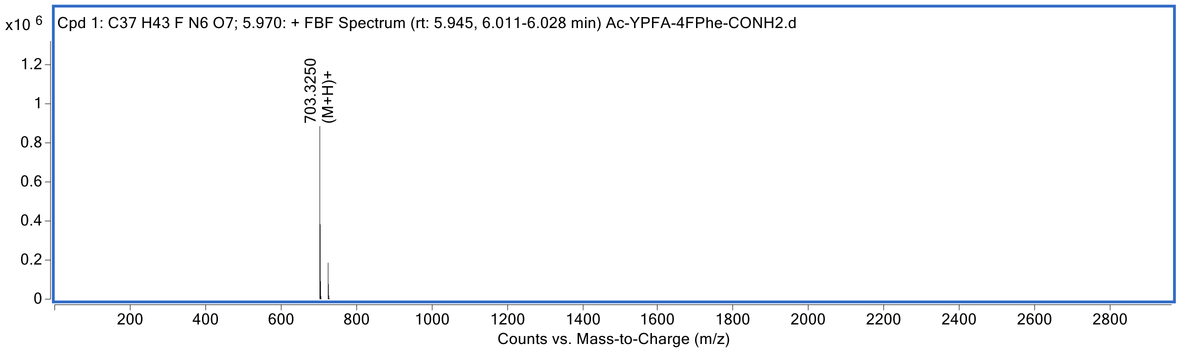

Figure S103. Mass spectrum.

Ac-YPYA(4FPhe)-CONH$_2$ (Peptide 38)

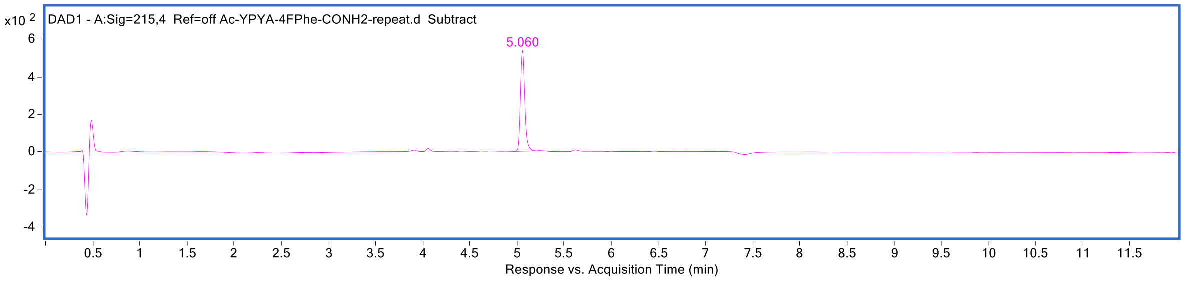

Figure S104. Analytical HPLC.

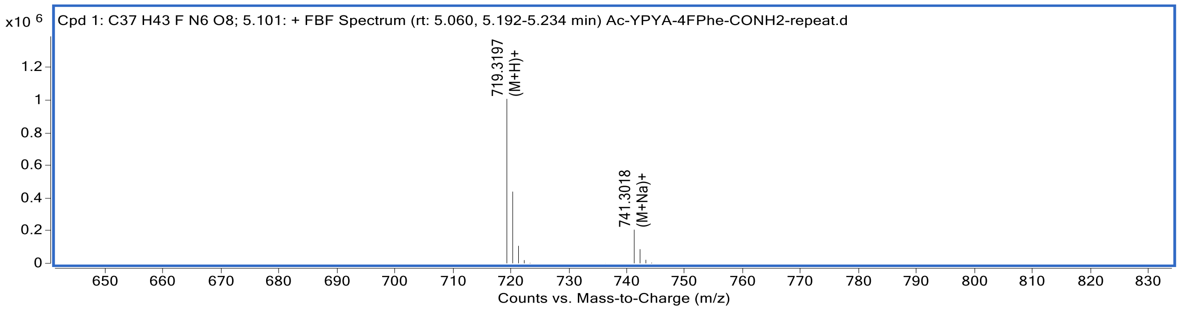

Figure S105. Mass spectrum.

Ac-YPWA(4FPhe)-CONH$_2$ (Peptide 39)

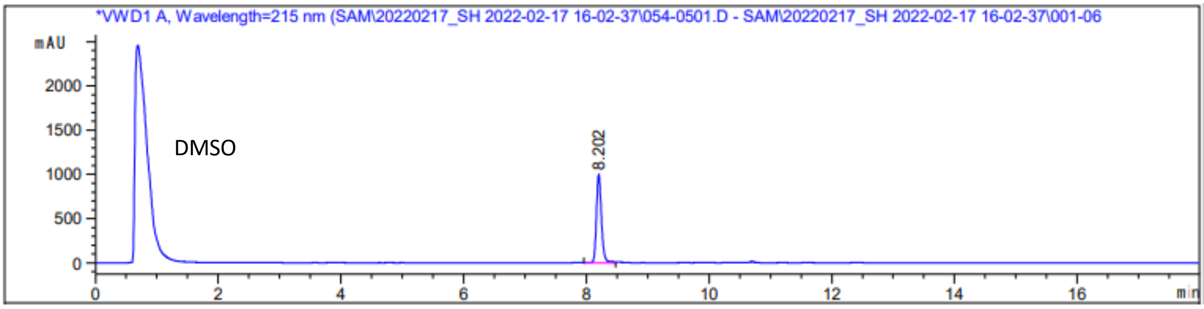

Figure S106. Analytical HPLC.

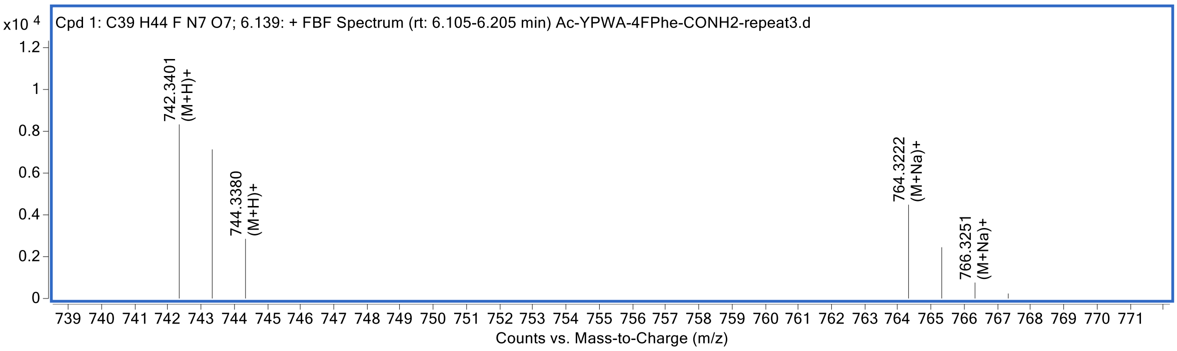

Figure S107. Mass spectrum.

Ac-YPDD(4F-Phe)-CONH$_2$ (Peptide 40)

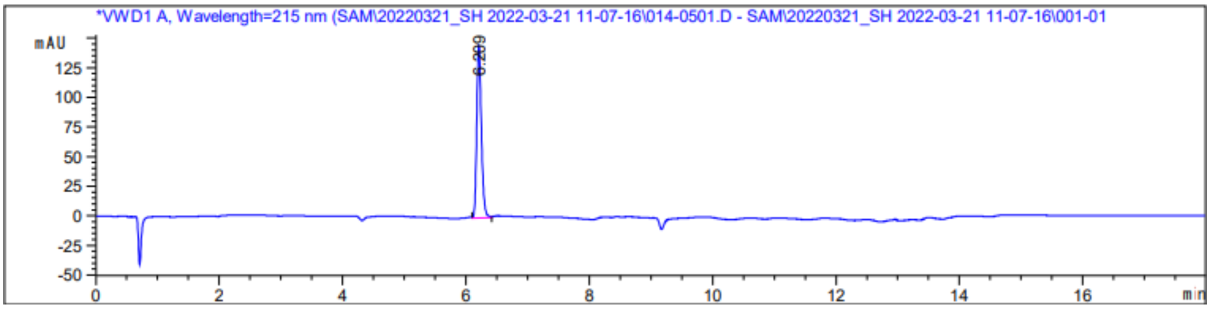

Figure S108. Analytical HPLC.

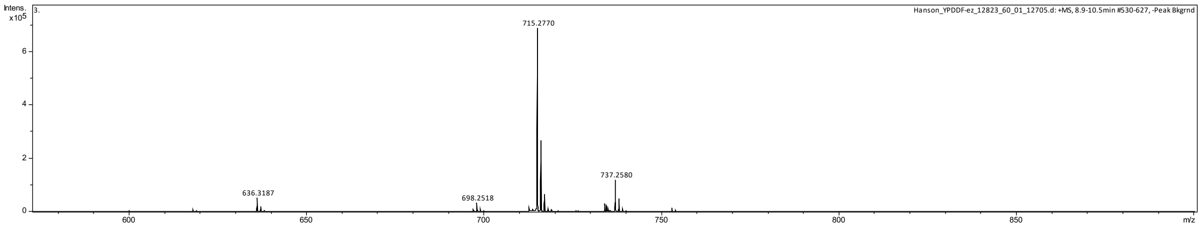

Figure S109. Mass spectrum.

Ac-YPHD(4FPhe)-$CONH_2$ (Peptide 41)

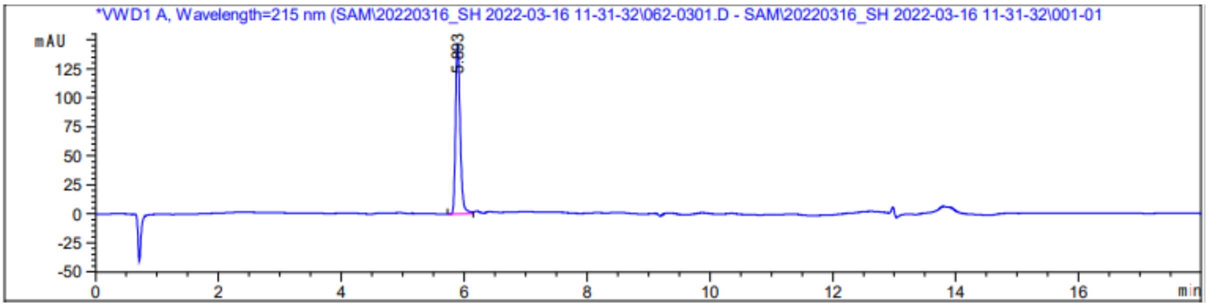

Figure S110. Analytical HPLC.

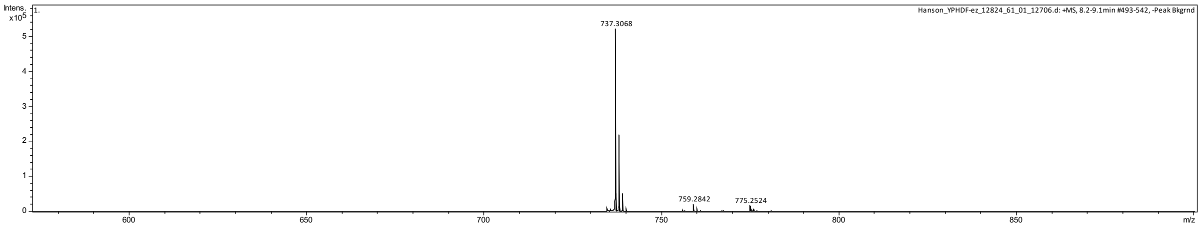

Figure S111. Mass spectrum.

Ac-YPKD(4FPhe)-$CONH_2$ (Peptide 42)

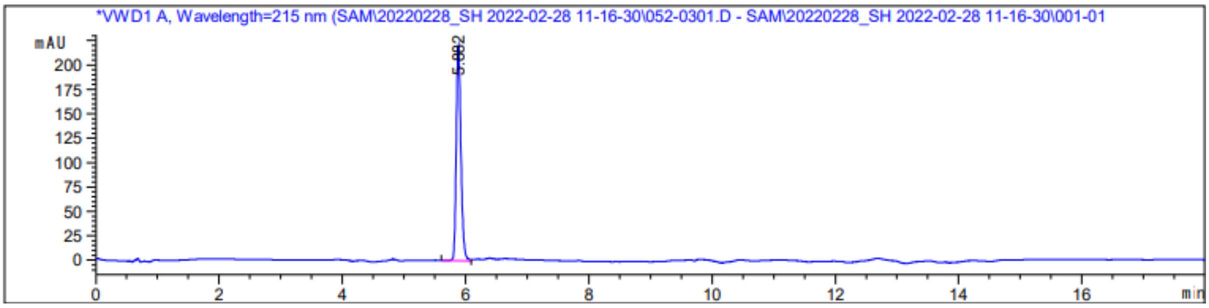

Figure S112. Analytical HPLC.

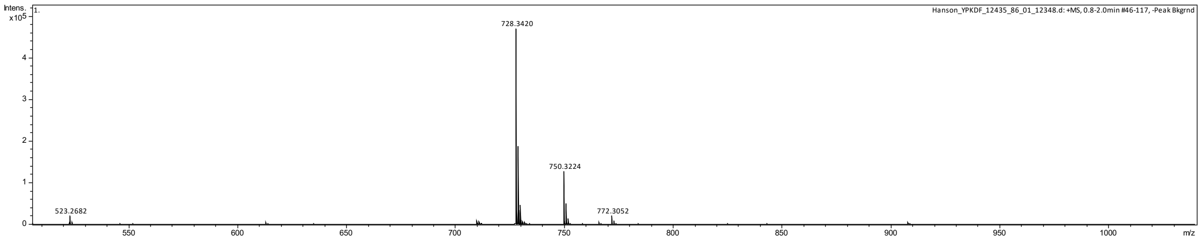

Figure S113. Mass spectrum.

Ac-YPRD(4FPhe)-CONH$_2$ (Peptide 43)

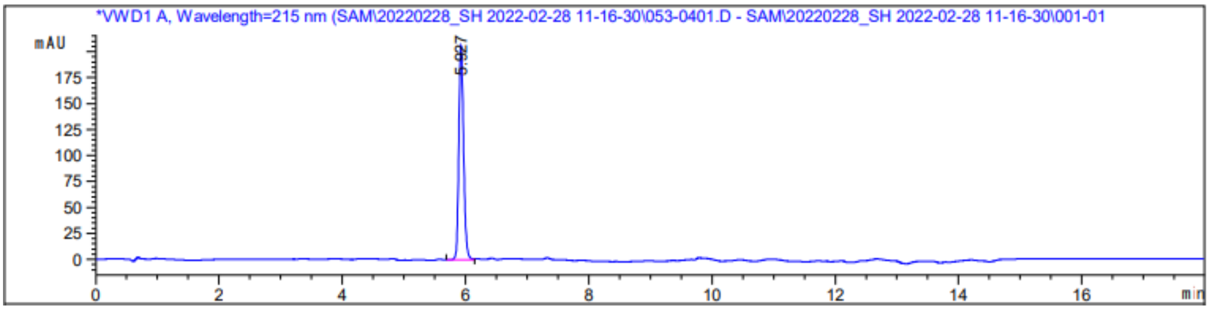

Figure S114. Analytical HPLC.

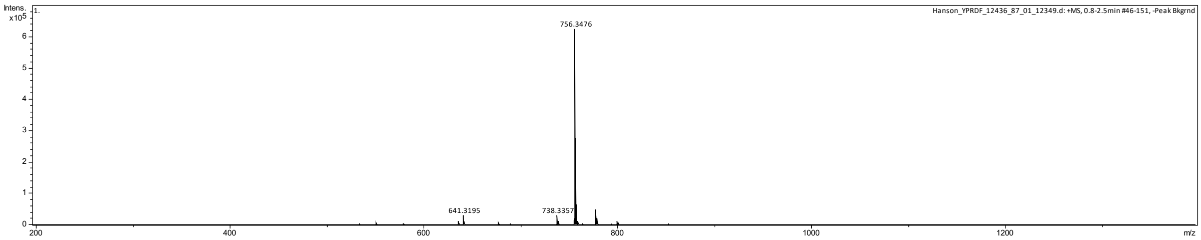

Figure S115. Mass spectrum.

Ac-APQE(4FPhe)-CONH$_2$ (Peptide 44)

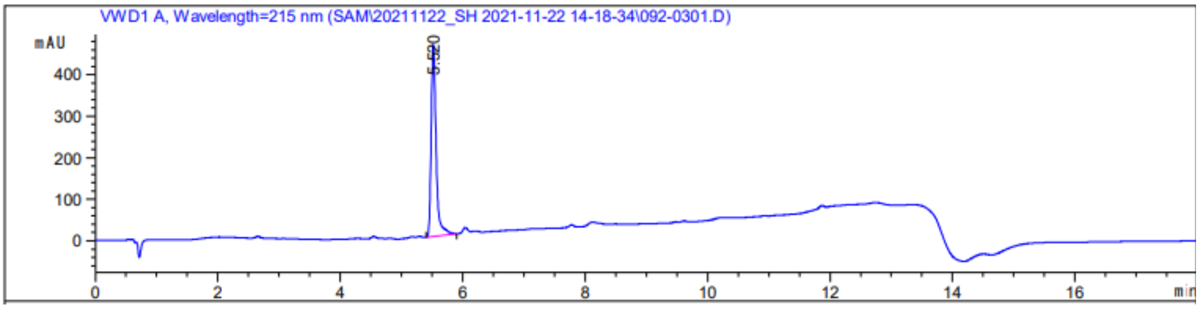

Figure S116. Analytical HPLC.

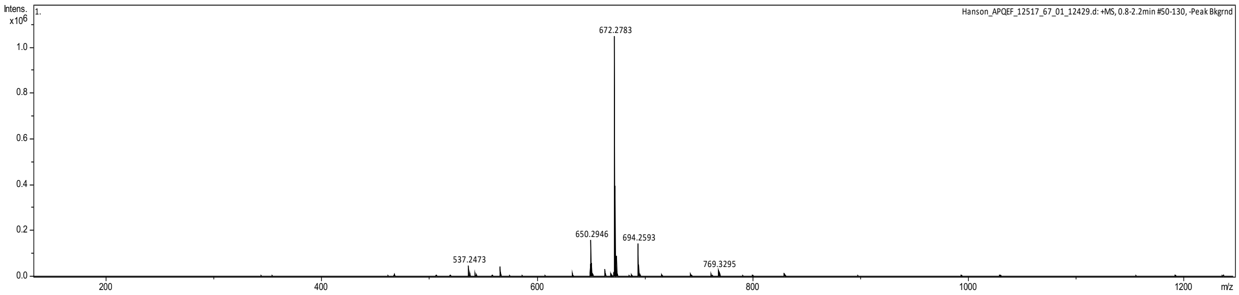

Figure S117. Mass spectrum.

Ac-MPVD(4FPhe)-CONH$_2$ (Peptide 45)

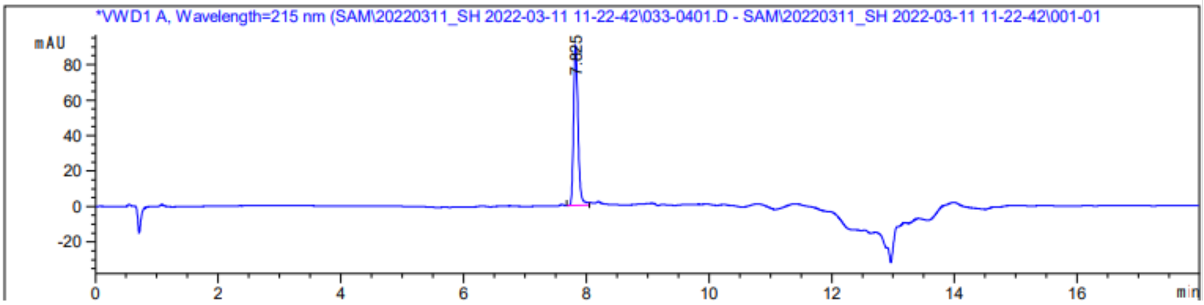

Figure S118. Analytical HPLC.

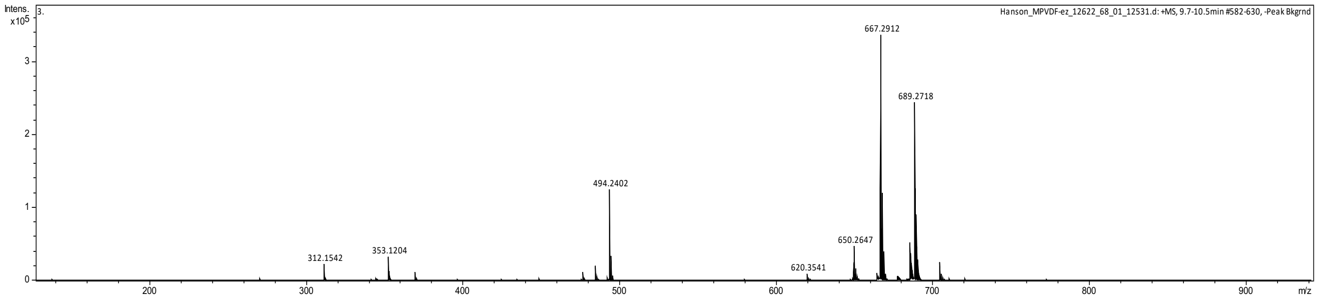

Figure S119. Mass spectrum.

Ac-DPDN(4FPhe)-CONH$_2$ (Peptide 46)

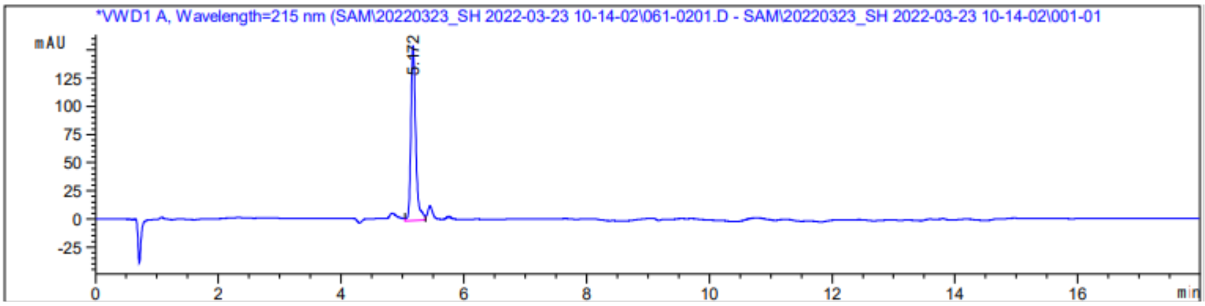

Figure S120. Analytical HPLC.

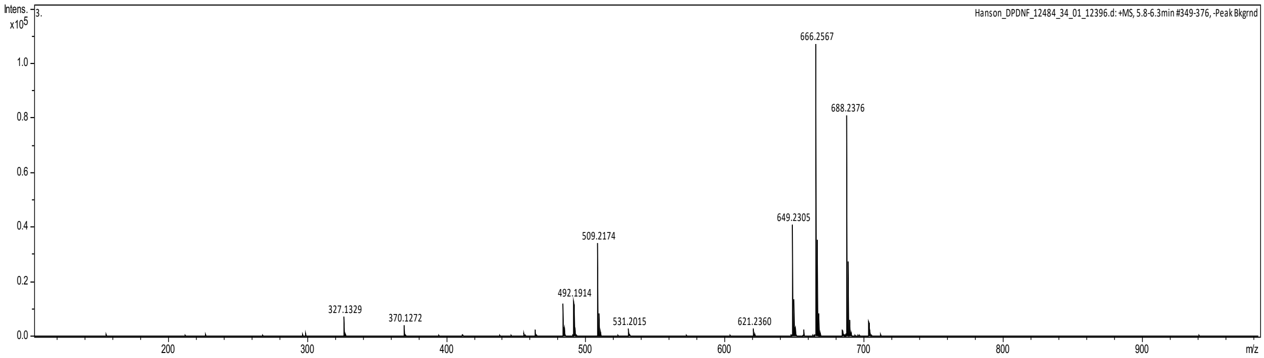

Figure S121. Mass spectrum.

Ac-MPSE(4FPhe)-$CONH_2$ (Peptide 47)

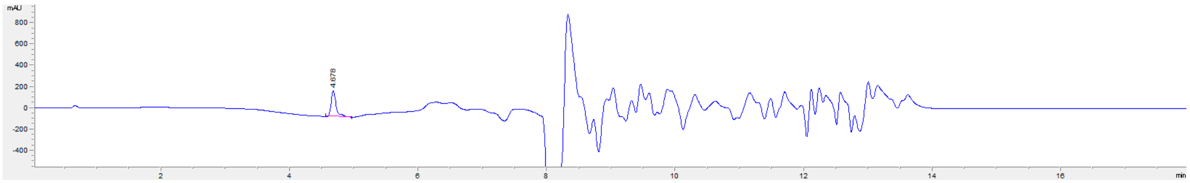

Figure S122. Analytical HPLC.

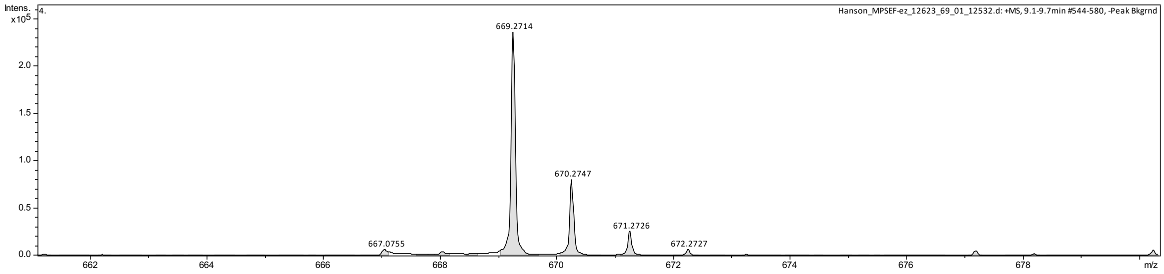

Figure S123. Mass spectrum.

Ac-EPEA(4FPhe)-$CONH_2$ (Peptide 48)

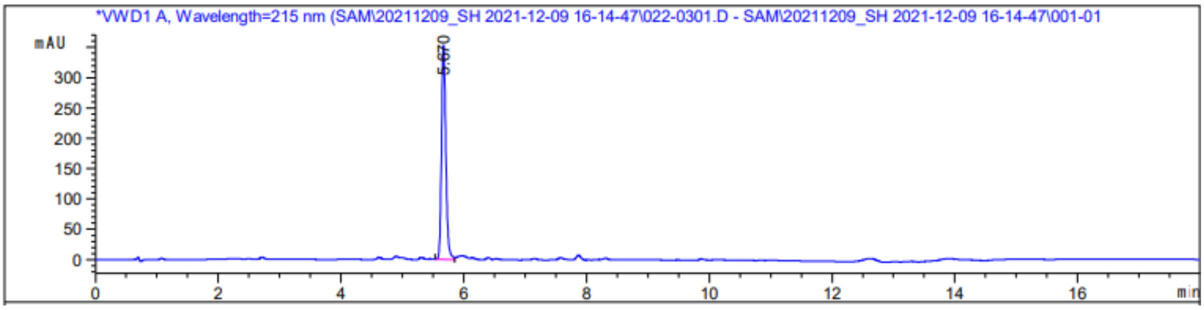

Figure S124. Analytical HPLC.

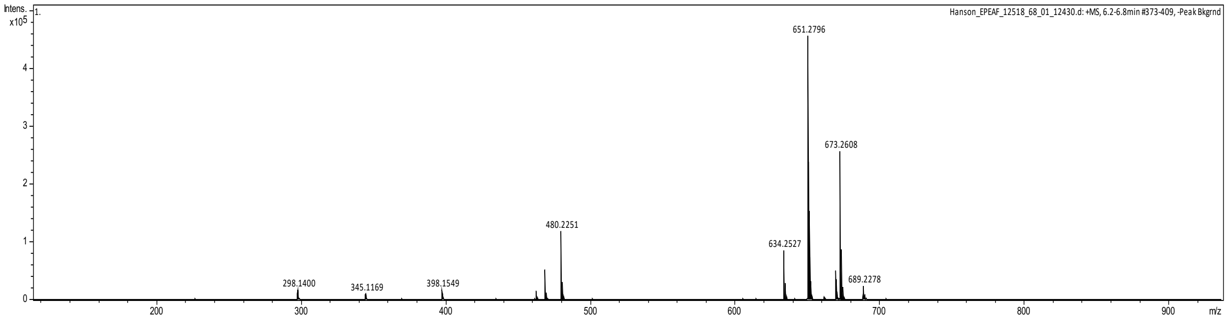

Figure S125. Mass spectrum.

References

1. H. J. Dyson, M. Rance, R. A. Houghten, R. A. Lerner, P. E. Wright, *J. Mol. Biol.* **1988**, *201, 161-200.*

2. C., Renner, S. Alefelder, J. H. Bae, N. Budisa, R. Huber, L. Moroder, *Angew. Chem. Int. Ed.*, **2001**, *40*, 923-925

3. D. P. Raleigh, P. A. Evans, M. Pitkeathly, C. M. Dobson, *J. Mol. Biol*. **1992**, *228*, 338-342.
